# 1,3-Dipolar Cycloaddition of Nitrile Oxides and Nitrilimines to (−)-β-Caryophyllene: Stereoselective Synthesis of Polycyclic Derivatives and Their Biological Testing

**DOI:** 10.3390/ijms252111435

**Published:** 2024-10-24

**Authors:** Dmitry E. Shybanov, Maxim E. Kukushkin, Yuri K. Grishin, Vitaly A. Roznyatovsky, Viktor A. Tafeenko, Louay Abo Qoura, Vadim S. Pokrovsky, Olga I. Yarovaya, Svetlana V. Belyaevskaya, Alexandrina S. Volobueva, Iana L. Esaulkova, Vladimir V. Zarubaev, Elena K. Beloglazkina

**Affiliations:** 1 Department of Chemistry, M. V. Lomonosov Moscow State University, 119991 Moscow, Russia; deshyb@mail.ru (D.E.S.); lemminng@mail.ru (M.E.K.); ykgris@mail.ru (Y.K.G.); vit.rozn@nmr.chem.msu.ru (V.A.R.); tafeenko-victor@yandex.ru (V.A.T.); 2 Research Institute of Molecular and Cellular Medicine, People’s Friendship University of Russia (RUDN University), 117198 Moscow, Russia; louay.ko@gmail.com (L.A.Q.); vadimpokrovsky@gmail.com (V.S.P.); 3 N.N. Blokhin National Medical Research Center of Oncology, Ministry of Health of Russian Federation, 115478 Moscow, Russia; 4 N.N. Vorozhtsov Novosibirsk Institute of Organic Chemistry, Siberian Branch, Russian Academy of Sciences, Lavrentjev Avenue 9, 630090 Novosibirsk, Russia; ooo@nioch.nsc.ru; 5 Pasteur Research Institute of Epidemiology and Microbiology, 14 MiraStr, 197101 St. Petersburg, Russiazarubaev@gmail.com (V.V.Z.)

**Keywords:** 1,3-dipolar cycloaddition, caryophyllene, nitrile oxides, nitrilimines, antiviral activity, cytotoxicity

## Abstract

The cycloaddition of nitrile oxides and nitrilimines to one or both of the C=C double bonds of caryophyllene is described. The possibility of introducing five-membered fused and spiro-linked heterocycles into the structure of sesquiterpenes by the 1,3-dipolar cycloaddition reactions of nitrile oxides and nitrilimines to caryophyllene was demonstrated. As a result of these reactions, pharmacophore fragments of isoxazoline and pyrazoline are introduced into the structure of caryophyllene, which leads to an increase in the conformational rigidity of the molecule. A complete stereochemical assignment of 1,3-dipolar cycloaddition adducts to caryophyllene was carried out. The study of antiviral and cytotoxic activity for some heterocyclic derivatives synthesized in this work revealed relatively high biological activity of previously little-studied cycloaddition adducts at the exocyclic C=CH_2_ bond of caryophyllene. The effect of substituents in the synthesized heterocycles on biological activity was demonstrated. Compounds with a good inhibitory effect on the H1N1 influenza virus were revealed. The activity of the compound was demonstrated up to 6 h post infection, and this could be due to slight inhibiting activity against viral neuraminidase, necessary at the stage of progeny virion budding.

## 1. Introduction

Currently, the synthesis of biologically active molecules based on natural compounds is a relevant area of medicinal chemistry. Natural products have played a key role in drug discovery, especially for cancer and infectious diseases, because they are structurally “optimized” by evolution to serve particular biological functions [1]; in addition, natural compounds are isolated from natural sources in enantiomerically pure form and already contain a set of unique structural fragments, the creation of which, by synthetic chemistry methods, as a rule, requires multi-step synthetic procedures [1,2,3]. The availability and renewability of natural raw materials reduces the cost of the potential final molecule and makes it possible to easily scale up the synthesis to an industrial scale.

**Figure 1:**
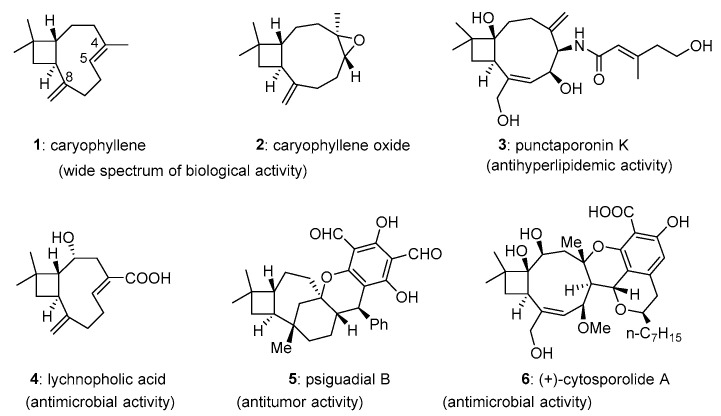
Examples of biologically active caryophyllene derivatives [7,17,20,29,30].

The natural terpene (−)-β-caryophyllene (Figure 1, compound **1**) is a sesquiterpene with a *trans*-cyclononene cycle fused to a four-membered carbocycle. Caryophyllene is the most common member of the bicyclic sesquiterpene family and it is found in some essential oils, particularly *Eugenia caryophyllata* [4], *Myrica gale* [5], and *Comptonia peregrina* oils [6]. Sesquiterpene hydrocarbon (*E*)-β-caryophylene is one of the most thoroughly studied and promising natural molecules [7]. In recent years, its modulating and pharmacological effects have been demonstrated on various organs, such as the liver [8], kidneys [9], and brain [10]. Antiviral activity of β-caryophyllene has been identified against different enveloped viruses, such as the herpes simplex virus, Newcastle disease virus, and avian infectious bronchitis virus (a gammacoronavirus) [11,12]. Caryophyllene and essential oils containing β-caryophyllene have demonstrated activity as coronavirus entry inhibitors [13]. In addition, this terpene is a selective phytocannabinoid agonist of type 2 receptors (CB2-R) [7,14,15,16] and inhibits the action of the main inflammatory mediators, such as cyclo-oxygenase 1 (COX-1), cyclo-oxygenase 2 (COX-2), inducible nitric oxide synthase (iNOS), interleukin-1β (IL-1β), interleukin-6 (IL-6), tumor necrosis factor-alpha (TNF-α), and nuclear factor kappa-light-chain-enhancer of activated B cells (NF-κB) [14,15]. The mechanism of action of caryophyllene derivatives has been studied much less well; antibacterial [17,18,19,20], cytotoxic [16,20,21], antiviral [22,23], antihyperlipidemic [20], anticholinesterase [21], antityrosinase [21], analgesic [16,24], anti-trypomastigote [25], and antitermite [26] activities have been described for a number of derivatives of this terpene. Some research shows that the diversity of biological properties of caryophyllene **1**, as well as its epoxide derivative **2**, is associated with the easy penetration of these compounds through the cell membrane [27,28]. Therefore, caryophyllene can be a convenient precursor for creating broad-spectrum drugs. Some examples of biologically active derivatives of terpene **1** are presented in Figure 1.

To enhance the biological properties of starting molecules in medicinal chemistry, the method of directed chemical transformations is widely used. Incorporation of a heterocyclic moiety into a compound’s structure provides a powerful tool for targeted tuning of solubility, lipophilicity, polarity, and hydrogen bonding ability. Heterocycles are found in the structure of a huge range of active pharmaceuticals, and many heterocyclic scaffolds can be considered privileged structures in medicinal chemistry [29].

**Scheme 1:**
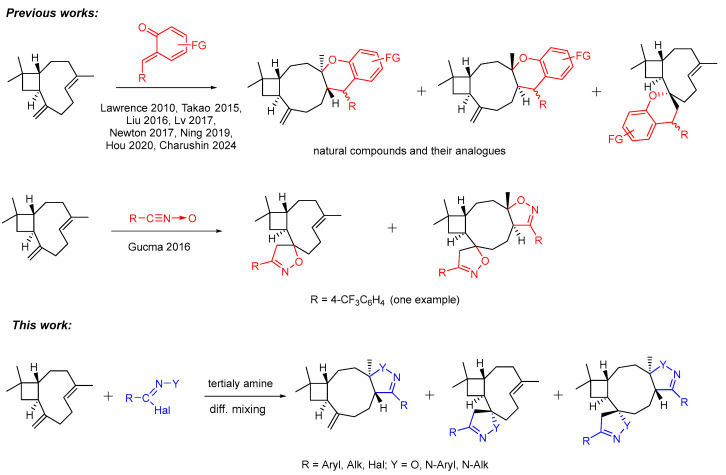
Cycloaddition reactions to (−)-β-caryophyllene described in the literature and in this work [29,30,32,33,34,35,36,37,39].

In the last decade, an actively developing direction in the chemistry of (−)-β-caryophyllene is the introduction of chromane rings into its structure through the reactions with *orto*-quinone methides [30,31,32,33,34,35,36,37], which makes it possible to obtain such natural substrates as psiguadial B [36], (+)-cytosporolide A [37], psidial A [30], guajadial [30], hyperjapones B–E [31], frutescones A, D–F [32], myrtucommulones K, N, and O [33], and rhodomentones A, B [34]. However, it may be noted that the described synthetic approach often leads to the formation of a mixture of stereoisomeric cycloaddition adducts at the endocyclic C=CH bond of terpene **1** [30,31,32,33,35,36,37] (Scheme 1) and can proceed with unsatisfactory yields [30,36]. Examples of the formation of cycloaddition adducts at the exocyclic C=CH_2_ bond of caryophyllene are much less represented in the literature without the analysis of the reaction’s chemoselectivity [32,33,34].

1,3-Dipolar cycloaddition reactions also allow the introduction of heterocyclic fragments into the target structure and are usually characterized by high regio- and stereoselectivity [38], which is an important advantage in the synthesis of drugs. To date, the only example of the addition of a 1,3-dipole to caryophyllene is described in [39] (Scheme 1) without fully assigning the stereochemistry of the resulting products.

In this work, we described the study of the nitrile oxide and nitrilimine cycloadditions to the C=C double bonds of caryophyllene. As a result of these reactions, pharmacophore fragments of isoxazoline and pyrazoline are introduced into the structure of caryophyllene, which leads to an increase in the conformational rigidity of the molecule and can be favorable for more effective interaction with biological targets. This study examined the regio-, stereo-, and chemoselectivity of the cycloaddition of nitrile oxides and nitrilimines at one or both of the C=C bonds of caryophyllene, as well as the influence of substituents in the 1,3-dipole on the reaction. For the first time, it was possible to carry out a complete stereochemical assignment of 1,3-dipolar cycloaddition adducts and establish the preferential conformation of caryophyllene in which it reacts with nitrile oxides and nitrilimines. The resulting heterocycles were tested for their cytotoxicity and for inhibiting activity against the most epidemiologically important H1N1 influenza virus. Such activity has previously been shown for nitrogen-containing derivatives of caryophyllene [22] and isocaryophyllene [23]. The nitrogen-containing derivatives of caryophyllene and isocaryophyllene investigated in these works were practically non-cytotoxic (CC_50_ > 700 µM), and some of them showed high selectivity indexes (from 100 to 7500), while others were completely inactive against the H1N1 influenza virus.

## 2. Results and Discussion

### 2.1. Synthesis

#### 2.1.1. Synthesis of Caryophyllene Derivatives Containing One Heterocyclic Fragment

**Scheme 2:**
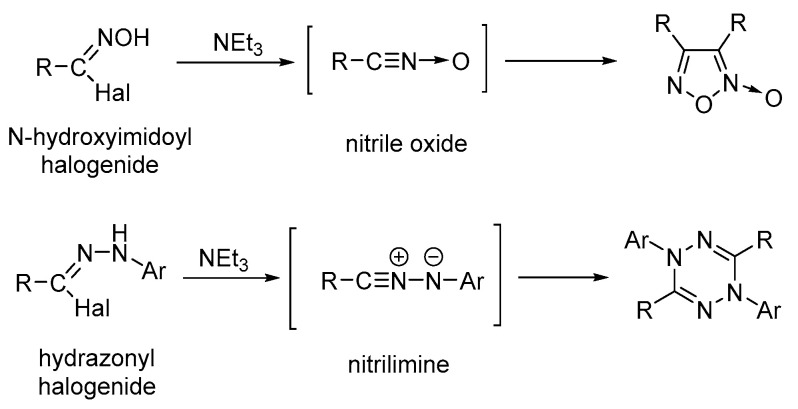
Unwanted dimerization reactions of 1,3-dipoles, complicating their addition to caryophyllene.

Several methods for carrying out the reactions of the 1,3-dipolar cycloaddition of nitrile oxides and nitrilimines to dipolarophiles are described in the literature [40]. The most convenient method is to generate these dipoles in situ from the corresponding N-hydroxyimidoyl halogenides and hydrazonyl halogenides under the action of a base (Scheme 2); such reactions occur under mild conditions, avoiding unwanted skeletal rearrangements of caryophyllene. However, the carrying out of the target cycloaddition reaction at the C=C bond of a dipolarophile can be complicated by undesirable dimerization of the initial dipoles (Scheme 2) [41,42], which can become the main reaction in the case of low-reactive dipolarophiles that do not contain strongly accepting substituents at their double bonds, which include caryophyllene.

To suppress the dimerization of nitrile oxides and nitrilimines, which can lead to a significant decrease in the yield of the target heterocycles, we used in this work the method of diffusion reagent mixing, which we had previously successfully applied to carry out 1,3-dipolar cycloaddition reactions with other substrates [40,43,44]. This technique has shown its effectiveness in the case of interaction of low-reactive dipolarophiles with rapidly dimerizing dipoles [40].

Previous techniques described in the literature for the realization of the diffusion mixing method involved the use of ex situ gas generation, which is convenient and only safe to work with in various gaseous substrates [45]. In contrast, in our proposed implementation of diffusion mixing, the reagents and formed intermediates are not gases under reaction conditions (the difference between the boiling point of the evaporating reagent and the medium can reach up to 140 °C! [40]), and the method itself is an alternative to the technique of dropping a solution of this amine into a mixture of a dipolarophile and dipole precursors **7**–**15**. In the case of diffusion mixing, amine vapors are slowly absorbed by the reaction mixture, which leads to the generation of nitrile oxides and nitrilimines in trace amounts; thus, their unwanted dimerization becomes unlikely. For comparison, in the case of dropwise addition, the amine enters the reaction mixture in portions, as a result of which many highly reactive 1,3-dipoles are formed in the local region, which, on the contrary, promotes their dimerization. The advantages of the method used include the fact that the rate of generation of nitrile oxides and nitrilimines during diffusion mixing can be controlled. When using an amine with a higher boiling point (for example, DIPEA instead of Et_3_N), it enters the reaction mixture more slowly and the highly reactive 1,3-dipole also is generated in the solution slowly, which is important for reactions with inactive dipolarophiles. The simplicity of the equipment and the ability to carry out reactions with suspensions are the advantages of the diffusion mixing technique compared to the use of flow reactors.

In order to study the influence of steric and electronic factors of nitrile oxides and nitrilimines on the course of cycloaddition reactions to caryophyllene, we synthesized various functionalized precursors, i.e., compounds **7**–**15**, of these dipoles. It was found that when carrying out 1,3-dipolar cycloaddition reactions with any of the compounds **7**–**15**, caryophyllene can add one or two equivalents of 1,3-dipoles; therefore, to increase the yields of monoadducts **16**–**24** in further reactions, we used a 5-fold excess of olefin **1**.

Reaction condition optimization was carried out on the reactions of terpene **1** interactions with compounds **10**–**12**, which are sources of rapidly dimerizing dipoles, as a result of which the yields of target heterocycles can significantly decrease. We found that when carrying out the reaction in chloroform at room temperature using the diffusion mixing method, to obtain the target heterocycles with a good overall yield, it is optimal to use triethylamine in the case of N-hydroxyimidoyl halogenides **10** and **11**, while for hydrazonyl halogenide **12,** it is better to use the higher boiling i-PrNEt_2_.

**Scheme 3:**
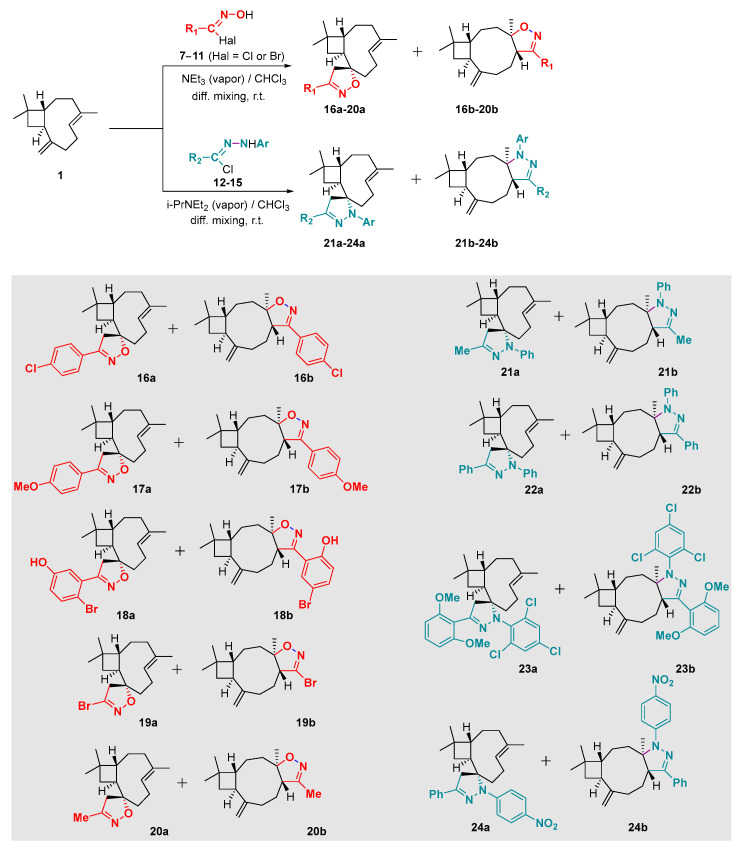
General scheme of addition of 1 equivalent of the 1,3-dipoles to (−)-β-caryophyllene and product scope.

The results of the reaction of caryophyllene with compounds **7**–**15** are presented in Scheme 3 and Table 1. It is worth noting that in all cases, the reactions proceeded with a good total yield of isomers **a** (addition product at the exocyclic C=C bond) and **b** (addition product at the endocyclic C=C bond). Monoadducts **16**–**19** and **21**–**23** were isolated as individual products by column chromatography. The low yields of heterocycles **18a,b** and **19a,b** are associated with similar chromatographic mobility of these compounds, which led to significant losses in the isolation of individual isomers. A mixture of compounds **20a** and **20b** (and also **24a** and **24b**) could not be separated.

A comparison of the chemoselectivity for the formation of isomers **a** and **b** (Table 1) demonstrates the complex nature of the dependence of the reaction results on the steric and electronic properties of the substituent in the 1,3-dipole. It is interesting that, in contrast to heterodienes [30,31,32,33,36,37], epoxidizing [45], and episulfurizing [46] agents, nitrile oxides and nitrilimines in most cases reacted with (−)-β-caryophyllene preferentially forming isomers **a**—the result of cycloaddition at the exocyclic C=CH_2_ bond that is poorly represented in the literature. The best addition chemoselectivity was demonstrated by nitrile oxides with *para*-substituted aromatic fragments (products **16a**, **17a**), nitrilimine with bulky aryl substituents (product **23a**), as well as bromonitrile oxide, which, unlike other dipoles, predominantly forms the reaction adduct at the endocyclic bond C=C **19b**.

The full assignment of the spectral lines of compounds **16a**, **16b**, **18b**, and **19b** using sets of 2D NMR spectroscopy methods (HMBC, HSQC, NOESY) allowed us to prove the structure of isomers **a** and **b** (see Supplementary Materials, Figures S8–S10, S13–S16, S25, S26, S31 and S32).

Isomers **a** and **b** can be easily distinguished by ^1^H NMR spectroscopy data. In the ^1^H NMR spectra, the characteristic proton of the CH= fragment of compounds **a** is observed in the range of 5.4–4.9 ppm, and in the region of 3.5–2.7 ppm for isomer **a**, there are two broadened doublets of the methylene group of the heterocyclic fragment. In the ^1^H NMR spectra of compounds **b**, signals of the CH_2_= group are observed in the region of 5.3–4.7 ppm, and the signal of the CH fragment of the heterocycle is observed at 3.6–2.8 ppm. It is important to note that all spiroadducts **a**, regardless of the steric properties of the substituents in the heterocycle, are characterized by the broadening of the signals of the lipophilic framework in the ^1^H and ^13^C NMR spectra, which arise due to the conformational mobility of the nine-membered ring of the caryophyllene skeleton. In the case of compounds **b**, the broadening of some signals are observed only for heterocycles with small substituents (for example, compound **21b**, see Supplementary Materials, Figures S37 and S38). Unlike isomers **a**, the introduction of bulky substituents into the structure of heterocycles **b** creates steric hindrances to conformational transitions in the molecule. Thus, the ^1^H and ^13^C NMR data of pyrazoline **23b** indicate weak conformational mobility of the nine-membered ring, as well as an apparently high barrier to rotation of the symmetrical trichlorophenyl group (see Supplementary Materials, Figures S48 and S49).

The regioselective formation of adducts **a** and **b** was established using ^13^C NMR spectroscopy data based on the characteristic signals of the quaternary carbon atoms of isoxazoline and pyrazoline fragments in the regions of 95–85 ppm and 75–70 ppm, respectively. In the case of compounds **16a**, **16b**, **18b**, **19b**, and **21b**, the structure of the products was confirmed by HMBC spectroscopy data (see Supplementary Materials, Figures S8, S12, S26, S32 and S40). It is worth noting that the regioselectivity of the addition of nitrile oxides and nitrilimines to caryophyllene does not depend on the steric properties of the substituents at positions 1 and 3 of the dipole. For example, for adducts **21**, the nitrilimine nitrogen bearing the bulky aryl group adds to the most substituted carbon atom of the caryophyllene C=C bond.

**Scheme 4:**
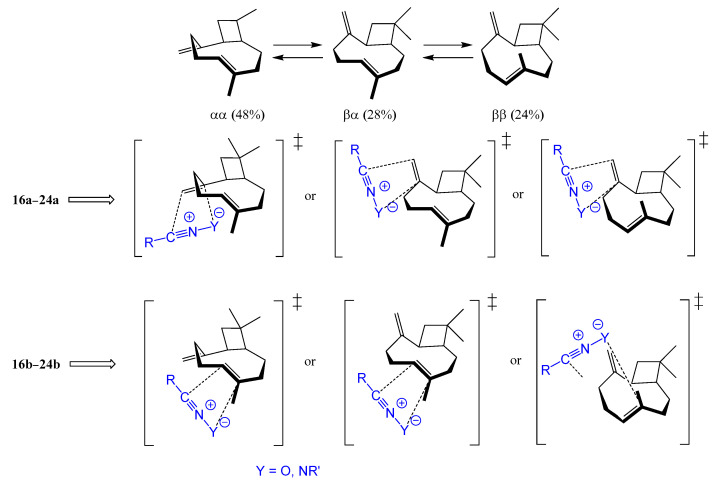
Possible modes of 1,3-dipole addition to various conformations of caryophyllene, leading to the formation of products **16**–**24**.

An important advantage of the cycloaddition reaction of nitrile oxides and nitrilimines to caryophyllene was the stereoselective formation of heterocycles **16**–**24** without some minor isomers. For comparison, hetero-Diels–Alder reactions described in the literature often proceed with the formation of an indivisible mixture of stereoisomers [30,31,32,33,36,37], which is explained by the interaction of heterodienes with the three main conformers of caryophyllene: αα, βα, and ββ (Scheme 4, the ratios of conformers data are taken from [47]). The stereoselective interaction of nitrile oxides and nitrilimines with terpene **1** may be associated with the very high sensitivity of these dipolariphiles to the steric properties of the dipolarophile, as a result of which, for caryophyllene conformation, the 1,3-dipole can approach caryophyllene in the transition state only from one of the two possible sides of the plane of the C=C bond, as shown in Scheme 4.

**Figure 2:**
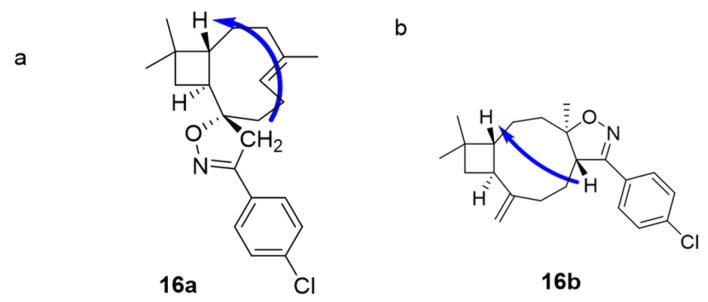
(**a**) Principal correlation observed in ^1^H-^1^H NOESY-1D NMR spectrum of the compound **16a** (see Supplementary Materials, Figure S10). (**b**) Characteristic correlation observed in GEMSTONE NOESY NMR spectrum of the compound **16b** (see Supplementary Materials, Figure S16).

The configuration of spiro-heterocycles **16**–**24** was determined on the examples of compounds **16a** and **16b** using ^1^H-^1^H NOESY-1D and GEMSTONE NOESY NMR spectroscopy: upon irradiation of the corresponding CH_2_ protons and CH groups of isoxazoline heterocycles (see Supplementary Materials, Figures S10C and S16E), their interaction with one of the protons of the cyclobutane fragment (Figure 2a,b) was observed.

**Figure 3:**
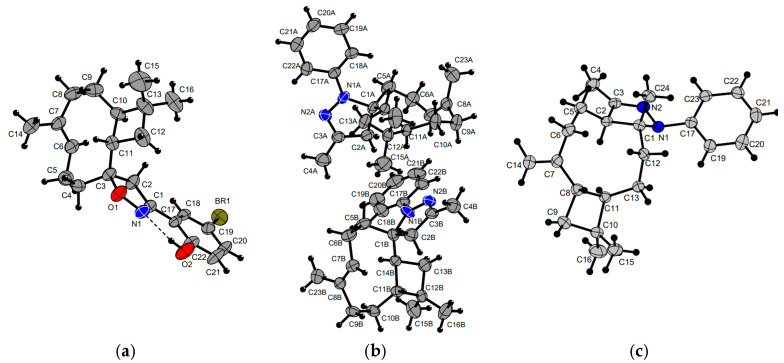
Molecular structures of compound **18a** (**a**), compound **21a** (**b**), and compound **21b** (**c**). Displacement ellipsoids are drawn at 30% probability level.

The structures of compounds **18a**, **21a**, and **21b** were confirmed by the X-ray diffraction data. Figure 3a–c show the atom numeration in the molecules **18a**, **21a**, and **21b**, respectively.

The hydroxy group of the compound **18a** participates in the formation of a hydrogen bond with the nitrogen atom N1 (see Figure 3a and Table S2 in Supplementary Materials). Hydrogen bonding stabilizes the planar arrangement of five- and six-membered rings relative to each other. Two independent molecules whose structures completely coincide are located in the unit cell of crystal structure **21a**. The torsion angle, which determines the relative position of the five- and six-membered cycles, is about 3–4 degrees. That is, their arrangement is almost flat. The stabilization of the flat structure is achieved by the interaction of the free electron pair of the nitrogen atom N2A and the hydrogen atom H22A at the carbon atom of the phenyl group (N2B and H22B for the second molecule, respectively). π–π conjugation between the cycles also determines their location. In the case of isomer **21b**, the N1 atom has a pyramidal configuration.

Despite the fact that, according to X-ray diffraction data, compounds **18a**, **21a**, and **21b** in the crystalline state have a βα-conformation of the caryophyllene skeleton, the characteristic broadening of the signals of protons of the lipophilic skeleton in the ^1^H and ^13^C NMR spectra indicates the conformational mobility of the nine-membered rings of these heterocycles in solutions. Apparently, the caryophyllene skeleton of adducts **a** can exist in all three forms (see Scheme 4), while for adducts **b**, the transition to the ββ conformation is apparently impossible due to steric hindrances created by the heterocyclic fragment.

The data obtained on the configuration of heterocycles correspond with the literature data for the products of the hetero-Diels–Alder reaction at the C=CH_2_ bond of caryophyllene [32,33,34]. At the same time, the configuration of adducts **b** established by ^1^H-^1^H NOESY NMR spectroscopy and X-ray diffraction methods contradicts the only example of isoxazoline from [39], in which the authors assumed the structure of the adduct based on COSY, NOESY, and HMBC NMR spectroscopy data. It is worth noting that our proposed spatial arrangement of substituents in the caryophyllene skeleton of compounds **b** coincides with the configuration of the known products of epoxidation [45] and episulfurization [46] of terpene **1**, as well as the products of its interaction with heterodienes [30,31,32,33,35,36,48].

#### 2.1.2. Synthesis of Caryophyllene Derivatives with Two Heterocyclic Fragments

**Scheme 5:**
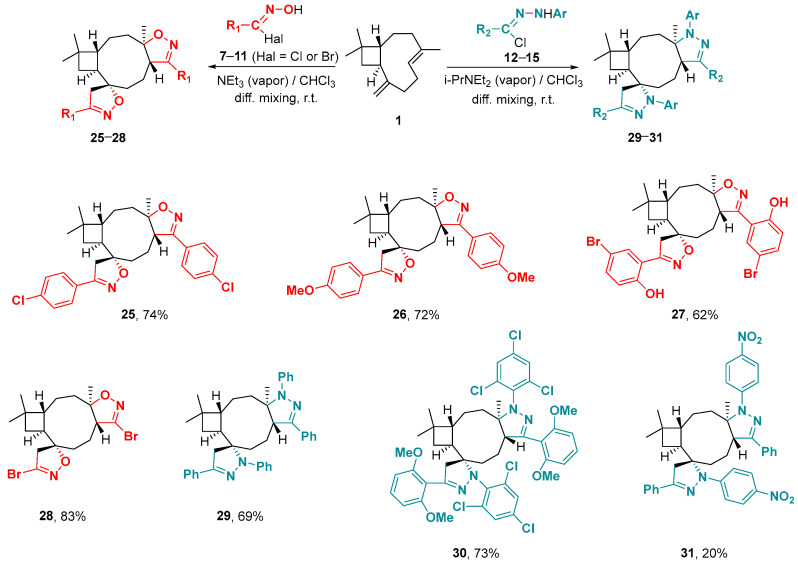
General scheme of addition of 2 equivalent of the 1,3-dipoles to (−)-β-caryophyllene and product scope.

Using conditions optimized for the synthesis of monoadducts **a** and **b**, we further carried out caryophyllene **1** reactions with a 2–3-fold excess of the precursors of nitrile oxide **7**–**11** and nitrilimines **12**–**15**. With the exception of compound **31**, in all cases, the target cycloaddition diadducts were obtained in good yields (Scheme 5). The low yield of product **31** may be a result of the lower activity of the nitrilimine intermediate obtained during its formation due to the stabilization of the negative charge in the 1,3-dipole by the *para*-nitrophenyl substituent.

The presence of two isoxazoline or pyrazoline fragments in products **25**–**31** is confirmed by the data of ^1^H NMR spectra of these compounds in the region of 3.7–2.7 ppm: we observed in this area two doublets of doublets and a singlet of CH_2_ and CH groups of these heterocycles. In the case of pyrazoline **30**, ^13^C NMR spectroscopy data indicate a high barrier to rotation of two halogenated aromatic moieties and one dimethoxy-substituted ring. The structure of the synthesized diadducts **25**–**31** was confirmed on the examples of compounds **27** and **31** by HSQC, HMBC, and ^1^H NOESY NMR spectroscopy (see Supplementary Materials, Figures S58–S60, S71 and S72).

Also, similar to monoadducts **16**–**24**, broadened signals of the lipophilic framework were observed in the ^1^H and ^13^C NMR spectra of heterocycles **25**, **26**, and **28**–**31**, which indicate the conformational mobility of the caryophyllene skeleton of these compounds. On the example of compound **31**, it was shown that when the temperature increases above room temperature, a narrowing of the signals in the NMR spectra is observed (see Supplementary Materials, Figure S69).

### 2.2. Biological Testing

#### 2.2.1. Antiviral Activity

Some compounds synthesized in this study have been tested for their potential inhibitory activity against influenza viruses of the H1N1 subtype A/Puerto Rico/8/34. In the course of experiments, the values of 50% cytotoxic concentration (CC_50_), 50% inhibiting concentration (IC_50_), and selectivity index (CC_50_/IC_50_ ratio) have been estimated for each compound. The resulting compounds were found to be virtually insoluble in water, but soluble in DMSO. Therefore, for biological testing, the stock solutions of each compound were prepared in DMSO following by the preparation of serial dilutions in the cell culture medium (MEM). The results are summarized in Table 2.

Among the compounds we obtained, the highest values of activity and selectivity were shown by monoadducts of cycloaddition to the caryophyllene exocyclic C=CH_2_**a**, the synthesis of which is poorly described in the literature. In the case of isomers **b**, lower activity and selectivity were found relative to their structural analogues **a**. A significant effect of substituents in heterocycles **16**–**19** and **21**–**23** on the biological activity of monoadducts was also discovered. Thus, halogenphenyl-substituted isoxazoline **16a** had the most optimal biological profile. With the exception of compounds **16a**, **21b**, and **23b**, the remaining monoadducts showed significant cytotoxicity in the MDCK cell line tested. A comparison of adduct **16a** with the reference compounds Rimantadine, Amantadine, Deitiforin, and Ribavirin shows that this isoxazoline has higher antiviral activity but is inferior in selectivity to the drug Ribavirin. Among the caryophyllene derivatives containing two heterocycles, the best data on antiviral activity were shown by substrate **25**, which is close in these indicators to the reference compounds Rimantadine and Amantadine. It is worth noting that with the exception of substrate **28**, the IC_50_ values of the remaining diadducts were significantly lower than those of the analogous monoadducts.

**Figure 4:**
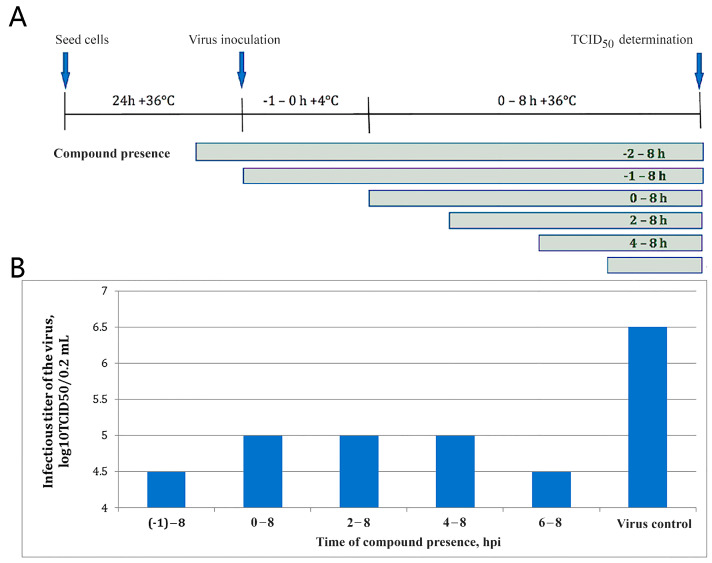
Scheme (**A**) and results (**B**) of time-of-addition assay for virus-inhibiting activity of **25** against influenza virus A/Puerto Rico/8/34 (H1N1) in MDCK cells.

**Figure 5:**
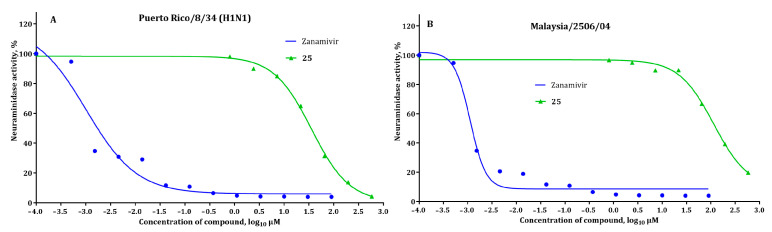
Anti-NA activity of (−)-β-caryophyllene derivatives against influenza viruses A/Puerto Rico/8/34 (H1N1) (**A**) and B/Malaysia/2506/04 (**B**).

In order to estimate the potential target for the active compound, a time-of-addition experiment has been conducted. The compound was added to the infected cells at various time points considering the moment of virus infection as point zero (Figure 4). As one can see from the results obtained, compound **25** has demonstrated its virus-inhibiting activity up to the very late stages of virus infection (6 hpi). This suggests that **25** is directed to the viral or cellular proteins necessary at the late stages of the viral cycle. Viral neuraminidase (NA) is an example of such a protein due to its function of releasing progeny virions from the cell surface. To assess whether **25** is able to inhibit NA directly, we performed a cell-free anti-NA assay using a synthetic luminescent substrate for NA. We found (Figure 5) that indeed, it possesses slight NA-suppressing activity, although of a much lower level than that of the reference compound zanamivir. Importantly, **25** has demonstrated slight NA inhibition regarding influenza A and B viruses, its IC_50_s being 35.8 and 115.1 μM, correspondingly. These parameters for zanamivir were 1.0 and 1.1 nM, correspondingly.

#### 2.2.2. Cytotoxicity

Due to the fact that many synthesized heterocyclic caryophyllene derivatives showed a relatively high level of cytotoxicity on the MDCK cells (see Table 2), to study the structure–activity relationship, synthesized isoxazolines and pyrazolines were investigated in the MTT test on human cancer cell lines DU145, A549, human healthy cells VA-13, HEK293, and murine mammary cell line EMT6. The results presented in Table 3 demonstrate that monoadducts **a** have typically greater cytotoxicity, while isomers **b** and diadducts **25**, **26**, and **28**–**30** are significantly less cytotoxic.

Isoxazoline **17a** and pyrazoline **21a** were shown to be extremely toxic, killing all cells within 48 h, whereas **23a** was only found to be extremely toxic against EMT6 (Table 2). Compounds **17b** and **23a** demonstrated toxicity on all cells after 72 h, with IC_50_ < 50 µM. Compounds **22a**, **28**, **22b**, and **18b** showed little toxicity and IC_50_ values ranging from 50 to 100 µM on normal cells HEK293 and VA-13. Diadduct **25** is harmful to both D145 and A549.

Additional cytotoxicity studies of compounds **17a**, **21a**, **23a**, and **25** were carried out with the MTT test using a panel of human cancer cell lines HCT116, HT-29, MCF7, SKBR3, SK-MEL-28, A549, DU145, and human normal cells (HEK-293). With an IC_50_ range of 12.3 to 39.4 µM, pyrazoline **21a** showed the most promising anticancer activity on a variety of cancer cell lines. Only compounds **17a** and **21a** exhibited cytotoxic effects against SKBR3 in breast cancer cells, with IC_50_ values of 36.5 ± 3.2 µM and 12.3 ± 1.0 µM, respectively, whereas MCF7 did not show any sensitivity to any of the studied caryophyllene derivatives (Table 3). Compounds **25** and **23a** did not show any cytotoxic effects on HCT116 and SK-MEL-28; however, spiroheterocycle **21a** showed high cytotoxic effects with IC_50_ = 22.4 ± 0.8 µM and 24.9 ± 1.3 µM for HCT116 and SK-MEL-28, respectively (Table 4 and Table S5).

In general, one can note the low selectivity of heterocyclic caryophyllene derivatives towards healthy and tumor cells.

## 3. Materials and Methods

### 3.1. Reagents

Starting (−)-β-caryophyllene **1** is commercially available and was used without further purification. Experimental details for the preparation of hydroxymoyl halides **7**, **8**, **10**, and **11** and imidoyl chlorides **12**, **13**, and **15** has been previously described [49,50,51,52,53]. The synthesis of 1,3-dipole precursors **9** and **14** is described in Section 3.4. Sigma-Aldrich (Schnelldorf, Germany) provided 3-(4,5-dimethylthiazol-2-yl)-2,5-diphenyltetrazolium bromide (MTT). Trypan blue, phosphate-buffered saline (PBS), and dimethyl sulfoxide (DMSO) were purchased from PanEco (Moscow, Russia). Fetal bovine serum was obtained from HyClone (Logan, UT, USA), along with flasks and plates purchased from Nunc (Moscow, Russia).

### 3.2. Equipment

^1^H and ^13^C NMR spectra (Figures S1–S76) were recorded on a Bruker Avance instrument with an operating frequency of 400 MHz for ^1^H NMR and 101 MHz for ^13^C NMR. Chemical shifts are given in parts per million on a scale of δ relative to hexamethyldisiloxane as an internal standard. The 2D NMR was measured using an Agilent 400 spectrometer operating at 400 MHz for ^1^H and 100.6 MHz for ^13^C. NMR spectra were processed and analyzed using the Mnova software (Mestrelab Research, Santiago, Spain; https://mestrelab-store.myshopify.com/products/mnova-nmr-perpetual-academic-single-license, accessed on 20 January 2024).

High-resolution mass spectra were recorded on an Orbitrap Elite mass spectrometer (Thermo Scientific, Waltham, MA, USA) with IREP. To enter solutions with a concentration of 0.1–9 μg/mL (in 1% formic acid in acetonitrile), direct injection into the ion source using a syringe pump (5 μL/min) was used. Spray voltage ± 3.5 kV; capillary temperature 275 °C.

For compounds **18a**, **21a**, and **21b**, the X-ray data were collected by using a STOE diffractometer Pilatus100K detector, with focusing mirror collimation Cu Kα (1.54086 Å) radiation and rotation method mode. The STOE X-AREA software (https://www.stoe.com/products/xarea/, accessed on 20 January 2024) was used for cell refinement and data reduction. Data collection and image processing was performed with X-Area 1.67 (STOE & Cie GmbH, Darmstadt, Germany, 2013). Intensity data were scaled with LANA (part of X-Area) in order to minimize differences of intensities of symmetry-equivalent reflections (multi-scan method). The structures were solved and refined with the SHELXT [54] program. The non-hydrogen atoms were refined by using the anisotropic full matrix least-square procedure. All hydrogen atoms were placed in the calculated positions and allowed to ride on their parent atoms [C-H 0.93–0.97; Uiso 1.2 Ueq (parent atom)]. Molecular geometry calculations were performed with the SHELX program, and the molecular graphics were prepared by using the DIAMOND [55] software, version 5.0. Some crystallographic data are listed in Tables S1, S3 and S4 (see the Supplementary Materials part of this article). The geometric parameters of the hydrogen bond for molecule **18a** are given in Table S2.

CCDC-2351070, CCDC-2351071, and CCDC-2351108 contain the supplementary crystallographic and geometric data for **18a**, **21b**, and **21a**, respectively. These data can be obtained free of charge from The Cambridge Crystallographic Data Centre via www.ccdc.cam.ac.uk/data_request/cif (accessed on 9 August 2024).

### 3.3. Cell Lines and Cytotoxicity Evaluation

Colon cancer (HCT116, HT29), breast cancer (MCF7, SKBR3), melanoma (SK-MEL28), lung cancer (A549), prostate cancer (DU145), human normal cells (VA-13 and HEK293), and murine mammary cell lines (EMT6) were purchased from ATCC (Manassas, VA, USA). Cells were routinely grown in RPMI 1640 culture medium, supplemented with 10% fetal bovine serum, glutamine, and 100 U/mL penicillin. HCT116, HT29, MCF7, SKBR3, SK-MEL28, A549, DU145, VA-13, HEK293, and EMT6 cells were grown in flasks in RPMI 1640 fresh culture medium with supplements at 37 °C and 5% CO_2_. Cells were grown as monolayer cultures, and the cells in the exponential growth phase were trypsinized and suspended in the supplemented RPMI 1640 medium.

### 3.4. Preparation of Compounds **9, 14, and 16–35**

#### 3.4.1. Synthesis of Benzaldehyde Oxime **34**

*5-Bromo-2-hydroxybenzaldehyde oxime* (**34**). A suspension of 995 mg (4.95 mmol, 1 eq.) of 5-bromo-2-hydroxybenzaldehyde and 378 mg (5.44 mmol, 1.1 eq.) of hydroxylamine hydrochloride in ethanol (12 mL) and water (24 mL) was cooled at 5 °C with an ice bath. Then a 1.15 mL 32% aqueous solution of sodium hydroxide (12.40 mmol, 2.2 eq.) was added dropwise within a 10 min period, whereupon most of the solid dissolved. After 1 h stirring at room temperature, the resulting mixture was then acidified with HCl (5 N). The mixture was then extracted with diethyl ether (3 × 30 mL). The organic phase was dried over anhydrous Na_2_SO_4_ and filtered; the solvent was removed under reduced pressure. Compound **34** (1.059 g, 99%) was obtained as a light yellow solid and used for the next reaction.

**^1^H NMR** (400 MHz, DMSO-d6): δ 11.47 (bs, 1H), 10.30 (s, 1H), 8.26 (s, 1H), 7.63 (d, *J* = 2.4 Hz, 1H), 7.34 (dd, *J* = 8.7, 2.4 Hz, 1H), 6.84 (d, *J* = 8.7 Hz, 1H). **^13^C NMR** (101 MHz, DMSOd6): δ 155.1, 145.6, 132.8, 129.2, 120.8, 118.4, 110.5. **HRMS** (ESI+) *m*/*z* calcd. for (C_7_H_7_BrNO_2_, M + H): 215.9655, found: (M + H): 215.9657.

#### 3.4.2. Synthesis of N-Hydroxyimidoyl Halogenide **9**

*5-Bromo-N,2-dihydroxybenzimidoyl chloride* (**9**). To a solution of 540 mg (2.50 mmol, 1 eq.) benzaldehyde oxime **34** in DMF (5 mL) at 5 °C, 367 mg (2.75 mmol, 1.1 eq.) *N*-chlorosuccinimide was added portion-wise over 30 min. After the addition was complete, the reaction mixture was stirred overnight at room temperature. The reaction mixture was diluted with 50 mL of water and extracted with ether (2 × 30 mL). The organic phase was washed with water (2 × 30 mL), dried over anhydrous Na_2_SO_4_, and filtered; the solvent was removed under reduced pressure. Compound **9** (557 mg, 89%) was obtained as a light yellow solid.

**^1^H NMR** (400 MHz, DMSO-d6): δ 12.45 (s, 1H), 10.39 (bs, 1H), 7.52 (d, *J* = 2.5 Hz, 1H), 7.46 (dd, *J* = 8.7, 2.5 Hz, 1H), 6.91 (d, *J* = 8.7 Hz, 1H). **^13^C NMR** (101 MHz, DMSOd6): δ 155.1, 134.0, 133.4, 131.8, 121.6, 118.7, 109.8. **HRMS** (ESI−) *m*/*z* calcd. for (C_7_H_4_BrClNO_2_, M − H): 247.9119, found: (M − H): 247.9108.

#### 3.4.3. Synthesis of Benzoyl Phenylhydrazine **35**

*2,6-Dimethoxy-N’-(2,4,6-trichlorophenyl)benzohydrazide* (**35**). A suspension of 1.00 g (5.49 mmol, 1 eq.) 2,6-dimethoxybenzoic acid in SOCl_2_ (10 mL) was refluxed for 2 h, cooled, and then distilled to remove excess SOCl_2_. A solution of 1.28 g (6.04 mmol, 1.1 eq.) 2,4,6-trichlorophenylhydrazine was dissolved in 0.97 mL (12.09 mmol, 2.2 eq.) pyridine and cooled at 5 °C with an ice bath. In parallel, 2,6-dimethoxybenzoyl chloride was dissolved in 5 mL of THF. The 2,6-dimethoxybenzoyl chloride solution was then added dropwise at 5 °C to the 2,4,6-trichlorophenylhydrazine solution. After addition, the temperature was allowed to warm to room temperature and the medium was stirred for 2 h. After completion of the reaction, water (30 mL) was added and the precipitate was filtered, washed by diethyl ether (3 × 10 mL), and dried. Compound **35** (1.38 g, 67%) was obtained as a white solid and used for the next reaction.

**^1^H NMR** (400 MHz, DMSO-d6): δ 10.20 (d, *J* = 3.1 Hz, 1H), 7.54 (s, 2H), 7.35–7.27 (m, 1H), 7.11 (d, *J* = 3.1 Hz, 1H), 6.65 (d, *J* = 8.4 Hz, 2H), 3.70 (s, 6H). **^13^C NMR** (101 MHz, DMSOd6): δ 164.5, 157.2 (2C), 141.3, 130.7, 128.2 (2C), 126.0 (2C), 125.9, 114.0, 104.0 (2C), 55.6 (2C). **HRMS** (ESI+) *m*/*z* calcd. for (C_15_H_14_Cl_3_N_2_O_3_, M + H): 375.0065, found: (M + H): 375.0067.

#### 3.4.4. Synthesis of Hydrazonyl Halogenide **14**

*2,6-Dimethoxy-N-(2,4,6-trichlorophenyl)benzohydrazonoyl chloride* (**14**). To a suspension of 500 mg (1.33 mmol, 1 eq.) benzoyl phenylhydrazine **35** in 3 mL anhydrous acetonitrile under a flow of nitrogen, 436 mg (1.66 mmol, 1.25 eq.) triphenylphosphine and 161 μL (1.66 mmol, 1.25 eq.) anhydrous carbon tetrachloride were added and left to react overnight at room temperature. After the completion of the reaction, the solvent was evaporated under reduced pressure and the crude product was purified by column chromatography using chloroform as the eluent. Compound **14** (195 mg, 37%) was obtained as a light yellow solid.

**^1^H NMR** (400 MHz, CDCl_3_): δ 8.07 (bs, 1H), 7.36–7.28 (m, 3H), 6.57 (d, *J* = 8.4 Hz, 2H), 3.84 (s, 6H). **^13^C NMR** (101 MHz, CDCl_3_): δ 158.3 (2C), 136.1, 131.2 (2C), 128.3 (2C), 127.9, 126.7, 121.8, 113.4, 103.5 (2C), 55.6 (2C). (The elemental composition of compound **14** could not be confirmed by HRMS, which may be due to the rapid decomposition of this compound under ionization conditions. However, the use of **14** in 1,3 dipolar addition reactions led to the expected products **23a**, **23b**, and **30**, the structures of which were confirmed by NMR spectroscopy and HRMS).

#### 3.4.5. General Procedure for Synthesis of Compounds **16**–**24**

A mixture of 1.60 mmol of (−)-β-caryophyllene **1** (5 eq.) and 0.32 mmol N-hydroxyimidoyl halide or imidoyl chloride (1 eq.) in 3 mL of chloroform was placed into a 15 mL vial (diameter 1.3 cm). This vial was then placed in closed 50 mL vial (diameter 3.5 cm) containing amine (35.85 mmol, ~5 mL, triethylamine for hydroxymoyl halide or DIPEA for imidoyl chloride) and the reaction mixture was stirred at room temperature for 2–4 days (TLC or NMR control). After the completion of the reaction (monitored by TLC and NMR control), the mixture from the inner vial was diluted with 10 mL of chloroform, transferred to a separating funnel, and washed with 2% aqueous HCl (2 × 10 mL). The organic phase was dried over anhydrous Na_2_SO_4_, the solvent was removed under reduced pressure, and the residue was purified by column chromatography on silica gel using chloroform as an eluent.

*(1S,2R,9R,E)-3′-(4-Chlorophenyl)-6,10,10-trimethyl-4′H-spiro[bicyclo [7.2.0]undecane-2,5′-isoxazol]-5-ene* (**16a**) and *(3aR,6aS,8aR,10aS)-3-(4-chlorophenyl)-8,8,10a-trimethyl-6-methylene-3a,5,6,6a,7,8,8a,9,10,10a-decahydro-4H-cyclobuta* [5,6]*cyclonona [1,2-d]isoxazole* (**16b**). From 327 mg (1.60 mmol) (−)-β-caryophyllene **1** and 61 mg (0.32 mmol) hydroximoyl chloride **7**, compound **16a** (65 mg, 56%) was obtained as a white solid and compound **16b** (32 mg, 29%) was obtained as a pale yellow oil.

Major isomer **16a: ^1^H NMR** (400 MHz, CDCl_3_): δ 7.67–7.63 (2H, m), 7.41–7.36 (2H, m), 5.20 (1H, bs), 3.28 (bd, *J* = 16.5 Hz, 1H), 3.15 (bd, *J* = 16.5 Hz, 1H), 2.44–2.27 (m, 2H), 2.13–1.90 (m, 5H), 1.81 (dd, *J* = 12.1, 8.8 Hz, 1H), 1.70 (s, 3H), 1.63–1.52 (m, 3H), 1.34–1.25 (m, 1H), 0.96 (s, 3H), 0.92 (s, 3H). **^13^C NMR** (101 MHz, CDCl_3_): 154.3, 137.8, 135.6, 129.0 (3C), 127.8 (2C), 120.5, 94.7, 49.7, 48.6, 40.2, 38.8, 36.9, 36.4, 32.1, 30.2, 30.0, 23.6, 21.7, 16.1. **HRMS** (ESI+) *m*/*z* calcd. for (C_22_H_29_ClNO, M + H): 358.1932, found: (M + H): 358.1938.

Minor isomer **16b: ^1^HNMR** (400 MHz, CDCl_3_): δ 7.52–7.46 (2H, m), 7.38–7.32 (2H, m), 5.20–5.19 (m, 1H), 5.02–5.01 (m, 1H), 3.47–3.41 (m, 1H), 2.50 (dt, ^2^*J* = 13.4, ^3^*J* = 4.6 Hz, 1H), 2.46–2.36 (m, 1H), 1.96–1.88 (m, 2H), 1.83 (dd, ^2^*J* = 10.6, ^3^*J* = 10.6 Hz, 1H), 1.78–1.57 (m, 7H), 1.48 (s, 3H), 1.02 (s, 6H). **^13^C NMR** (101 MHz, CDCl_3_): δ 159.3, 152.3, 135.4, 129.0 (2C), 128.6, 128.5 (2C), 111.0, 90.2, 57.0, 48.9, 47.7, 42.0, 36.8, 34.9, 33.9, 30.0, 27.6, 26.3, 21.8, 19.4. **HRMS** (ESI+) *m*/*z* calcd. for (C_22_H_29_ClNO, M + H): 358.1932, found: (M + H): 358.1939.

*(1S,2R,9R,E)-3′-(4-Methoxyphenyl)-6,10,10-trimethyl-4′H-spiro[bicyclo [7.2.0]undecane-2,5′-isoxazol]-5-ene* (**17a**) and *(3aR,6aS,8aR,10aS)-3-(4-methoxyphenyl)-8,8,10a-trimethyl-6-methylene-3a,5,6,6a,7,8,8a,9,10,10a-decahydro-4H-cyclobuta* [5,6]*cyclonona [1,2-d]isoxazole* (**17b**). From 327 mg (1.60 mmol) (−)-β-caryophyllene **1** and 59 mg (0.32 mmol) hydroximoyl chloride **8**, compound **17a** (52 mg, 46%) was obtained as a white solid and compound **17b** (29 mg, 25%) was obtained as a pale yellow oil.

Major isomer **17a: ^1^H NMR** (400 MHz, CDCl_3_): δ 7.69–7.64 (2H, m), 6.96–6.92 (2H, m), 5.23 (1H, bs), 3.85 (s, 3H), 3.30 (bd, *J* = 16.3 Hz, 1H), 3.17 (bd, *J* = 16.3 Hz, 1H), 2.40–2.29 (m, 2H), 2.12–1.90 (m, 5H), 1.81 (dd, *J* = 12.0, 8.7 Hz, 1H), 1.70 (s, 3H), 1.57 (bs, 3H), 1.40–1.29 (m, 1H), 0.96 (s, 3H), 0.92 (s, 3H). **^13^C NMR** (101 MHz, CDCl_3_): δ 160.3, 154.3, 137.1, 127.5 (2C), 122.6, 120.1, 113.7 (2C), 93.2, 55.0, 49.2, 48.1, 39.7, 38.3, 36.2, 31.5, 29.5, 23.2, 21.3, 15.6. **HRMS** (ESI+) *m*/*z* calcd. for (C_23_H_32_NO_2_, M + H): 354.2428, found: (M + H): 354.2432.

Minor isomer **17b: ^1^HNMR** (400 MHz, CDCl_3_): δ 7.52–7.48 (2H, m), 6.92–6.89 (2H, m), 5.20–5.19 (m, 1H), 5.03–5.02 (m, 1H), 3.83 (s, 3H), 3.44 (dd, *J* = 10.4, 3.6 Hz, 1H), 2.53–2.45 (m, 1H), 2.44–2.37 (m, 1H), 1.98–1.87 (m, 2H), 1.83 (t, *J* = 10.6 Hz, 1H), 1.81–1.73 (m, 3H), 1.72–1.61 (m, 4H), 1.47 (s, 3H), 1.02 (s, 6H). **^13^C NMR** (101 MHz, CDCl_3_): δ 160.6, 159.8, 152.3, 128.7 (2C), 122.5, 114.1 (2C), 111.0, 89.4, 56.9, 55.4, 49.2, 47.7, 42.0, 36.7, 34.9, 33.8, 30.0, 27.7, 26.3, 21.8, 19.4. **HRMS** (ESI+) *m*/*z* calcd. for (C_23_H_32_NO_2_, M + H): 354.2428, found: (M + H): 354.2433.

*4-Bromo-2-((1S,2R,9R,E)-6,10,10-trimethyl-4′H-spiro[bicyclo [7.2.0]undecane-2,5′-isoxazol]-5-en-3′-yl)phenol* (**18a**) and *4-bromo-2-((3aS,6aS,8aR,10aR)-8,8,10a-trimethyl-6-methylene-3a,5,6,6a,7,8,8a,9,10,10a-decahydro-4H-cyclobuta* [5,6]*cyclonona [1,2-d]isoxazol-3-yl)phenol* (**18b**). From 327 mg (1.60 mmol) (−)-β-caryophyllene **1** and 80 mg (0.32 mmol) hydroximoyl chloride **9**, compound **18a** (45 mg, 34%) and compound **18b** (21 mg, 16%) were obtained as white solids.

Major isomer **18a: ^1^H NMR** (400 MHz, CDCl_3_): δ 9.96 (s, 1H), 7.41–7.36 (2H, m), 6.95–6.90 (1H, m), 5.22 (1H, bs), 3.36 (bd, *J* = 16.5 Hz, 1H), 3.24 (bd, *J* = 16.5 Hz, 1H), 2.47–2.30 (m, 2H), 2.16–2.05 (m, 2H), 2.03–1.98 (m, 3H), 1.70 (dd, *J* = 12.0, 8.7 Hz, 1H), 1.71 (s, 3H), 1.64–1.51 (bs, 3H), 1.30–1.21 (m, 1H), 0.97 (s, 3H), 0.94 (s, 3H). **^13^C NMR** (101 MHz, CDCl_3_): δ 156.4, 138.0, 133.8 (2C), 130.3, 120.2, 118.9, 116.6, 111.1, 94.1, 49.5, 48.8, 40.1, 38.6, 36.8, 36.5, 32.3, 30.1, 29.9, 23.6, 21.6, 16.1. **HRMS** (ESI−) *m*/*z* calcd. for (C_22_H_27_BrNO_2_, M − H): 416.1220, found: (M + H): 416.1230.

Minor isomer **18b: ^1^H NMR** (400 MHz, CDCl_3_): δ 9.92 (bs, 1H), 7.35–7.32 (m, 2H), 6.92–6.88 (m, 1H), 5.32–5.31 (m, 1H), 5.20–5.19 (m, 1H), 3.48 (bd, *J* = 11.6 Hz, 1H), 2.67–2.57 (m, 1H), 2.46–2.36 (m, 1H), 1.96–1.80 (m, 5H), 1.73–1.62 (m, 4H), 1.56 (s, 3H), 1.55–1.45 (m, 1H), 1.03 (s, 6H). **^13^C NMR** (101 MHz, CDCl_3_): δ 160.4, 156.9, 151.8, 133.5, 130.7, 119.1, 115.8, 111.8, 111.1, 89.5, 56.7, 49.1, 48.6, 41.5, 36.8, 35.0, 33.8, 29.8, 27.8, 26.7, 21.7, 18.9. **HRMS** (ESI+) *m*/*z* calcd. for (C_22_H_29_BrNO_2_, M + H): 416.1220, found: (M + H): 416.1230.

*(1S,2R,9R,E)-3′-Bromo-6,10,10-trimethyl-4′H-spiro[bicyclo [7.2.0]undecane-2,5′-isoxazol]-5-ene* (**19a**) and *(3aR,6aS,8aR,10aR)-3-bromo-8,8,10a-trimethyl-6-methylene-3a,5,6,6a,7,8,8a,9,10,10a-decahydro-4H-cyclobuta* [5,6]*cyclonona [1,2-d]isoxazole* (**19b**). From 327 mg (1.60 mmol) (−)-β-caryophyllene **1** and 65 mg (0.32 mmol) hydroximoyl bromide **10**, compound **19b** (11 mg, 11%) and compound **19a** (9 mg, 9%) were obtained as white solids.

Major isomer **19b: ^1^H NMR** (400 MHz, CDCl_3_): δ 5.10–5.09 (m, 1H), 4.99–4.98 (m, 1H), 3.10 (t, *J* = 6.2 Hz, 1H), 2.50 (ddd, *J* =14.0, 9.0, 5.1 Hz, 1H), 2.41–2.33 (m, 1H), 2.11–2.02 (m, 2H), 1.84–1.56 (m, 6H), 1.55–1.51 (m, 1H), 1.36–1.28 (m, 1H), 1.28 (s, 3H), 1.01 (s, 3H), 1.00 (s, 3H). **^13^C NMR** (101 MHz, CDCl_3_): δ 150.8, 144.1, 112.5, 90.9, 58.1, 52.9, 43.8, 41.3, 37.1, 35.5, 34.3, 29.9, 24.7, 24.5, 22.0, 19.6. **HRMS** (ESI+) *m*/*z* calcd. for (C_16_H_25_BrNO, M + H): 326.1114, found: (M + H): 326.1115.

Minor isomer **19a: ^1^H NMR** (400 MHz, CDCl_3_): δ 5.07 (1H, bs), 3.27–3.17 (m, 1H), 3.11–2.99 (m, 1H), 2.47–2.20 (m, 2H), 2.18–1.84 (m, 5H), 1.81 (dd, *J* = 11.9, 8.7 Hz, 1H), 1.66 (s, 3H), 1.59–1.47 (m, 3H), 1.33–1.24 (m, 1H), 0.96 (s, 6H). **^13^C NMR** (101 MHz, CDCl_3_) (some carbon signals are not observed due to conformational mobility): δ 120.1, 112.3, 95.5, 85.0, 49.5, 48.6, 43.4, 40.1, 38.6, 36.9, 30.2, 29.8, 25.6, 21.5, 16.1. **HRMS** (ESI+) *m*/*z* calcd. for (C_16_H_25_BrNO, M + H): 326.1114, found: (M + H): 326.1117.

Mixture of *(1S,2R,9R,E)-3′,6,10,10-tetramethyl-4′H-spiro[bicyclo [7.2.0]undecane-2,5′-isoxazol]-5-ene* (**20a**) and *(3aS,6aS,8aR,10aR)-3,8,8,10a-tetramethyl-6-methylene-3a,5,6,6a,7,8,8a,9,10,10a-decahydro-4H-cyclobuta* [5,6]*cyclonona [1,2-d]isoxazole* (**20b**). From 327 mg (1.60 mmol) (−)-β-caryophyllene **1** and 30 mg (0.32 mmol) hydroximoyl chloride **11**, the mixture of compounds **20a** and **20b** in a 55/45 ratio (56 mg, 68%) was obtained as a white crystalline solid.

Mixture of isomers **20a** and **20b: ^1^H NMR** (400 MHz, CDCl_3_): δ 5.10 (bs, 1H), 5.05–5.04 (m, 1H’), 4.91–4.90 (m, 1H’), 2.97–2.86 (m, 1H), 2.86–2.83 (m, 1H’), 2.81–2.70 (m, 1H), 2.42–1.44 (m, 17H + 14H’), 1.33–1.24 (m, 1H + 1H’), 1.17 (s, 3H’), 0.99 (s, 3H), 0.98 (s, 3H’), 0.93 (s, 3H + 3H’). **^13^C NMR** (101 MHz, CDCl_3_) (some carbon signals are not observed due to conformational mobility): δ159.3, 153.9, 151.48, 137.5, 120.5, 111.7, 92.6, 88.4, 58.1, 51.2, 49.6, 48.5, 43.6, 41.3, 40.4, 40.1, 38.7, 36.8, 35.9, 34.2, 31.9, 30.1, 30.0, 29.9, 25.7, 24.3, 24.2, 23.6, 22.0, 21.7, 19.6, 16.01, 13.6, 12.1. **HRMS** (ESI+) *m*/*z* calcd. for (C_17_H_28_NO, M + H): 262.2165, found: (M + H): 262.2168.

*(1S,2R,9R,E)-5′,6,10,10-Tetramethyl-2′-phenyl-2′,4′-dihydrospiro[bicyclo [7.2.0]undecane-2,3′-pyrazol]-5-ene* (**21a**) and *(3aS,6aS,8aR,10aS)-3,8,8,10a-tetramethyl-6-methylene-1-phenyl-1,3a,4,5,6,6a,7,8,8a,9,10,10a-dodecahydrocyclobuta* [6,7]*cyclonona [1,2-c]pyrazole* (**21b**). From 327 mg (1.60 mmol) (−)-β-caryophyllene **1** and 54 mg (0.32 mmol) imidoyl chloride **12**, compound **21b** (44 mg, 41%) was obtained as a light brown solid and compound **21a** (26 mg, 24%) was obtained as a brown solid.

Major isomer **21b: ^1^H NMR** (400 MHz, CDCl_3_): δ 7.26–7.20 (m, 2H), 7.19–7.15 (m, 2H), 7.04–6.99 (m, 1H), 5.07–5.06 (m, 1H), 4.95–4.94 (m, 1H), 2.88 (bs, 1H), 2.48–2.33 (m, 2H), 2.24 (bs, 2H), 2.03–1.94 (m, 1H), 1.99 (s, 3H), 1.89–1.61 (m, 6H), 1.49–1.35 (m, 1H), 1.03 (s, 3H), 1.01 (s, 3H), 0.72 (s, 3H). **^13^C NMR** (101 MHz, CDCl_3_) (some carbon signals are not observed due to conformational mobility): δ 154.0, 151.6, 146.2, 128.4 (2C), 123.2, 122.5 (2C), 111.8, 73.8, 59.1, 51.1, 42.4, 36.4, 34.5, 30.1, 23.4, 22.3, 15.1, 14.7. **HRMS** (ESI+) *m*/*z* calcd. for (C_23_H_33_N_2_, M + H): 337.2638, found: (M + H): 337.2640.

Minor isomer **21a: ^1^H NMR** (400 MHz, CDCl_3_): δ 7.22–7.16 (m, 2H), 7.13–7.06 (m, 2H), 6.80–6.76 (m, 1H), 5.20 (bs, 1H), 3.05 (bs, 1H), 2.93 (bs, 1H), 2.62 (bs, 1H), 2.36–2.18 (m, 2H), 2.15–2.05 (m, 1H), 2.09 (s, 3H), 2.02–1.74 (m, 3H), 1.70 (s, 3H), 1.65–1.53 (m, 4H), 1.50–1.41 (m, 1H), 0.94 (s, 6H). **^13^C NMR** (101 MHz, CDCl_3_) (some carbon signals are not observed due to conformational mobility): δ 146.9, 145.5, 128.8, 121.0, 118.8, 115.4, 74.6, 50.5, 48.2, 42.2, 40.3, 38.2, 37.7, 30.2, 23.9, 22.5, 16.3. **HRMS** (ESI+) *m*/*z* calcd. for (C_23_H_33_N_2_, M + H): 337.2638, found: (M + H): 337.2638.

*(1S,2R,9R,E)-6,10,10-Trimethyl-2′,5′-diphenyl-2′,4′-dihydrospiro[bicyclo [7.2.0]undecane-2,3′-pyrazol]-5-ene* (**22a**) and *(3aR,6aS,8aR,10aR)-8,8,10a-trimethyl-6-methylene-1,3-diphenyl-1,3a,4,5,6,6a,7,8,8a,9,10,10a-dodecahydrocyclobuta* [6,7]*cyclonona [1,2-c]pyrazole* (**22b**). From 327 mg (1.60 mmol) (−)-β-caryophyllene **1** and 74 mg (0.32 mmol) imidoyl chloride **13**, compound **22a** (48 mg, 38%) was obtained as a light yellow solid and compound **22b** (57 mg, 45%) was obtained as a yellow oil.

Isomer **22a: ^1^H NMR** (400 MHz, CDCl_3_): δ 7.86–7.65 (m, 2H), 7.48–7.40 (m, 2H), 7.37–7.22 (m, 5H), 6.91–6.84 (m, 1H), 5.40–5.11 (m, 1H), 3.47 (bs, 1H), 3.38 (bs, 1H), 2.74 (bs, 1H), 2.47–2.29 (m, 2H), 2.24–1.87 (m, 3H), 1.86–1.73 (m, 1H), 1.78 (s, 3H), 1.72–1.51 (m, 4H), 1.49–1.40 (m, 1H), 0.95 (s, 3H), 0.92 (s, 3H). **^13^C NMR** (101 MHz, CDCl_3_) (some carbon signals are not observed due to conformational mobility): δ 144.7, 133.6, 128.9, 128.6, 128.1, 125.5, 121.0, 119.7, 115.9, 75.6, 50.4, 48.1, 40.3, 38.5, 37.6, 30.2, 23.9, 22.4, 16.4. **HRMS** (ESI+) *m*/*z* calcd. for (C_28_H_35_N_2_, M + H): 399.2795, found: (M + H): 399.2798.

Isomer **22b: ^1^H NMR** (400 MHz, CDCl_3_): δ 7.68–7.62 (m, 2H), 7.43–7.37 (m, 2H), 7.36–7.28 (m, 5H), 7.08–7.02 (m, 1H), 5.26–5.25 (m, 1H), 5.11–5.10 (m, 1H), 3.59–3.51 (m, 1H), 2.58–2.48 (m, 2H), 2.15–2.07 (m, 1H), 2.03–1.98 (m, 1H), 1.97–1.75 (m, 6H), 1.71 (dd, *J* = 10.6, 7.8 Hz, 1H), 1.60–1.51 (m, 1H), 1.39 (bs, 3H), 1.06 (s, 3H), 1.05 (s, 3H). **^13^C NMR** (101 MHz, CDCl_3_): δ152.9, 152.5, 144.9, 133.4, 128.6 (2C), 128.4 (2C), 128.1, 126.8 (2C), 122.7, 121.4, 111.6 (2C), 72.8, 57.0, 49.8, 45.8, 38.5, 36.6, 35.7, 33.8, 30.1, 26.9, 24.8, 22.1, 18.5. **HRMS** (ESI+) *m*/*z* calcd. for (C_28_H_35_N_2_, M + H): 399.2795, found: (M + H): 399.2796.

*(1S,2R,9R,E)-5′-(2,6-Dimethoxyphenyl)-6,10,10-trimethyl-2′-(2,4,6-trichlorophenyl)-2′,4′-dihydrospiro[bicyclo [7.2.0]undecane-2,3′-pyrazol]-5-ene* (**23a**) and *(3aR,6aS,8aR,10aR)-3-(2,6-dimethoxyphenyl)-8,8,10a-trimethyl-6-methylene-1-(2,4,6-trichlorophenyl)-1,3a,4,5,6,6a,7,8,8a,9,10,10a-dodecahydrocyclobuta* [6,7]*cyclonona [1,2-c]pyrazole* (**23b**). From 327 mg (1.60 mmol) (−)-β-caryophyllene **1** and 126 mg (0.32 mmol) imidoyl chloride **14**, compound **23a** (77 mg, 43%) and compound **23b** (47 mg, 26%) were obtained as white solids.

Major isomer **23a: ^1^H NMR** (400 MHz, CDCl_3_): δ 7.34 (d, *J* = 2.5 Hz, 1H), 7.32 (d, *J* = 2.5 Hz, 1H), 7.26 (t, *J* = 8.4 Hz, 1H), 6.55 (d, *J* = 8.4 Hz, 2H), 5.22–5.08 (m, 1H), 3.81 (s, 6H), 3.48 (bs, 1H), 3.17 (bs, 1H), 2.61 (bs, 1H), 2.33–2.20 (m, 2H), 2.18–2.11 (m, 1H), 2.10–1.68 (m, 4H), 1.67–1.44 (m, 4H), 1.61 (s, 3H), 0.95 (s, 3H), 0.92 (s, 3H). **^13^C NMR** (101 MHz, CDCl_3_) (some carbon signals are not observed due to conformational mobility): δ 159.1, 137.0, 129.9, 129.6, 128.7, 121.2, 112.1, 104.0, 55.8, 53.9, 47.8, 41.9, 40.5, 36.9, 31.2, 30.6, 29.8, 23.7, 22.6, 16.1. **HRMS** (ESI+) *m*/*z* calcd. for (C_28_H_35_N_2_, M + H): 561.1837, found: (M + H): 561.1837.

Minor isomer **23b: ^1^H NMR** (400 MHz, CDCl_3_): δ 7.34 (d, *J* = 2.5 Hz, 1H), 7.32 (d, *J* = 2.5 Hz, 1H), 7.26 (t, *J* = 8.4 Hz, 1H), 6.55 (d, *J* = 8.4 Hz, 2H), 5.01–5.00 (m, 1H), 4.72–4.71 (m, 1H), 3.80 (s, 6H), 3.37–3.32 (m, 1H), 2.38 (dd, *J* = 18.0, 9.5 Hz, 1H), 2.03–1.86 (m, 3H), 1.81–1.49 (m, 7H), 1.37–1.24 (m, 1H), 1.26 (s, 3H), 1.02 (s, 3H), 0.98 (s, 3H). **^13^C NMR** (101 MHz, CDCl_3_): δ159.5 (2C), 152.3, 151.9, 138.8, 138.2, 138.1, 132.0, 130.1, 129.5, 128.9, 111.2, 110.9, 103.6 (2C), 75.2, 59.6, 55.8 (2C), 51.6, 43.3, 42.1, 36.9, 36.8, 34.4, 29.9, 24.7, 22.4, 22.0, 17.1. **HRMS** (ESI+) *m*/*z* calcd. for (C_30_H_36_Cl_3_N_2_O_2_, M + H): 561.1841, found: (M + H): 561.1837.

Mixture of *(1S,2R,9R,E)-6,10,10-trimethyl-2′-(4-nitrophenyl)-5′-phenyl-2′,4′-dihydrospiro[bicyclo [7.2.0]undecane-2,3′-pyrazol]-5-ene* (**24a**) and *(3aR,6aS,8aR,10aR)-8,8,10a-trimethyl-6-methylene-1-(4-nitrophenyl)-3-phenyl-1,3a,4,5,6,6a,7,8,8a,9,10,10a-dodecahydrocyclobuta* [6,7]*cyclonona [1,2-c]pyrazole* (**24b**). From 327 mg (1.60 mmol) (−)-β-caryophyllene **1** and 88 mg (0.32 mmol) imidoyl chloride **15**, the mixture of compounds **24a** and **24b** in a 59/41 ratio (77 mg, 54%) was obtained as an orange oil.

Mixture of isomers **24a** and **24b: ^1^H NMR** (400 MHz, CDCl_3_): δ 8.15–8.08 (m, 2H+2H’), 7.87–7.80 (m, 2H), 7.71–7.66 (m, 2H’), 7.51–7.25 (m, 5H + 5H’), 5.38–5.20 (m, 1H), 5.30–5.29 (m, 1H’), 5.12–5.11 (m, 1H’), 3.64 (dd, *J* = 10.4, 2.3 Hz, 1H’), 3.63–3.40 (m, 1H), 2.93–2.73 (m, 1H), 2.71–2.43 (m, 2H + 3H’), 2.22–1.39 (m, 12H + 12H’), 1.29–1.21 (m, 1H), 1.06 (s, 3H’), 1.04 (s, 3H’), 0.91 (s, 3H), 0.88 (s, 3H). **^13^C NMR** (101 MHz, CDCl_3_) (some carbon signals are not observed due to conformational mobility): δ155.3, 151.6, 150.2, 148.8, 139.0, 138.7, 138.0, 132.1, 131.8, 129.6, 129.3, 128.8, 128.6, 127.1, 126.1, 125.8, 125.5, 120.5, 113.9, 112.7, 112.0, 77.4, 75.9, 71.6, 57.2, 51.7, 49.3, 48.2, 47.3, 40.2, 38.3, 37.0, 36.8, 36.0, 34.0, 31.5, 30.1, 29.9, 27.5, 25.6, 23.6, 22.2, 21.8, 17.9, 16.4. **HRMS** (ESI+) *m*/*z* calcd. for (C_28_H_34_N_3_O_2_, M + H): 444.2646, found: (M + H): 444.2646.

#### 3.4.6. General Procedure for Synthesis of Compounds **25**–**31**

A mixture of 0.16 mmol of (−)-β-caryophyllene **1** (1 eq.) and 0.32–0.48 mmol hydroxymoyl halide or imidoyl chloride (2–3 eq.) in 3 mL of chloroform was placed into a 15 mL vial (diameter 1.3 cm). This vial was then placed in a closed 50 mL vial (diameter 3.5 cm) containing amine (35.85 mmol, ~5 mL, triethylamine for hydroxymoyl halide or DIPEA for imidoyl chloride) and the reaction mixture was stirred at room temperature for 2–4 days (TLC or NMR control). After the completion of the reaction as monitored by TLC and NMR control, the mixture from the inner vial was diluted with 10 mL of chloroform, transferred to a separating funnel, and washed with 2% aqueous HCl (2 × 10 mL). The organic phase was dried over anhydrous Na_2_SO_4_, the solvent was removed under reduced pressure, and the residue was purified by column chromatography on silica gel using chloroform (for compounds **27**, **29**, and **30**), methanol/chloroform mixtures (1:800, for compounds **25**, **26**, and **30**) or methanol (for compound **28**) as eluents.

*(3aR,6R,6aS,8aR,10aS)-3,3′-Bis(4-chlorophenyl)-8,8,10a-trimethyl-3a,4,5,6a,7,8,8a,9,10,10a-decahydro-4′H-spiro[cyclobuta* [5,6]*cyclonona [1,2-d]isoxazole-6,5′-isoxazole]* (**25**). From 33 mg (0.16 mmol) (−)-β-caryophyllene **1** and 61 mg (0.32 mmol) hydroximoyl chloride **7**, compound **25** (61 mg, 74%) was obtained as a pale yellow oil.

**^1^H NMR** (400 MHz, CDCl_3_): δ 7.65–7.55 (m, 2H), 7.43–7.45 (m, 2H), 7.44–7.36 (m, 4H), 3.35 (d, *J* = 16.1 Hz, 1H), 3.18–3.13 (m, 1H), 3.14 (d, *J* = 16.1 Hz, 1H), 2.53 (q, *J* = 9.4 Hz, 1H), 2.20–2.04 (m, 2H), 1.96–1.62 (m, 7H), 1.61–1.52 (m, 1H), 1.57 (s, 3H), 1.32–1.25 (m, 1H), 1.01 (s, 3H), 0.98 (s, 3H). **^13^C NMR** (101 MHz, CDCl_3_) (some carbon signals are not observed due to conformational mobility): δ 159.1, 153.9, 136.1, 135.9, 129.3, 129.2, 128.8, 128.4, 128.4, 127.6, 92.5, 89.9, 50.2, 47.1, 36.7, 36.3, 35.3, 34.0, 30.1, 22.6, 22.5, 19.8. **HRMS** (ESI+) *m*/*z* calcd. for (C_29_H_33_Cl_2_N_2_O_2_, M + H): 511.1914, found: (M + H): 511.1916.

*(3aR,6R,6aS,8aR,10aS)-3,3′-Bis(4-methoxyphenyl)-8,8,10a-trimethyl-3a,4,5,6a,7,8,8a,9,10,10a-decahydro-4′H-spiro[cyclobuta* [5,6]*cyclonona [1,2-d]isoxazole-6,5′-isoxazole]* (**26**). From 33 mg (0.16 mmol) (−)-β-caryophyllene **1** and 59 mg (0.32 mmol) hydroximoyl chloride **8**, compound **26** (58 mg, 72%) was obtained as a pale yellow oil.

**^1^H NMR** (400 MHz, CDCl_3_): δ 7.67–7.60 (m, 2H), 7.54–7.47 (m, 2H), 7.00–6.92 (m, 4H), 3.86 (s, 3H), 3.84 (s, 3H), 3.37 (d, *J* = 16.1 Hz, 1H), 3.20 (d, *J* = 16.1 Hz, 1H), 3.17–3.12 (m, 1H), 2.58–2.47 (m, 1H), 2.17–2.04 (m, 2H), 1.93–1.73 (m, 6H), 1.71–1.61 (m, 1H), 1.60–1.51 (m, 1H), 1.54 (s, 3H), 1.33 (t, *J* = 10.9 Hz, 1H), 1.00 (s, 3H), 0.97 (s, 3H). **^13^C NMR** (101 MHz, CDCl_3_) (some carbon signals are not observed due to conformational mobility): δ 161.0, 160.7, 159.8, 154.5, 128.6, 127.9, 122.6, 122.5, 114.3, 114.2, 91.7, 89.1, 77.4, 55.5, 55.4, 50.4, 47.1, 36.8, 36.6, 35.3, 33.9, 30.0, 22.8, 22.5, 19.7. **HRMS** (ESI+) *m*/*z* calcd. for (C_31_H_39_N_2_O_4_, M + H): 503.2904, found: (M + H): 503.2910.

*2,2′-((3aS,6R,6aS,8aR,10aR)-8,8,10a-Trimethyl-3a,4,5,6a,7,8,8a,9,10,10a-decahydro-4′H-spiro[cyclobuta* [5,6]*cyclonona [1,2-d]isoxazole-6,5′-isoxazole]-3,3′-diyl)bis(4-bromophenol)* (**27**). From 33 mg (0.16 mmol) (−)-β-caryophyllene **1** and 80 mg (0.32 mmol) hydroximoyl chloride **9**, compound **27** (63 mg, 62%) was obtained as a white solid.

**^1^H NMR** (400 MHz, CDCl_3_): δ 9.90 (s, 1H), 9.87 (bs, 1H), 7.71–7,69 (m, 1H), 7.40 (m, 2H), 7.33–7.31 (m, 1H), 6.97–6.91 (m, 2H), 3.52 (d, *J* = 16.0 Hz, 1H), 3.40 (d, *J* = 16.0 Hz, 1H), 3.18–3.13 (m, 1H), 2.59–2.51 (m, 1H), 2.39–2.30 (m, 1H), 2.20–2.13 (m, 1H), 2.07–1.97 (m, 1H), 1.95–1.89 (m, 1H), 1.86–1.73 (m, 5H), 1.71–1.63 (m, 1H), 1.61 (s, 3H), 1.30–1.23 (m, 1H), 1.02 (s, 3H), 1.00 (s, 3H). **^13^C NMR** (101 MHz, CDCl_3_): δ 159.2, 156.8, 156.5, 155.9, 134.4, 133.6, 130.0, 129.5, 119.8, 118.9, 115.8, 115.5, 111.4, 111.4, 92.2, 89.3, 50.3, 49.2, 46.4, 39.9, 36.5, 36.2, 35.6, 33.8, 29.9, 26.7, 22.8, 22.3, 19.3.

*(3aR,6R,6aS,8aR,10aR)-3,3′-Dibromo-8,8,10a-trimethyl-3a,4,5,6a,7,8,8a,9,10,10a-decahydro-4′H-spiro[cyclobuta* [5,6]*cyclonona [1,2-d]isoxazole-6,5′-isoxazole]* (**28**). From 33 mg (0.16 mmol) (−)-β-caryophyllene **1** and 97 mg (0.48 mmol) hydroximoyl bromide **10**, compound **28** (63 mg, 83%) was obtained as a white solid.

**^1^H NMR** (400 MHz, CDCl_3_): δ3.22 (d, *J* = 17.2 Hz, 1H), 3.01 (d, *J* = 17.2 Hz, 1H), 2.74 (dd, *J* = 10.2, 5.0 Hz, 1H), 2.49–2.40 (m, 1H), 2.32–2.23 (m, 1H), 2.07–1.84 (m, 4H), 1.82–1.64 (m, 5H), 1.30 (s, 3H), 1.27–1.20 (m, 1H), 1.01 (s, 3H), 0.99 (s, 3H). **^13^C NMR** (101 MHz, CDCl_3_) (some carbon signals are not observed due to conformational mobility): δ 134.6, 92.9, 90.2, 53.3, 47.4, 44.3, 36.5, 34.8, 34.6, 34.6, 30.0, 22.5, 21.7, 19.6. **HRMS** (ESI+) *m*/*z* calcd. for (C_17_H_25_Br_2_N_2_O_2_, M + H): 477.0277, found: (M + H): 477.0279.

*(3aR,6R,6aS,8aR,10aR)-8,8,10a-trimethyl-1,2′,3,5′-tetraphenyl-2′,3a,4,4′,5,6a,7,8,8a,9,10,10a-Dodecahydro-1H-spiro[cyclobuta* [6,7]*cyclonona [1,2-c]pyrazole-6,3′-pyrazole]* (**29**). From 33 mg (0.16 mmol) (−)-β-caryophyllene **1** and 111 mg (0.48 mmol) imidoyl chloride **13**, compound **29** (65 mg, 69%) was obtained as a yellow oil.

**^1^H NMR** (400 MHz, CDCl_3_): δ 7.85–7.79 (m, 2H), 7.68–7.60 (m, 2H), 7.51–7.25 (m, 14H), 7.16–7.08 (m, 1H), 7.98–7.90 (m, 1H), 3.61 (d, *J* = 16.4 Hz, 1H), 3.51–3.46 (m, 1H), 3.47 (d, *J* = 16.4 Hz, 1H), 3.06–2.94 (m, 1H), 2.47–2.25 (m, 2H), 2.24–1.95 (m, 5H), 1.86–1.72 (m, 1H), 1.71–1.61 (m, 1H), 1.56–1.45 (m, 2H), 1.22–1.05 (m, 3H), 1.03 (s, 3H), 0.97 (s, 3H). **^13^C NMR** (101 MHz, CDCl_3_) (some carbon signals are not observed due to conformational mobility): δ 153.3, 145.6, 144.7, 144.5, 133.8, 133.1, 129.0, 128.8, 128.7, 128.6, 128.5, 127.2, 125.4, 123.9, 122.9, 120.8, 117.4, 77.4, 74.8, 50.1, 46.7, 38.0, 36.3, 35.0, 33.5, 30.4, 23.7, 22.5, 17.8. **HRMS** (ESI+) *m*/*z* calcd. for (C_41_H_45_N_4_, M + H): 593.3639, found: (M + H): 593.3642.

*(3aR,6R,6aS,8aR,10aR)-3,5′-Bis(2,6-dimethoxyphenyl)-8,8,10a-trimethyl-1,2′-bis(2,4,6-trichlorophenyl)-2′,3a,4,4′,5,6a,7,8,8a,9,10,10a-dodecahydro-1H-spiro[cyclobuta* [6,7]*cyclonona [1,2-c]pyrazole-6,3′-pyrazole]* (**30**). From 33 mg (0.16 mmol) (−)-β-caryophyllene **1** and 190 mg (0.48 mmol) imidoyl chloride **14**, compound **30** (108 mg, 73%) was obtained as a colorless oil.

**^1^H NMR** (400 MHz, CDCl_3_): δ7.34 (d, *J* = 2.5 Hz, 1H), 7.33–7.31 (m, 2H), 7.30–7.21 (m, 2H), 7.19 (d, *J* = 2.5 Hz, 1H), 6.57 (d, *J* = 8.4 Hz, 3H), 6.40 (d, *J* = 8.3 Hz, 1H), 3.81–3.74 (m, 9H), 3.59 (s, 3H), 3.41 (d, *J* = 17.5 Hz, 1H), 3.30–3.22 (m, 1H), 3.05 (d, *J* = 17.5 Hz, 1H), 2.77–2.66 (m, 1H), 2.14–2.00 (m, 3H), 1.98–1.86 (m, 2H), 1.73–1.53 (m, 4H), 1.45–1.33 (m, 2H), 1.23 (s, 3H), 1.05 (s, 3H), 0.99 (s, 3H). **^13^C NMR** (101 MHz, CDCl_3_) (some carbon signals are not observed due to conformational mobility): δ 158.7, 158.0, 157.6, 149.7, 149.1, 138.4, 138.3, 137.5, 137.3, 136.9, 136.9, 131.2, 131.1, 129.1, 129.0, 128.6, 128.5, 127.9, 127.5, 110.8, 110.2, 103.2, 102.9, 102.2, 73.6, 72.9, 54.9, 54.7, 54.2, 50.8, 48.0, 43.1, 34.5, 34.0, 32.8, 28.9, 23.9, 21.5, 20.5, 15.9. **HRMS** (ESI+) *m*/*z* calcd. for (C_45_H_47_Cl_6_N_4_O_4_, M + H): 917.1723, found: (M + H): 917.1718.

*(3aR,6R,6aS,8aR,10aR)-8,8,10a-Trimethyl-1,2′-bis(4-nitrophenyl)-3,5′-diphenyl-2′,3a,4,4′,5,6a,7,8,8a,9,10,10a-dodecahydro-1H-spiro[cyclobuta* [6,7]*cyclonona [1,2-c]pyrazole-6,3′-pyrazole]* (**31**). From 33 mg (0.16 mmol) (−)-β-caryophyllene **1** and 88 mg (0.32 mmol) imidoyl chloride **15**, compound **31** (22 mg, 20%) was obtained as an orange solid.

**^1^H NMR** (400 MHz, CDCl_3_): δ 8.17–8.13 (m, 4H), 7.85–7.81 (m, 2H), 7.68–7.64 (m, 2H), 7.55–7.42 (m, 6H), 7.38–7.30 (m, 4H), 3.70 (d, *J* = 16.8 Hz, 1H), 3.54–3.46 (m, 2H), 3.17–3.10 (m, 1H), 2.81–2.72 (m, 1H), 2.51 (bs, 1H), 2.24–2.16 (m, 1H), 2.16–1.85 (m, 5H), 1.79–1.58 (m, 4H), 1.41–1.35 (m, 1H), 1.31–1.25 (m, 1H), 0.98 (s, 3H), 0.96 (s, 3H). **^13^C NMR** (101 MHz, CDCl_3_) (some carbon signals are not observed due to conformational mobility): δ 154.7, 149.5, 148.5, 139.4, 132.5, 131.7, 130.0, 129.8, 129.0, 127.3, 126.0, 125.9, 125.5, 125.3, 115.2, 113.4, 74.3, 52.6, 47.5, 39.5, 36.5, 34.7, 33.9, 30.1, 22.8, 17.7. **HRMS** (ESI+) *m*/*z* calcd. for (C_41_H_43_N_6_O_2_, M + H): 683.3340, found: (M + H): 683.3336.

### 3.5. Cytotoxicity (MTT-Test)

#### 3.5.1. Initial Testing

To evaluate the cytotoxicity of the compounds, we placed cells in (20–30) × 10^3^ cells/mL concentrations in 96-well culture plates for 24 h. Cells were counted after treatment with Trypan blue solution (0.4%). They were then exposed to two different concentrations of compounds **16**–**30** (50–100 µM) dilutions in pre-incubated cells at 37 °C for 72 h. In control wells with untreated cells, only (DMSO + PBS) was added. Cell viability was measured by the standard MTT test [56]. The stock solutions of each compound were prepared in DMSO following by preparation of serial dilutions in PBS.

The absorbance was measured at 540 nm using a Multiskan™ FC microplate photometer and the Skanlt software 6.1 RE for microplate reader, both from Thermo Scientific (Waltham, MA, USA). Experiments were carried out in triplicate; to perform the statistical analysis, more concentrations should be evaluated.

#### 3.5.2. IC_50_ Determination

To evaluate the cytotoxicity of the SDE-series in vitro, we placed cells in (15–30) × 10^3^ cells/mL concentrations in 96-well culture plates for 24 h. Cells were counted after treatment with Trypan blue solution (0.4%). After 24 h, the cells were exposed to six tested concentrations of SDE-series (50–1.5 µM) dilutions to pre-incubated cells at 37 °C for 72 h. In control wells with untreated cells, only (DMSO + PBS) was added. Cell viability was measured by the standard MTT test. The absorbance was measured at 540 nm using a Multiskan™ FC microplate photometer and the Skanlt software 6.1 RE for microplate reader, both from Thermo Scientific (Waltham, MA, USA). In vitro experiments were carried out in triplicate. GraphPad prism version 9.0 was used to determine the IC_50_. The IC_50_ data are presented as mean (±) standard deviation (SD).

#### 3.5.3. Viruses and Cells

We used MDCK cells (ATCC CCL-34) from the collection of cell lines of the Saint Petersburg Pasteur Institute. Cells were cultured in 96-well culture plates in MEM medium with 10% fetal bovine serum («HyClone», Cytiva, Wilmington, DE, USA), 40 U/mL gentamicin sulfate, and 2.5 U/mL amphotericin B. A cell suspension with a concentration of 105 cells/mL was placed in the wells of the plates in a volume of 100 μL and cultured until a complete monolayer was formed for 24 h at 36 °C in the presence of 5% CO_2_. The same medium without serum was used as a support medium for culturing cells with viruses.

We used influenza virus A/Puerto Rico/8/34 (H1N1) from the collection of the Saint Petersburg Pasteur Institute. The infectious titers of the virus were determined by titration in 96-well plates with monolayers of MDCK cells. The results were evaluated visually according to the presence of the virus cytopathic action; the virus titer was calculated by the Spearman–Kerber method and represented in decimal logarithms of 50% tissue cytopathic doses in mL (lg TCD_50/mL_).

The stock solutions of each compound were prepared in DMSO followed by the preparation of serial dilutions in the cell culture medium (MEM).

#### 3.5.4. Evaluation of Cytotoxic Properties of Compounds

The assessment of the toxicity of compounds was carried out based on the evaluation of the cell viability using the reduction reaction of the tetrazolium dye MTT (3-(4,5-dimethyl-2-thiazolyl)-2,5-diphenyl-2H-tetrazolium bromide) by cells in culture. Its intensity shows the degree of cell viability as a result of dye reduction by mitochondrial and partially cytoplasmic dehydrogenases.

The test compounds in the concentration range of 3.7–300 μg/mL dissolved in the medium for cell cultivation were added to the plate wells in a volume of 200 μL and incubated for 48 h at 36 °C in an atmosphere of 5% CO_2_. At the end of the incubation period, the cells were washed with the MEM medium, and 100 μL of a solution (0.5 mg/mL) of MTT in the cell medium was added to the plate wells. The cells were incubated at 36 °C at 5% CO_2_ for 2 h and washed for 5 min with saline. The precipitate was dissolved in 100 μL of DMSO per well, and the optical density was measured using a Multiscan FC plate analyzer (Thermo Scientific) at a wavelength of 540 nm. Based on the obtained data, the 50% cytotoxic concentration (CC_50_) was calculated, i.e., the concentration of the compound which reduces the optical density in the wells by half compared to the control cells without drugs.

#### 3.5.5. Evaluation of Antiviral Activity of Compounds

The compounds in appropriate concentrations were added to the MDCK cells (0.1 mL per well). After 1 h of incubation, the cells were infected with influenza virus A/Puerto Rico/8/34 (H1N1) (m.o.i. 0.01) and incubated for 48 h at 36 °C and 5% CO_2_. After that, cell viability was assessed by the MTT test, as described above. The cytoprotective activity of the compounds was considered as their ability to increase the values of OD compared to the control wells (with virus only, no drugs). Based on the results obtained, the values of IC_50_, i.e., the concentration of compounds that resulted in 50% cell protection, were calculated using the GraphPad Prism 6.01 software. The values of IC_50_ obtained in μg/mL were then calculated into micromoles (μM). For each compound, the value of selectivity index (SI) was calculated as the ratio of CC_50_ to IC_50_. Compounds with SI of 10 and higher were considered active. Neuraminidase inhibitors zanamivir (Relenza, Glaxo Wellcome, Évreux, France) and oseltamivir carboxylate (Hoffman LaRoche, Basel, Switzerland) were used as the reference compounds.

#### 3.5.6. Time-of-Addition Experiments

To determine the stage of the viral life cycle that is affected by the compound, cells were seeded into 24-well plates and incubated with influenza virus A/Puerto Rico/8/34 (H1N1) (m.o.i. 10) for 1 h at 4 °C. After washing of the non-absorbed virions for 5 min with MEM, the plates were incubated for 8 h at 36 °C at 5% CO_2_. The starting point of this incubation was referred as 0 h. The lead compounds **3q** and **3s** (final concentration 300 micromole/L) were dissolved in MEM and added to the infected cells at the time periods as follows: (−2) h (before infecting); (−1) h (simultaneously to absorption); and at 0, 2, 4, or 6 h post infection (hpi). The treatment (−2) to 8 hpi was considered as a positive control. After 8 h of growth, the infectious titer of the virus was determined in the culture medium by an end-point dilution assay.

#### 3.5.7. Anti-Neuraminidase Assay

To assess the ability of compounds to inhibit neuraminidase activity, the activity of viral NA was measured in the presence of compounds in the reaction with fluorogenic substrate [57]. For this purpose, serial dilutions of compounds under investigation in the buffer (32.5 mM MES, pH 6.0, 4 mM CaCl_2_) were mixed with influenza virus A/Puerto Rico/8/34 (H1N1) in the wells of black 96-well plates (Corning, Corning, NY, USA). Plates were incubated 30 min at 37 °C. Then 0.2 mM substrate solution (4-methylumbelliferyl-α-d-*N*-acetylneuraminic acid) was added to the wells and the plates were further incubated for 30 min at room temperature. The reaction was stopped with the stop-solution (25% ethanol, 0.1 M glycine pH 10.7), followed by measurement of luminescence on the multifunctional plate reader Varioscan (ThermoFisher Scientific, excitation λ 365 nm, emission λ 450 nm). The clinically approved NA inhibitor zanamivir was used as the reference compound. Based on the data obtained, 50% inhibiting concentrations of each compound were calculated.

## 4. Conclusions

Thus, the possibility of introducing five-membered fused and spiro-linked heterocycles into the structure of sesquiterpenes by the 1,3-dipolar cycloaddition reactions of nitrile oxides and nitrilimines to caryophyllene was demonstrated. The use of the diffusion mixing technique, which is effective in the case of cycloaddition of 1,3-dipoles to low-reactive olefins, made it possible to obtain the target products in good yields. High diastereoselectivity of the 1,3-dipolar cycloaddition to caryophyllene was demonstrated, regardless of the type of substituent in the initial dipole, which is an advantage of this reaction compared to the literature-described [4 + 2] cycloaddition reactions of the quinone methides to this terpene. The high regioselectivity of the 1,3-dipoles interaction with caryophyllene was also discovered.

The study of antiviral and cytotoxic activity for some heterocyclic derivatives synthesized in this work revealed relatively high biological activity of previously little-studied cycloaddition adducts at the exocyclic C=CH_2_ bond of caryophyllene. The effect of substituents in synthesized heterocycles on biological activity was demonstrated, and compounds with a good inhibitory effect on the H1N1 influenza virus, comparable to commercially available drugs, were obtained. The activity of the compound was demonstrated up to 6 h post infection, and this could be due to a slight inhibiting activity against the viral neuraminidase, necessary at the stage of progeny virion budding.

## Figures and Tables

**Table 1:** Ratio and yield of caryophyllene cycloaddition products.

Products	a:b ^a^	Yield a, %	Yield b, %
**16a + 16b**	1.9:1	56	29
**17a + 17b**	1.8:1	46	25
**18a + 18b**	1.2:1	34	16
**19a + 19b**	0.3:1	9	11
**20a + 20b**	1.2:1	68 ^b^	
**21a + 21b**	0.8:1	24	41
**22a + 22b**	1:1	38	45
**23a + 23b**	1.7:1	43	26
**24a + 24b**	1.4:1	54 ^b^	

^a^—A ratio determined from the ^1^H NMR spectra of reaction mixtures. ^b^—Isomers could not be separated; the yield is indicated for a mixture of **a** and **b**.

**Table 2:** Cytotoxic and anti-viral properties of novel (−)-β-caryophyllene derivatives against influenza virus A/Puerto Rico/8/34 (H1N1) in MDCK cells. Compounds with maximum selectivity indices are highlighted in gray.

Compound	CC_50_, μM	IC_50_, μM	SI
**16a**	125.40 ± 6.5	9.8 ± 1.3	13
**16b**	20.21 ± 1.9	16.8 ± 2.2	1
**17a**	36.97 ± 2.2	8.03 ± 1.1	5
**17b**	27.01 ± 2.4	>12.8	<2
**18a**	25.11 ± 1.8	4.78 ± 0.6	5
**19a**	19.64 ± 1.1	>12.26	<2
**19b**	44.17 ± 3.6	>33.7	<1
**21a**	27.97 ± 1.9	6.25 ± 0.7	4
**21b**	374.70 ± 15.6	>296.7	<1
**22a**	170.4 ± 12.1	72.7 ± 9.2	2
**22b**	25.08 ± 1.6	>9.28	<3
**23a**	10.16 ± 0.8	>5.59	<2
**23b**	>535	178.25 ± 20.1	>3
**25**	426.43 ± 26.9	34.15 ± 4.4	12
**26**	>652	>652	-
**28**	13.83 ± 1.2	>8.70	<2
**29**	>506	101.7 ± 12.6	>5
**30**	>330	71.05 ± 8.8	>5
Rimantadine	335 ± 27	67.0 ± 4.9	5
Amantadine	284 ± 21	64 ± 5	4
Deitiforin	1266 ± 82	209 ± 15	6
Ribavirin	>2000	25	>81.0

CC_50_—50% cytotoxicity concentration, at which 50% of cells in uninfected monolayers are destroyed. IC_50_—50% virus inhibitory concentration, at which 50% of cells in infected monolayers are preserved. SI—selectivity index, ratio CC_50_/IC_50_.

**Table 3:** Initial testing of compounds **16**–**30** cytotoxicity on different cell lines (MTT test).

Compound	Human Cancer Cell Lines		Human Healthy Cells		Murine Mammary Cell Line
	DU145	A549	VA-13	HEK293	EMT6
**16a**	−	−	−	−	−
**16b**	−	−	−	−	−
**17a**	+ *	+ *	+ *	+ *	+ *
**17b**	+	+	+	+	+
**18a**	−	−	−	−	−
**18b**	−	−	+^	+^	−
**19a**	−	−	−	−	−
**21a**	+ *	+ *	+ *	+ *	+ *
**21b**	−	−	−	−	−
**22a**	−	−	+^	+^	−
**22b**	−	−	+	+	−
**23a**	+	+	+	+	+ *
**23b**	−	−	−	−	−
**25**	+^	+^	−	−	−
**26**	−	−	−	−	−
**28**	−	−	+^	+^	−
**29**	−	−	−	−	−
**30**	−	−	−	−	−

+ IC_50_ < 50 µM. − IC_50_ > 50 µM. * The compound is extremely toxic, killing all cells within 48 h. +^ IC_50_ ranging between 50 and 100 µM.

**Table 4:** Cytotoxicity of compounds **17a**, **21a**, **23a**, and **25** (IC_50_, MTT test).

Organ	Cell Lines	IC_50_, µM			
		17a	21a	23a	25
Colon cancer	HCT116	39.4 ± 2.8	22.4 ± 0.8	>50	>50
	HT-29	47.1 ± 3.0	39.4 ± 2.1	45.0 ± 1.9	>50
Breast cancer	MCF7	>50	>50	>50	>50
	SKBR3	36.5 ± 3.2	12.3 ± 1.0	>50	>50
Melanoma	SK-MEL-28	38.9 ± 3.9	24.9 ± 1.3	>50	>50
Lung cancer	A549	30.7 ± 2.1	21.2 ± 0.9	33.4 ± 2.2	>50
Prostate cancer	DU145	42.6 ± 3.4	18.8 ± 0.4	28.8 ± 1.2	>50
Normal cells	HEK-293	44.3 ± 4.1	14.2 ± 0.9	39.2 ± 3.0	>50

## Data Availability

Data are available from the authors upon request.

## Supplementary Materials

The following supporting information can be downloaded at: https://www.mdpi.com/article/10.3390/ijms252111435/s1.
