# Supplementary material for: 1,3-Dipolar Cycloaddition of Nitrile Oxides and Nitrilimines to (−)-β-Caryophyllene: Stereoselective Synthesis of Polycyclic Derivatives and Their Biological Testing
Source: Int J Mol Sci. 2024 Oct 24;25(21):11435. doi: 10.3390/ijms252111435 (PMC11545984; doi:10.3390/ijms252111435)
Supplement: Supplementary file 1 [file ijms-25-11435-s001.zip › ijms-3273476-supplementary.pdf]

Supporting Information

1,3-Dipolar cycloaddition of nitrile oxides and nitrilimines to (−)-β-caryophyllene: stereoselective synthesis of polycyclic derivatives and their biological testing

Dmitry E. Shybanov[1], Maxim E. Kukushkin[1], Yuri K. Grishin[1], Vitaly A. Roznyatovsky[1], Viktor A. Tafeenko[1], Louay Abo Qoura [2,3], Vadim S. Pokrovsky[2,3], Olga I. Yarovaya[4], Svetlana V. Belyaevskaya[5], Alexandrina S. Volobueva[5], Iana L. Esaulkova[5], Vladimir V. Zarubaev[5], Elena K. Beloglazkina[1]

[1)] *Department of Chemistry, M. V. Lomonosov Moscow State University, 119991 Moscow, Russia*
[2)] *Research Institute of Molecular and Cellular Medicine, People's Friendship University of Russia (RUDN University), 117198 Moscow, Russia*
[3)] *N.N.Blokhin National Medical Research Center of Oncology of Ministry of Health of Russian Federation, 115478 Moscow, Russia*
[4)] *N.N. Vorozhtsov Novosibirsk Institute of Organic Chemistry, Siberian Branch, Russian Academy of Sciences, Lavrentjev Avenue 9, 630090 Novosibirsk, Russia*
[3)] Pasteur Research Institute of Epidemiology and Microbiology, 14 MiraStr, St. Petersburg, 197101 Russia

Table of Contents

NMR spectra of compounds 9, 14, 16-33. 3

Table S1. Crystal data and structure refinement for compound 18a. 49

Table S2. Hydrogen bonds for 18a [Å and °]. 50

Table S3. Crystal data and structure refinement for compound 21a. 51

Table S4. Crystal data and structure refinement for compound 21b 52

Table S5. Cytotoxic effect of compounds 17a (SDE-1525), 21a (SDE-1558-1), 23a (SDE-1615-1), and 25 (SDE-1650-2) on a variety of cell lines, including colon cancer (HCT116, HT-29), breast cancer (MCF7, SKBR3), melanoma (SK-MEL-28), lung cancer (A549), prostate cancer (DU145), and normal cell (HEK-293), with IC_{50} values. 53

NMR spectra of compounds 9, 14, 16-33.

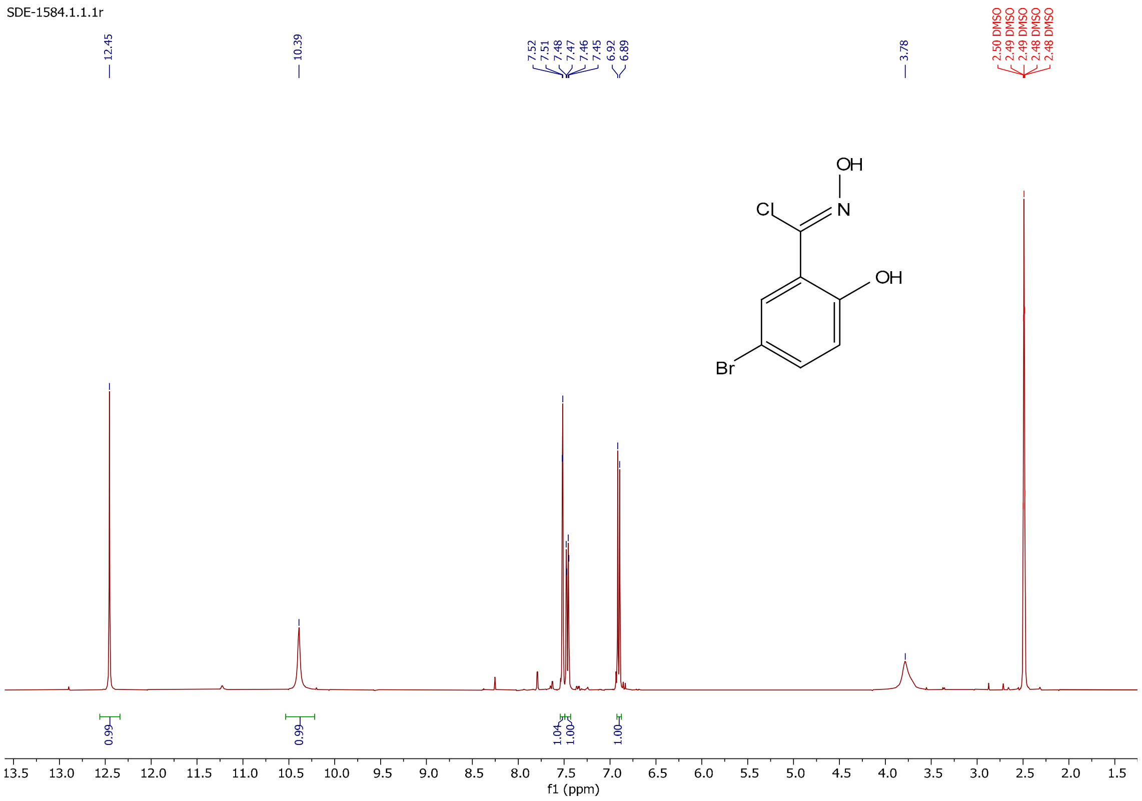

Figure S1. ^{1}H NMR spectra of compound **9.**

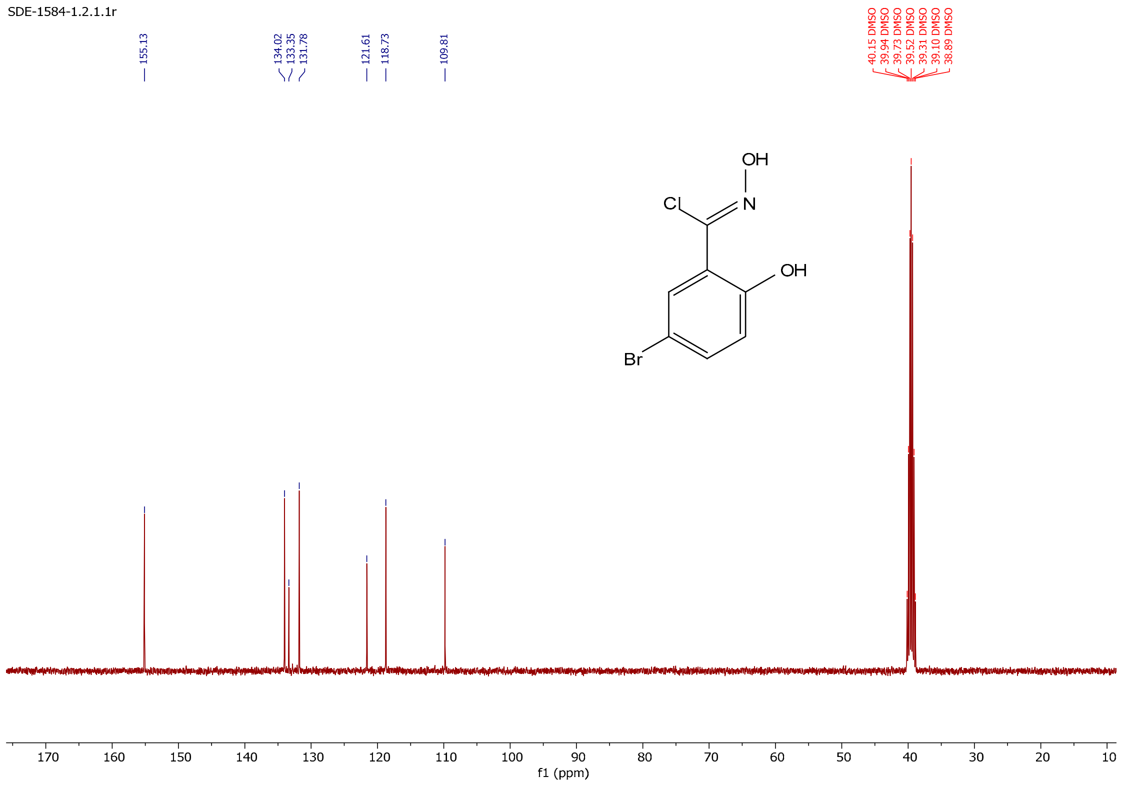

Figure S2. ^{13}C NMR spectra of compound **9.**

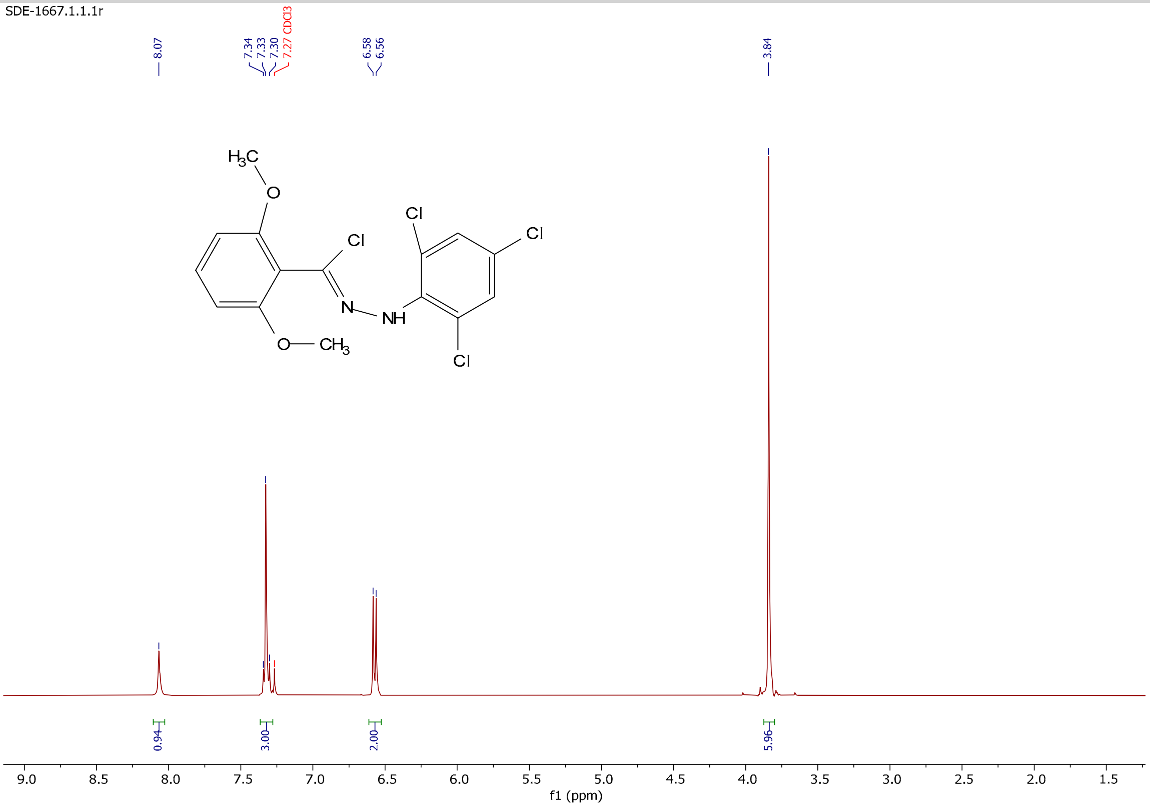

Figure S3. ^{1}H NMR spectra of compound **14.**

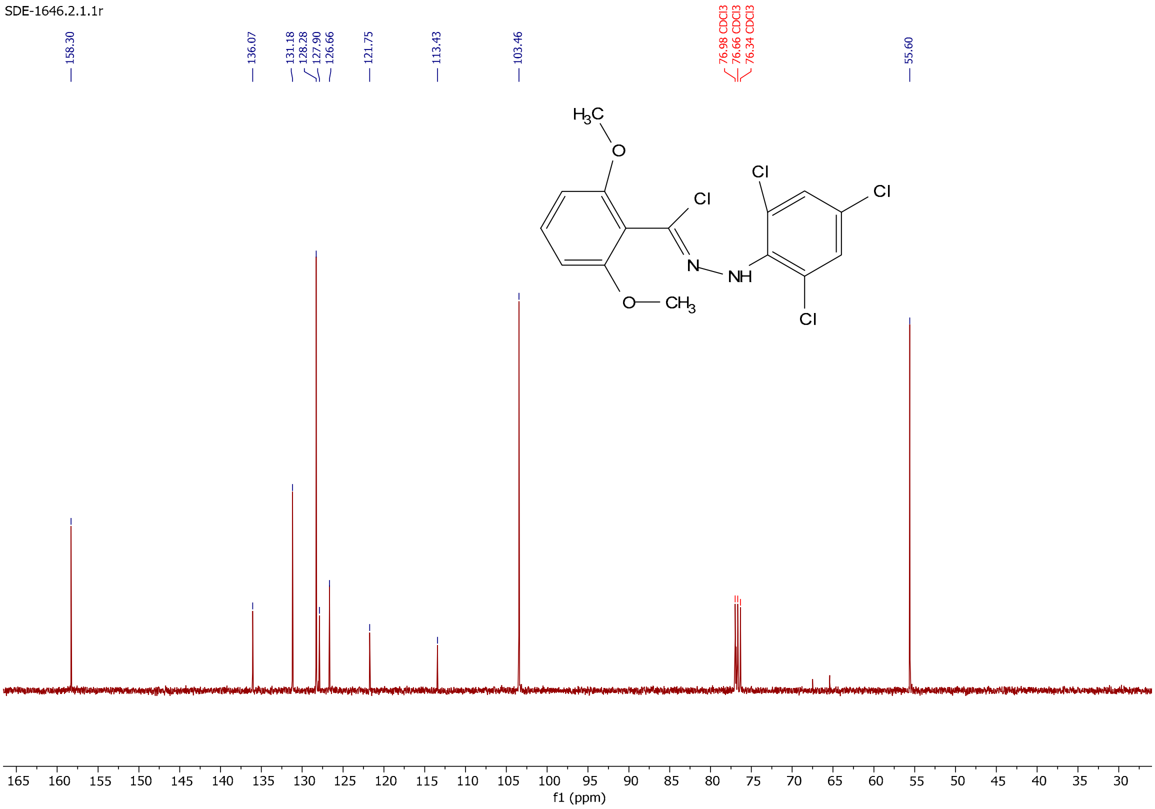

Figure S4. ^{13}C NMR spectra of compound **14.**

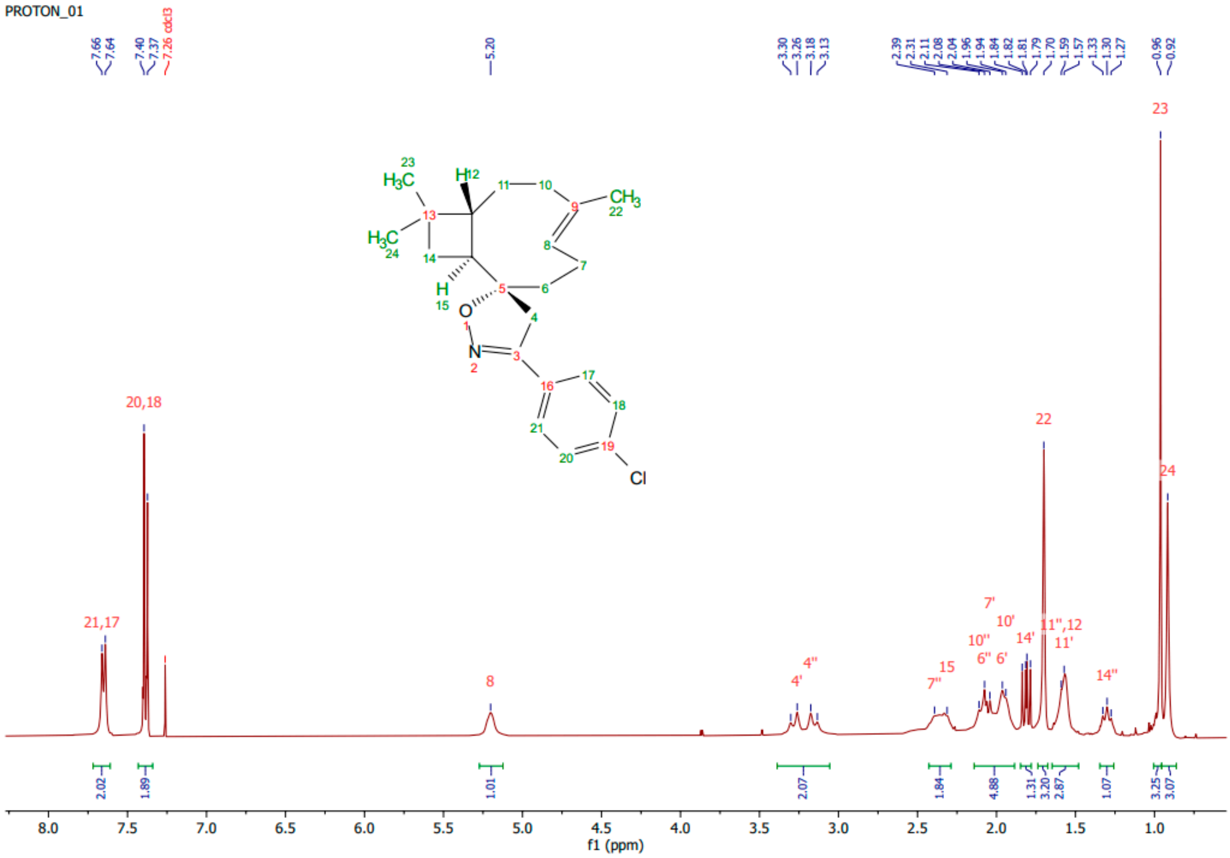

Figure S5. ^{1}H NMR spectra of compound **16a.**

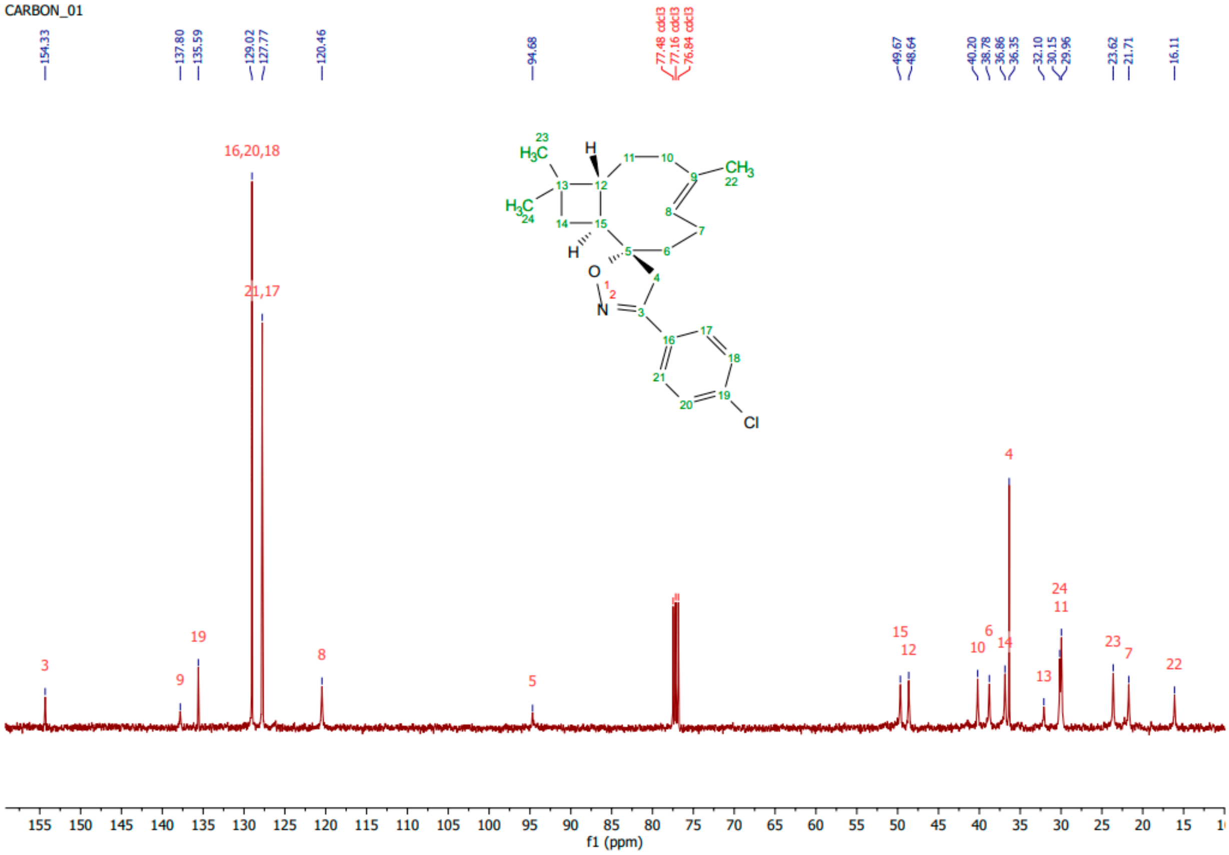

Figure S6. ^{13}C NMR spectra of compound **16a.**

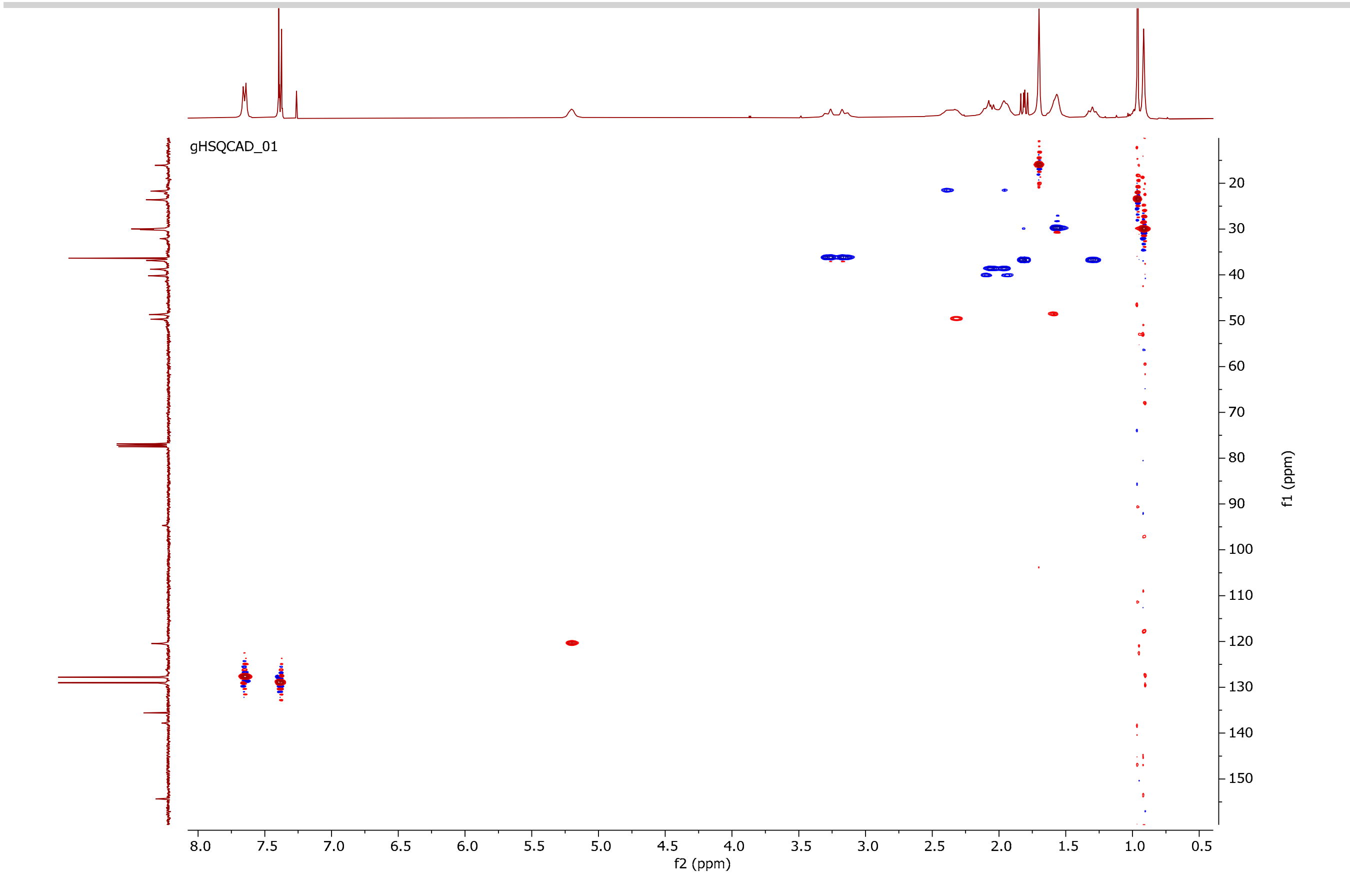

Figure S7. HSQC ^{1}H-^{13}C NMR spectra of compound **16a**.

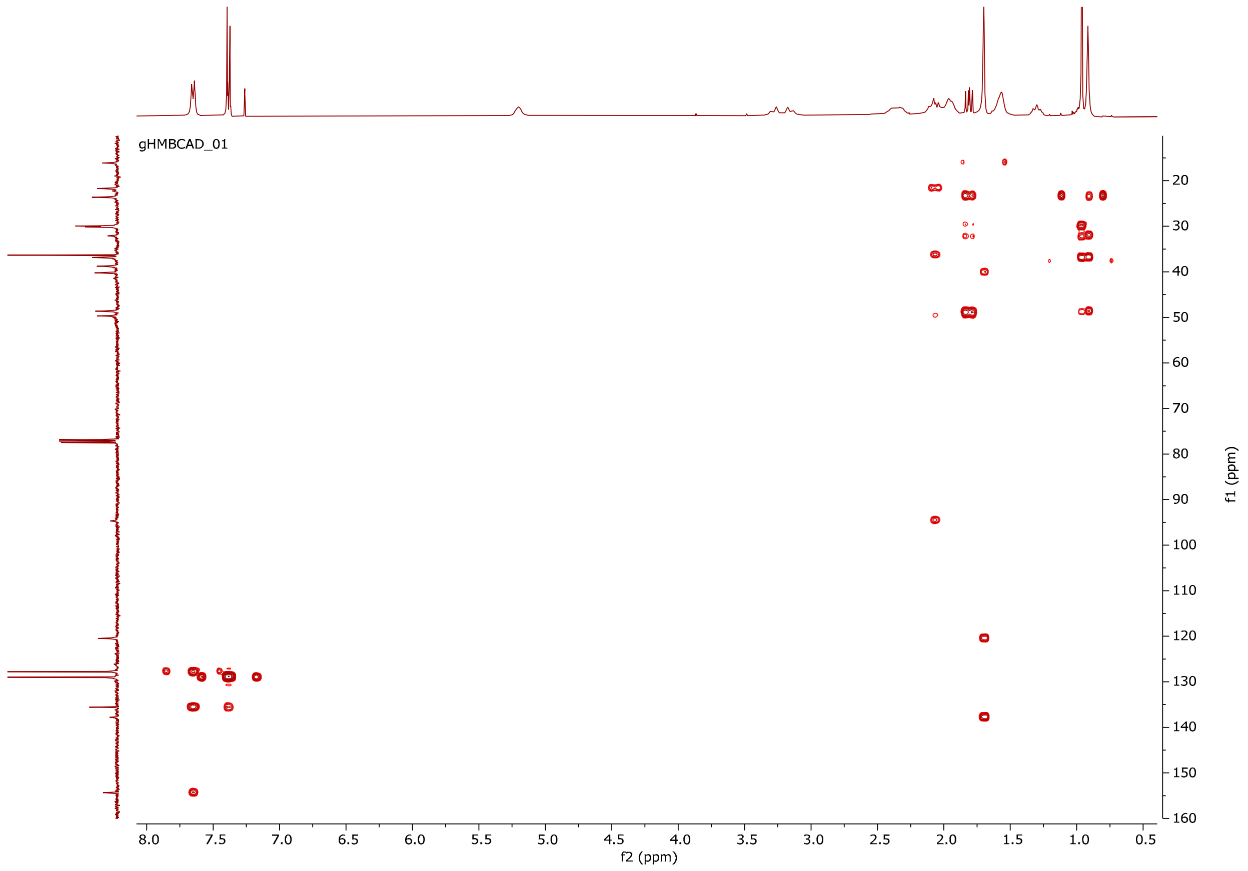

Figure S8. HMBC ^{1}H-^{13}C NMR spectra of compound **16a**.

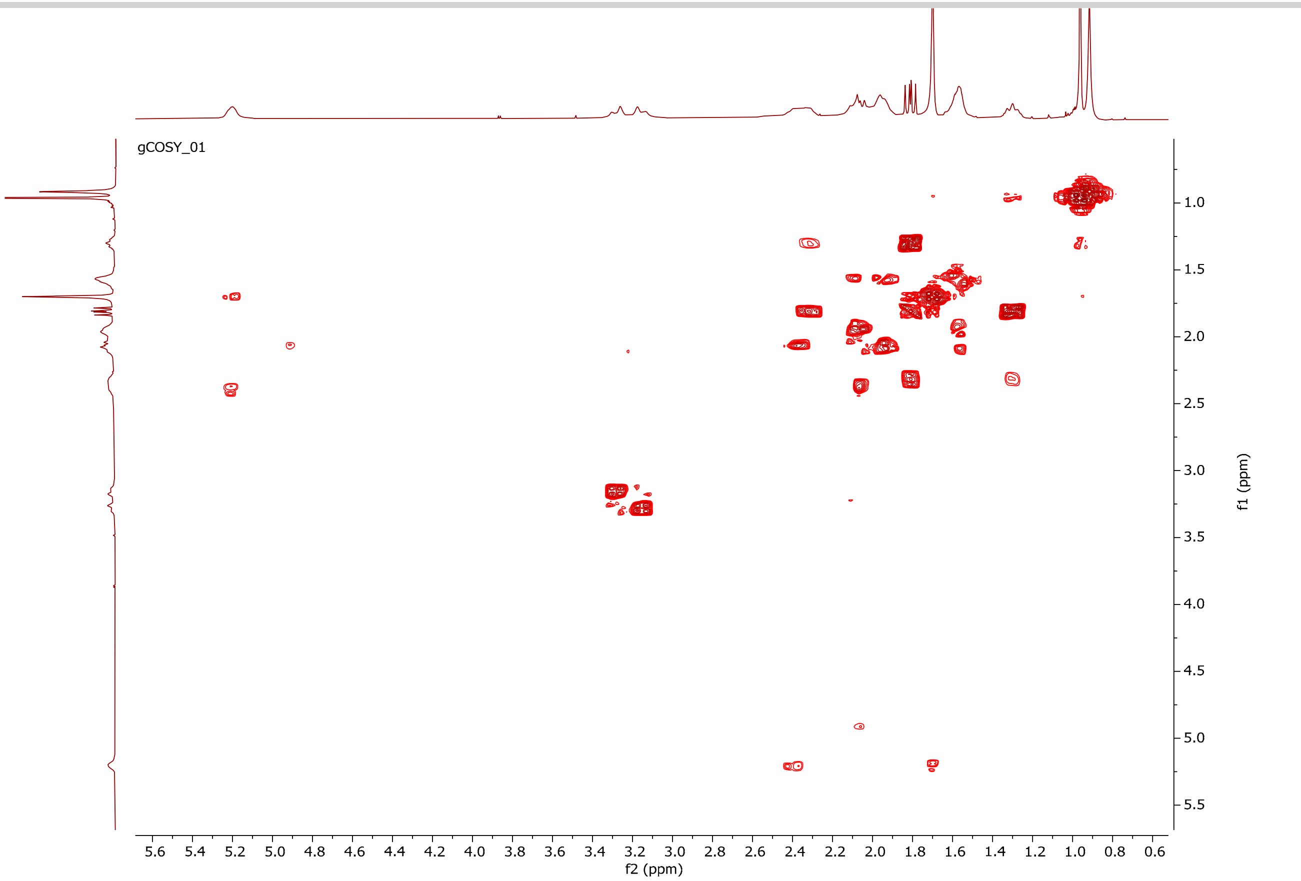

Figure S9. COSY ^1H-^1H NMR spectra of compound **16a**.

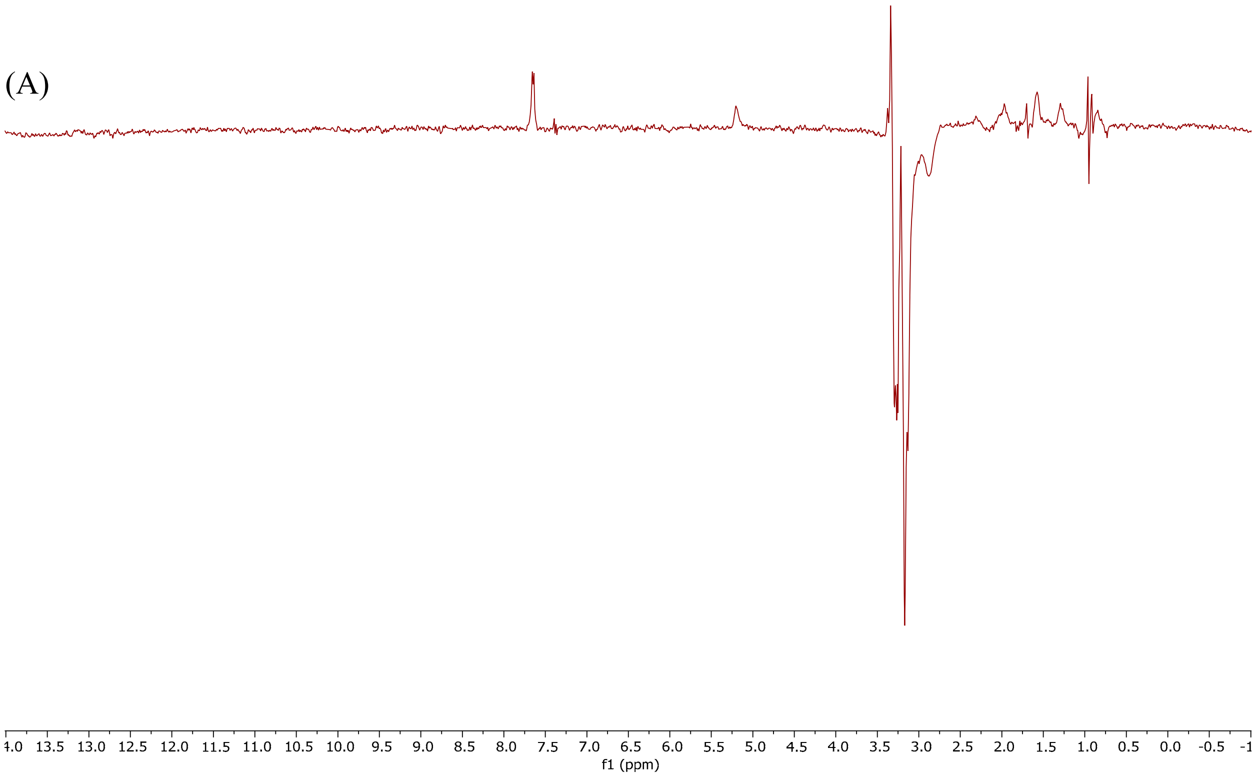

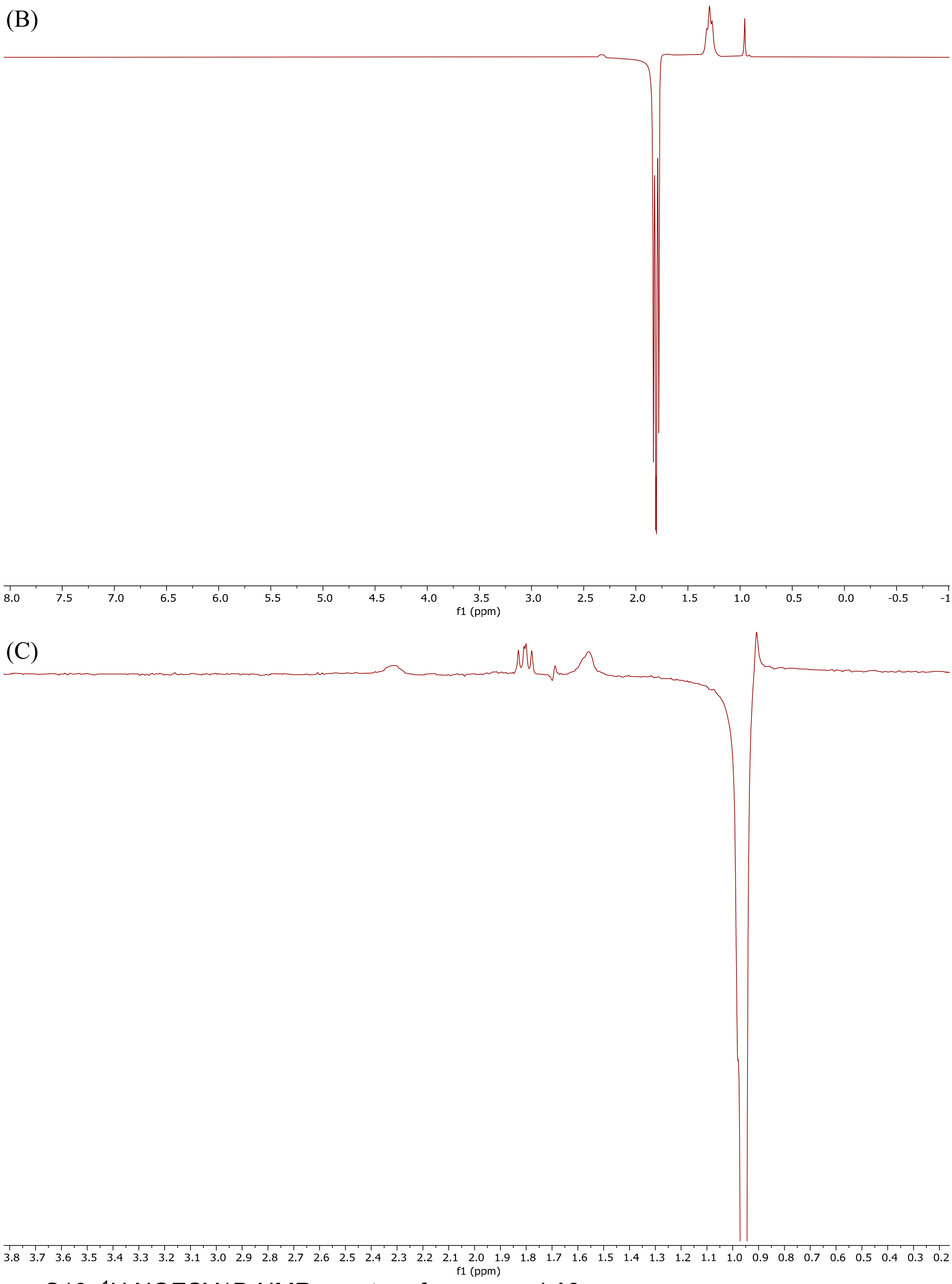

Figure S10. ^{1}H NOESY1D NMR spectra of compound **16a**.

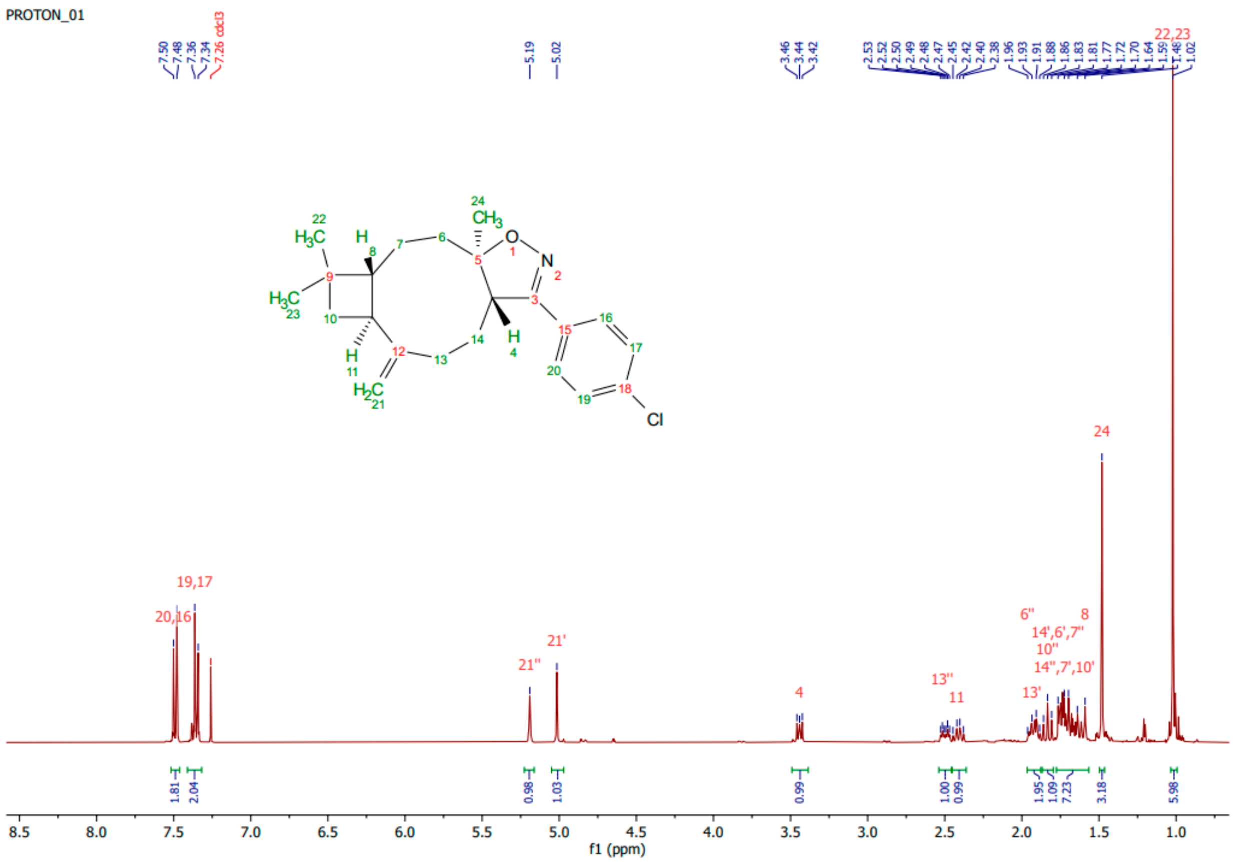

Figure S11. ^{1}H NMR spectra of compound **16b.**

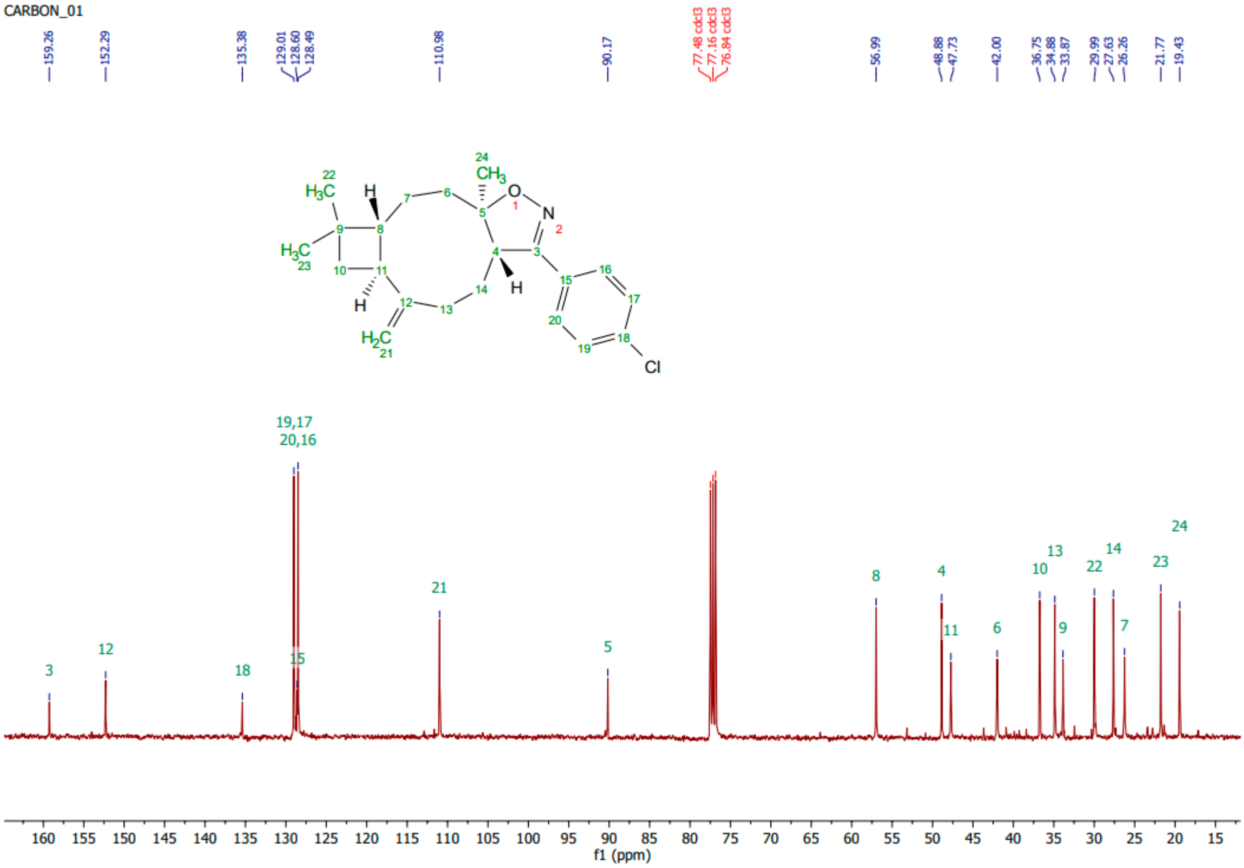

Figure S12. ^{13}C NMR spectra of compound **16b.**

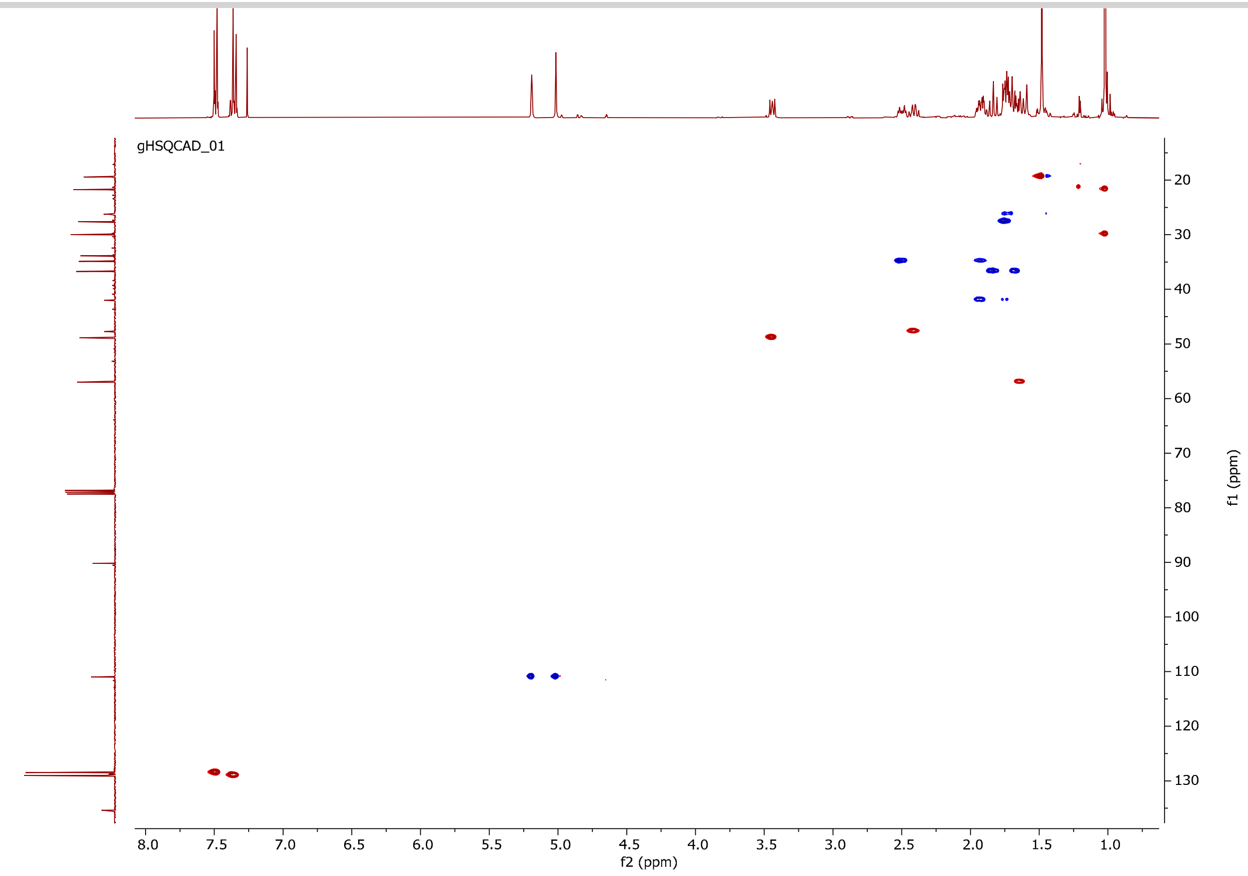

Figure S13. HSQC ^{1}H-^{13}C NMR spectra of compound **16b**.

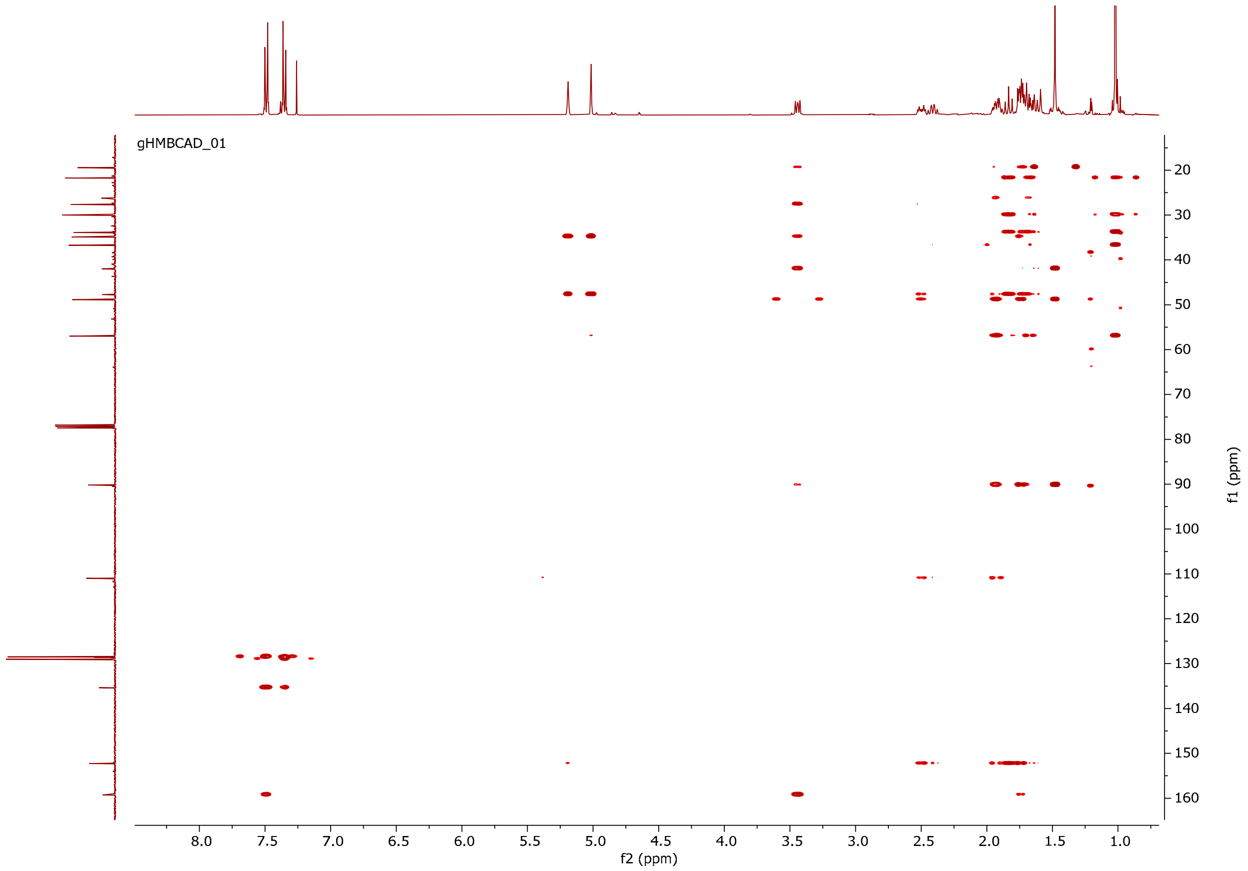

Figure S14. HMBC ^{1}H-^{13}C NMR spectra of compound **16b**.

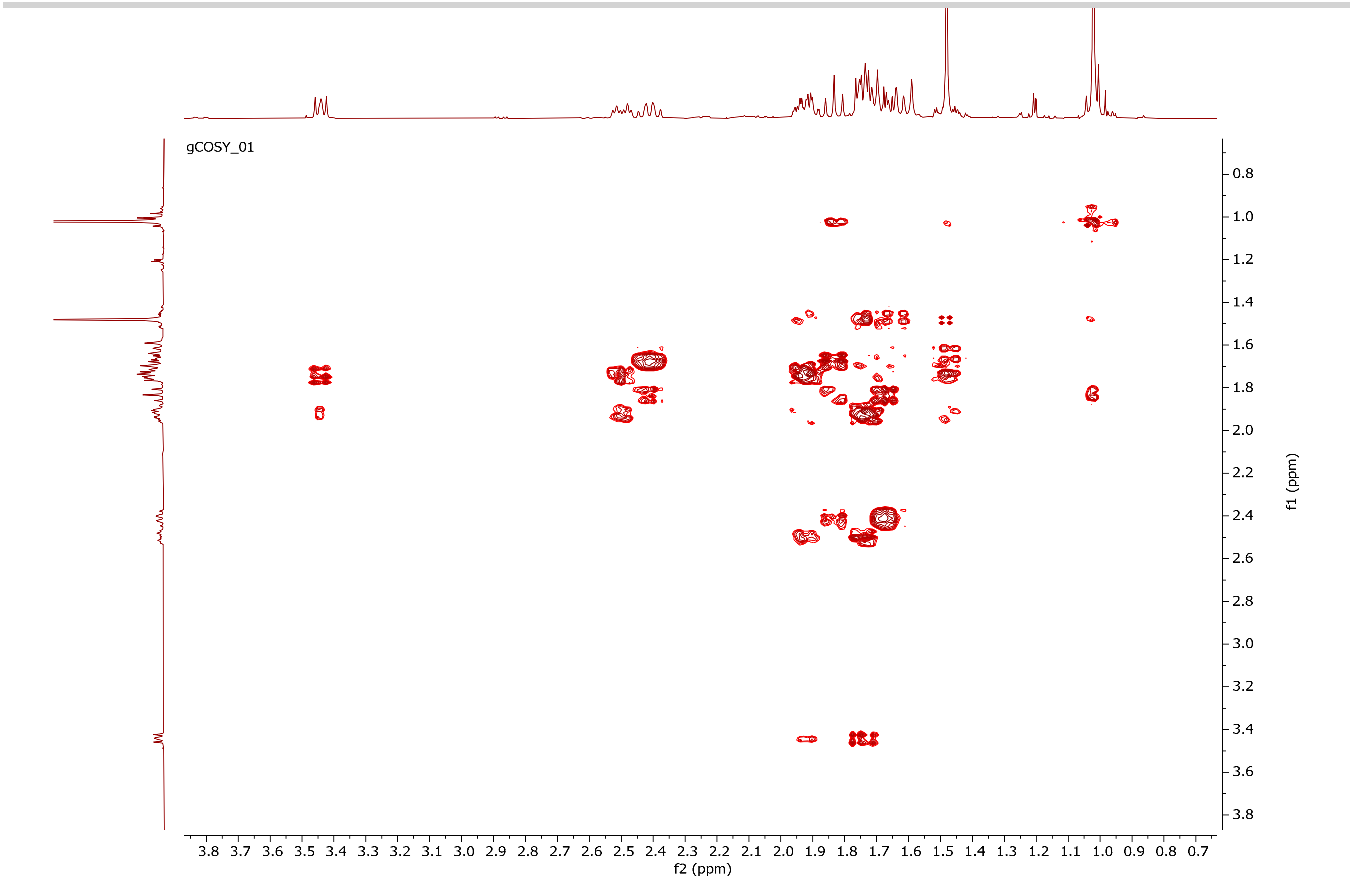

Figure S15. COESY ^{1}H-^{1}H NMR spectra of compound **16b**.

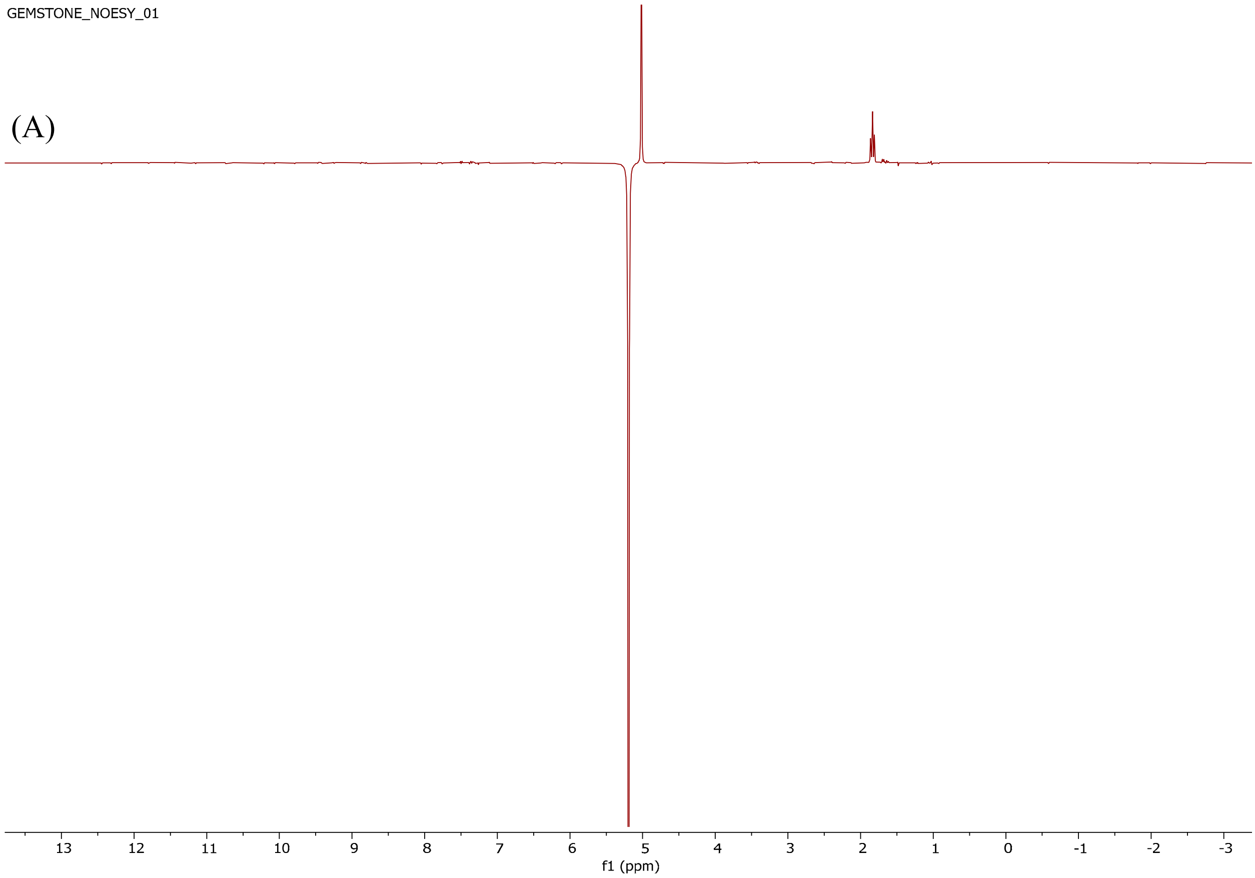

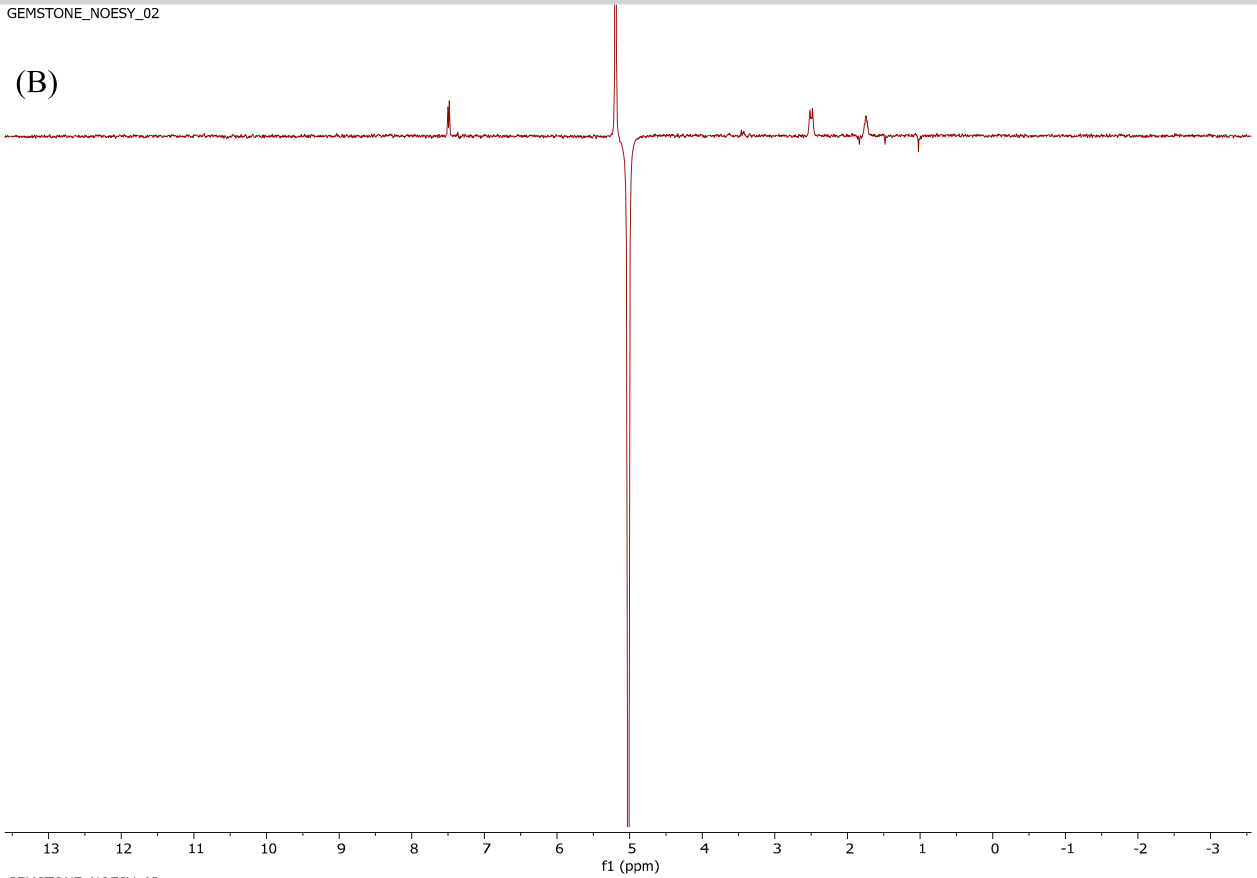
GEMSTONE_NOESY_02
(B)
13 12 11 10 9 8 7 6 5 4 3 2 1 0 -1 -2 -3
f1 (ppm)

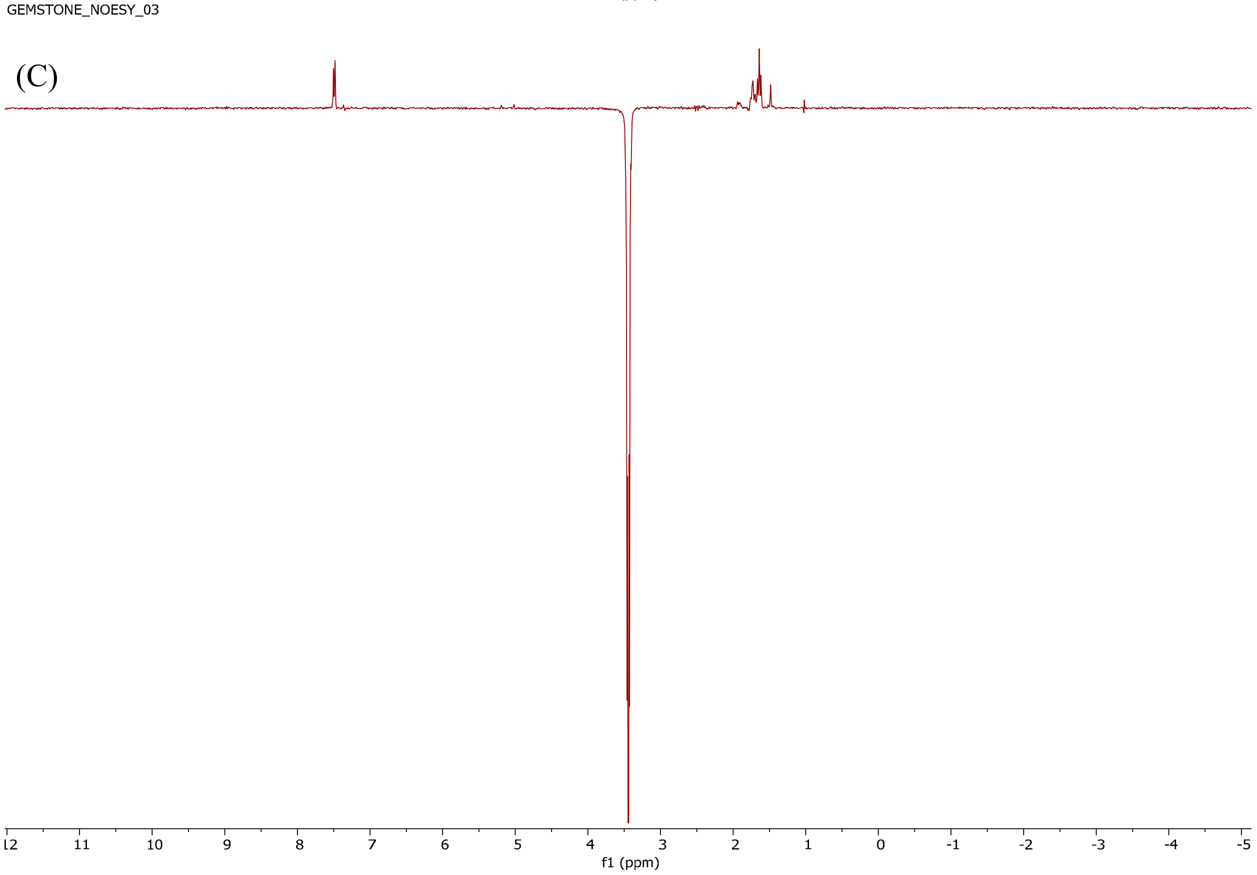
GEMSTONE_NOESY_03
(C)
11 10 9 8 7 6 5 4 3 2 1 0 -1 -2 -3 -4 -5
f1 (ppm)

GEMSTONE_NOESY_05

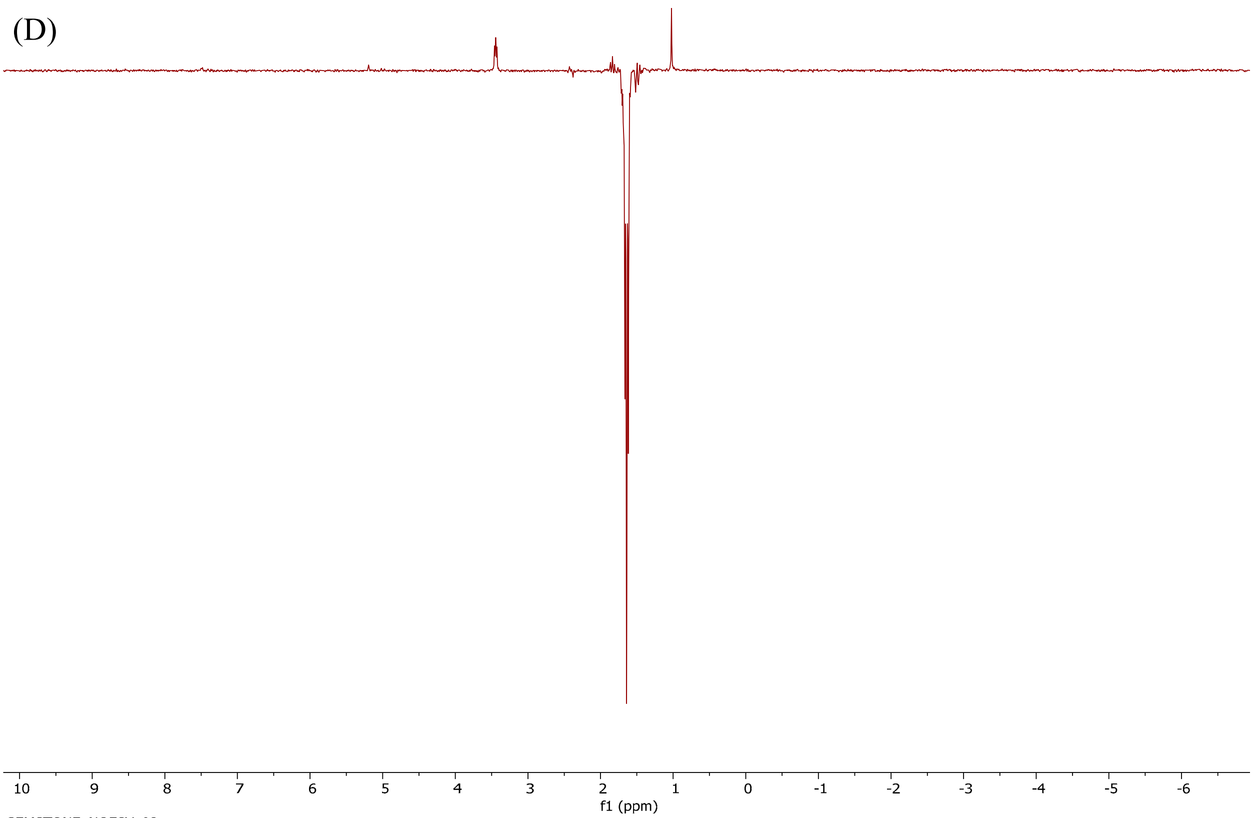

GEMSTONE_NOESY_08

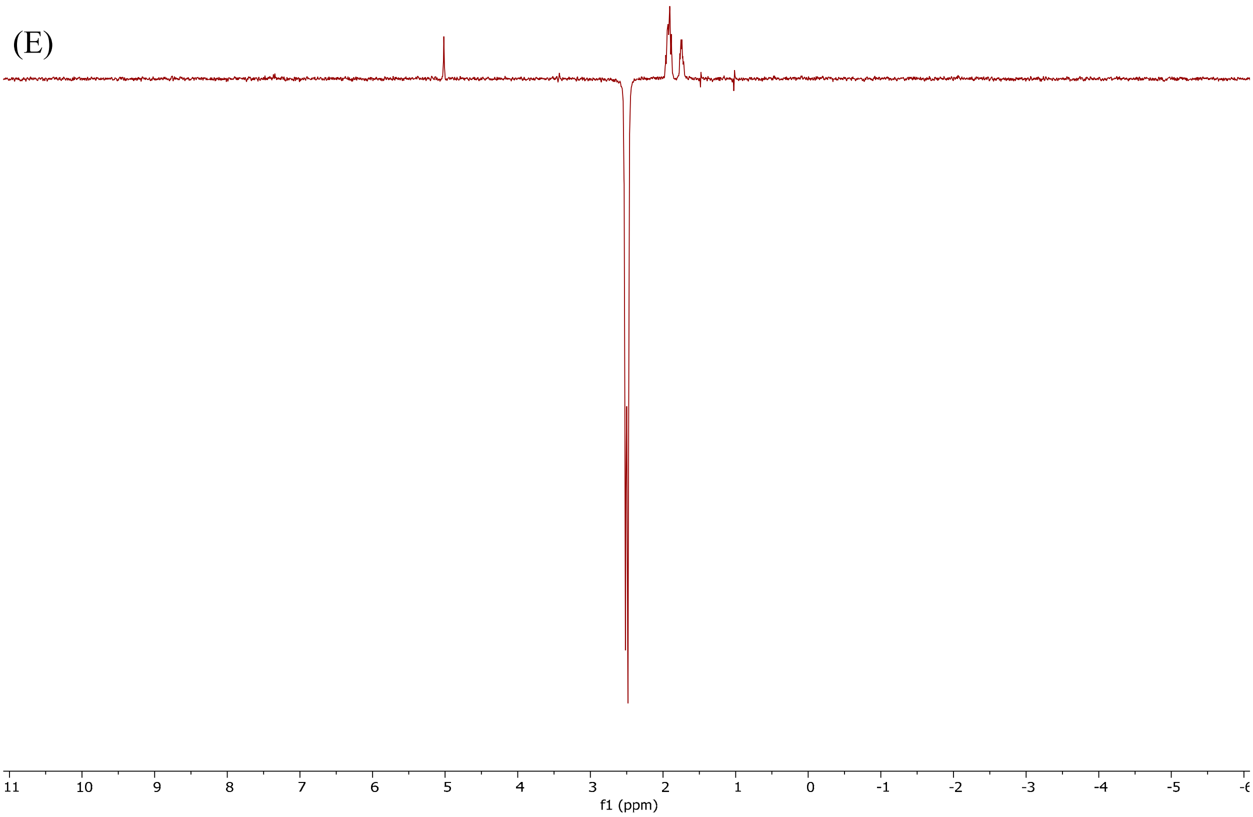

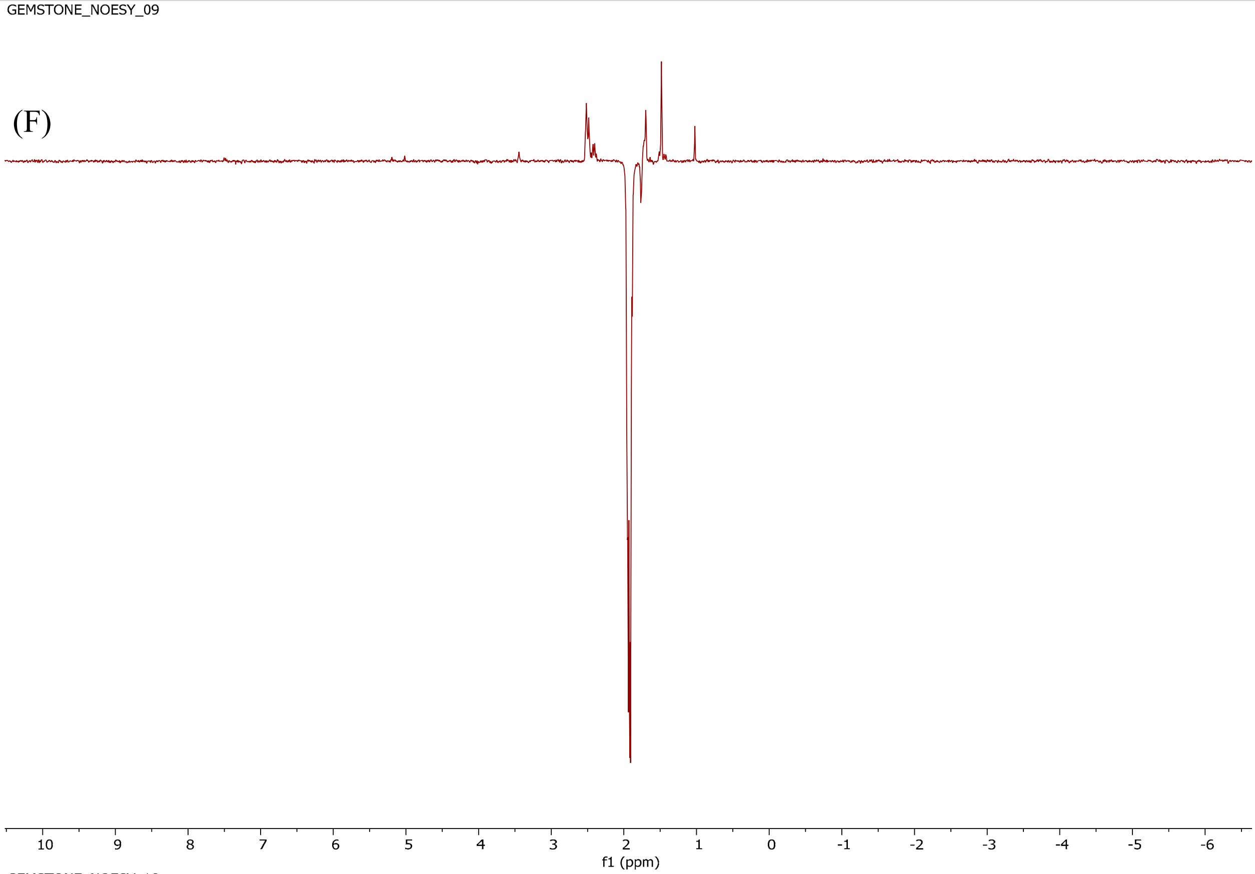

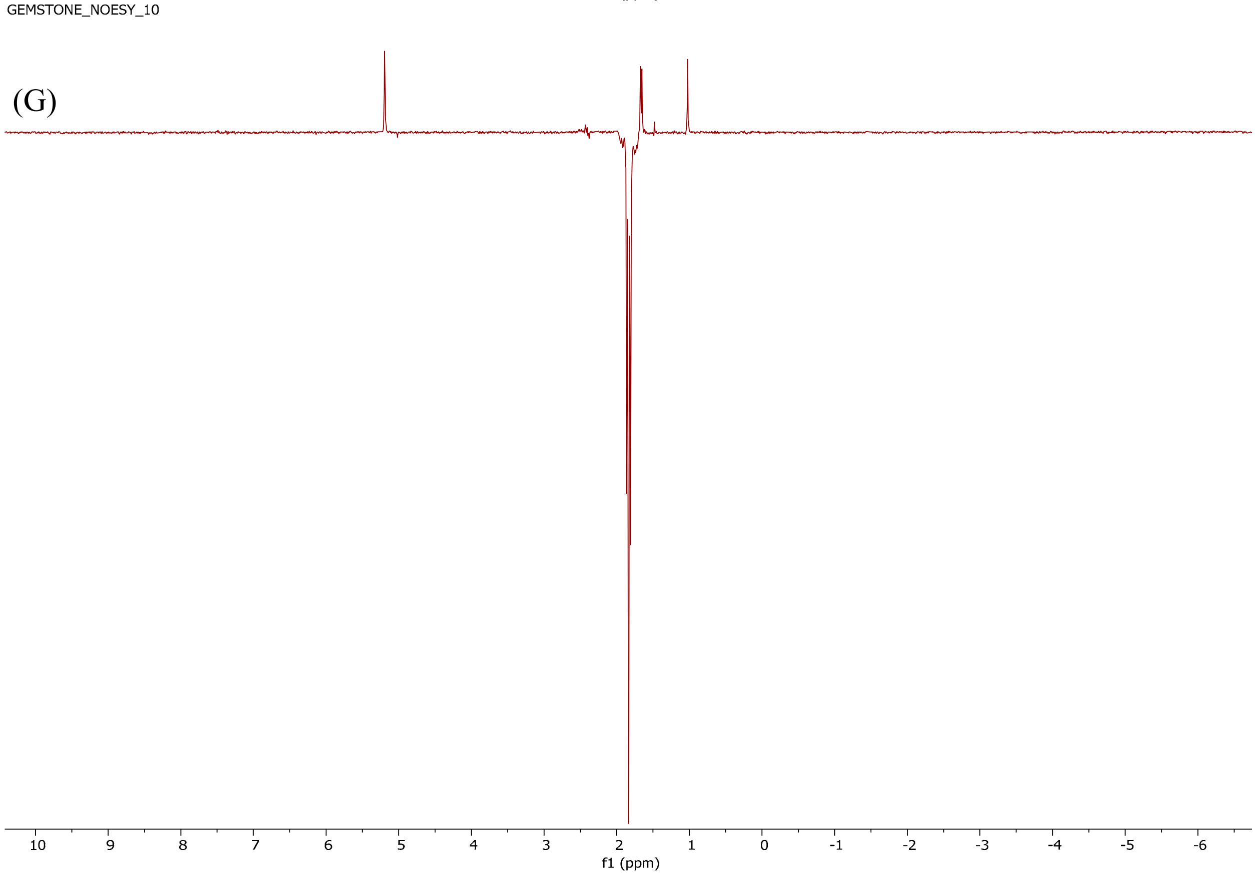

Figure S16. ^{1}H NOESY1D NMR spectra of compound **16b.**

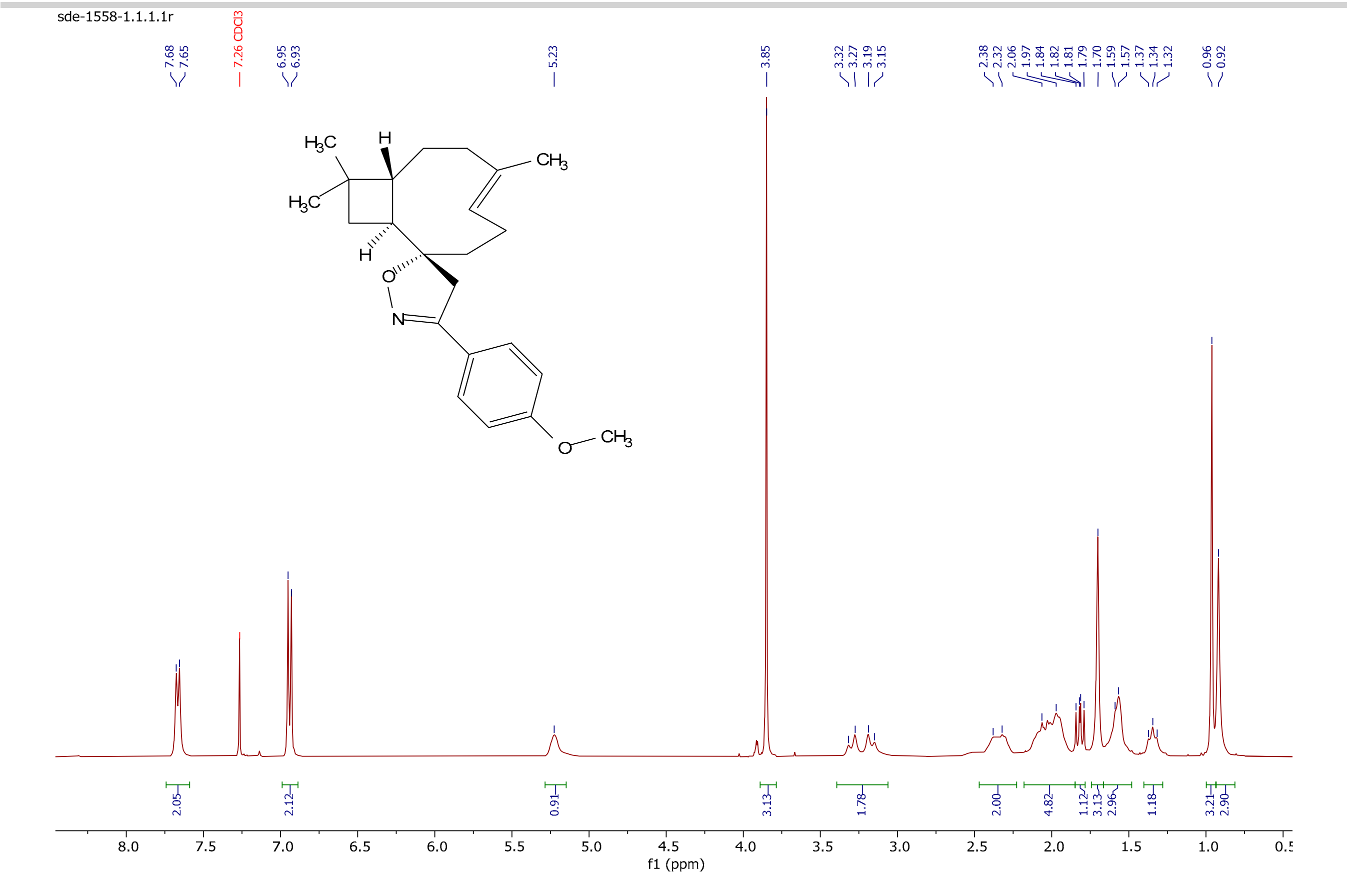

Figure S17. ^{1}H NMR spectra of compound **17a.**

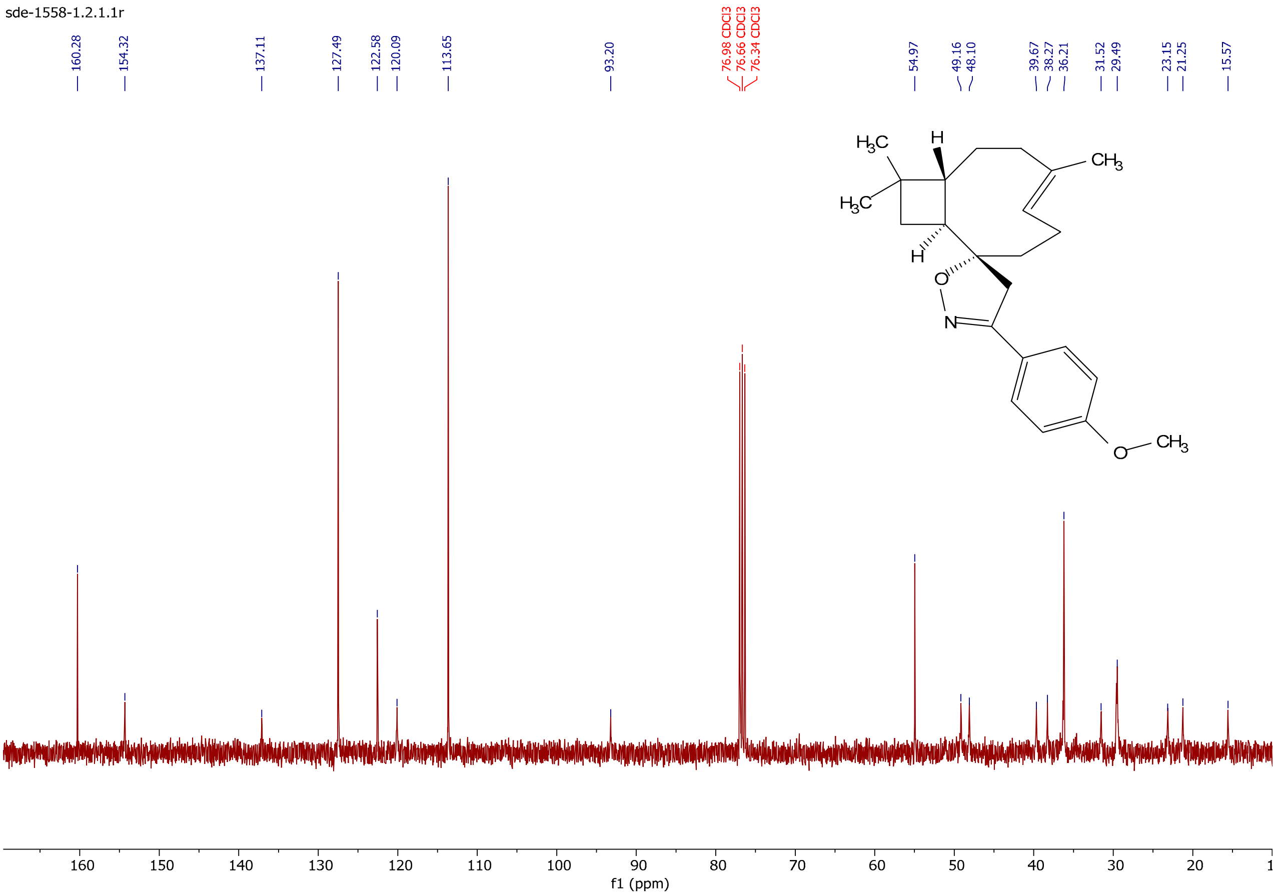

Figure S18. ^{13}C NMR spectra of compound **17a.**

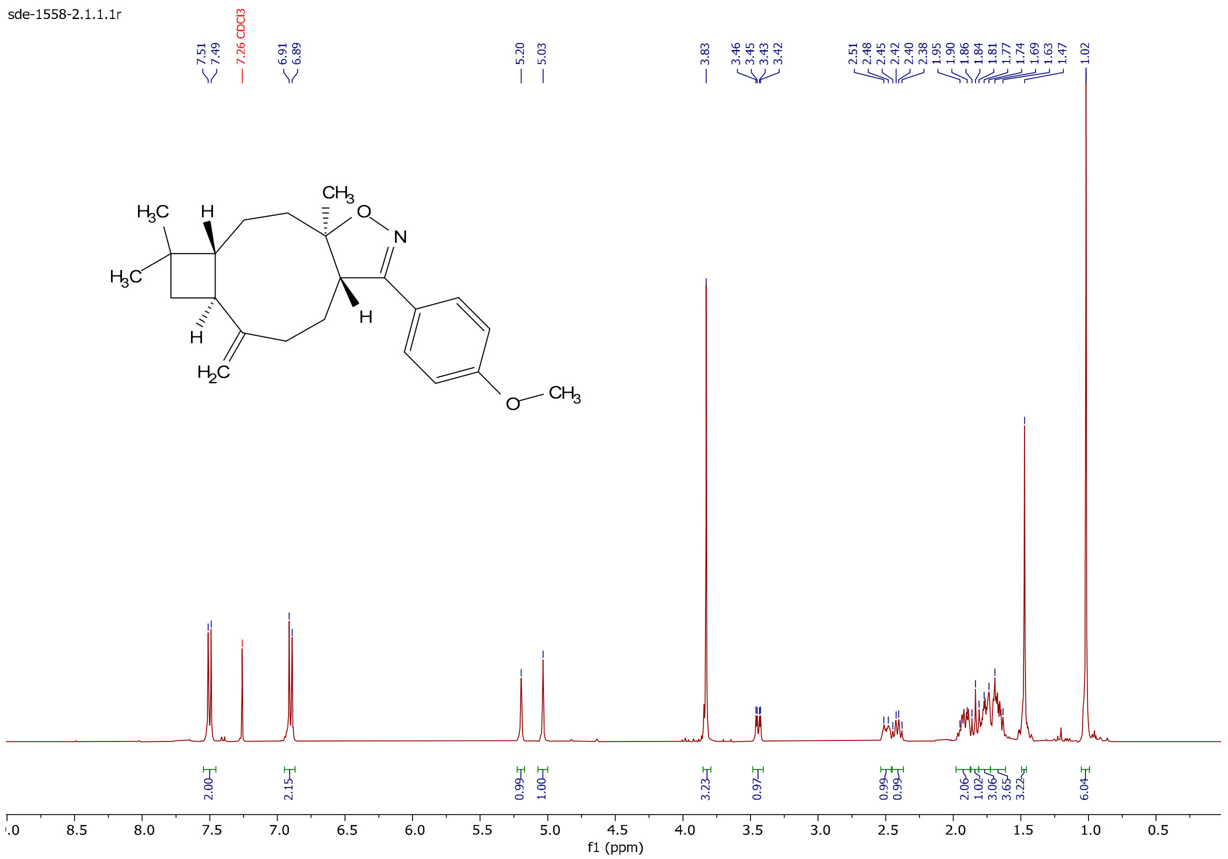

Figure S19. ^{1}H NMR spectra of compound **17b.**

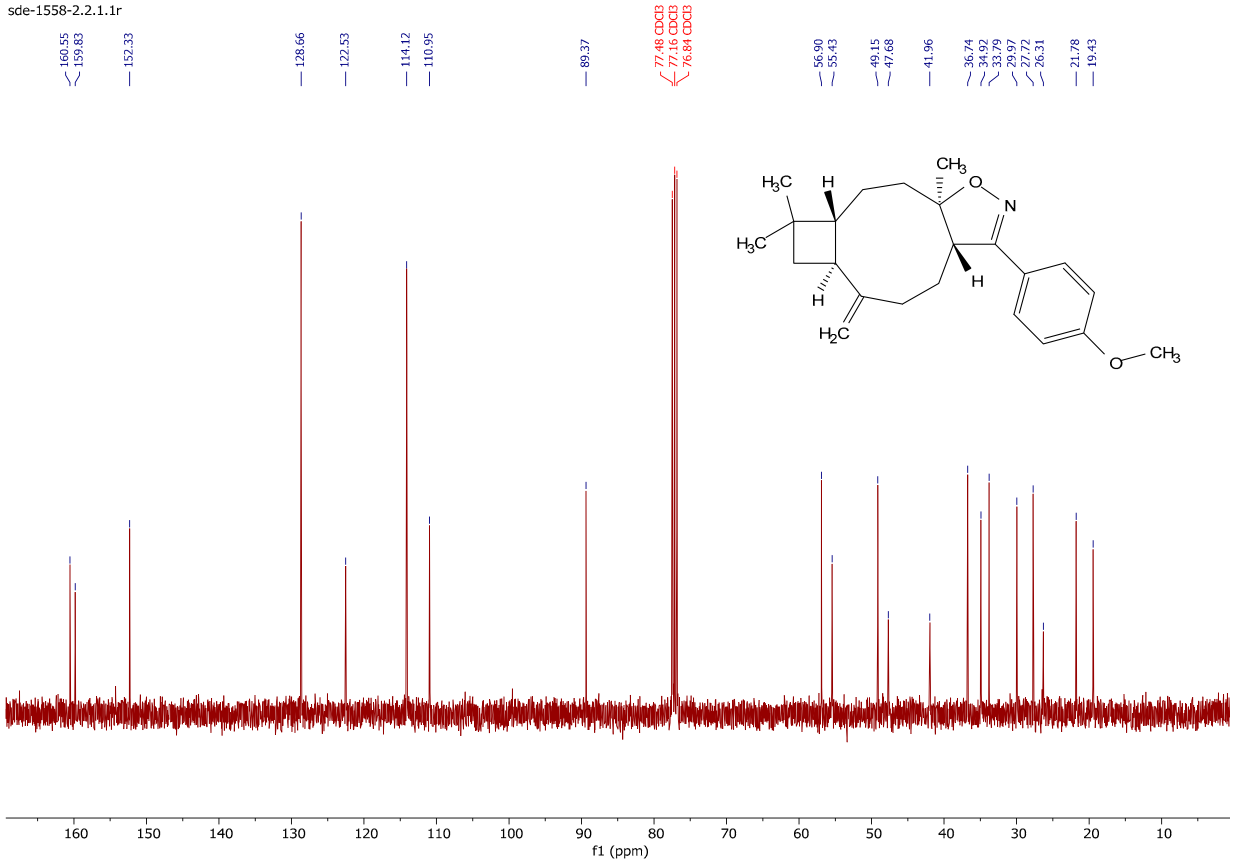

Figure S20. ^{13}C NMR spectra of compound **17b.**

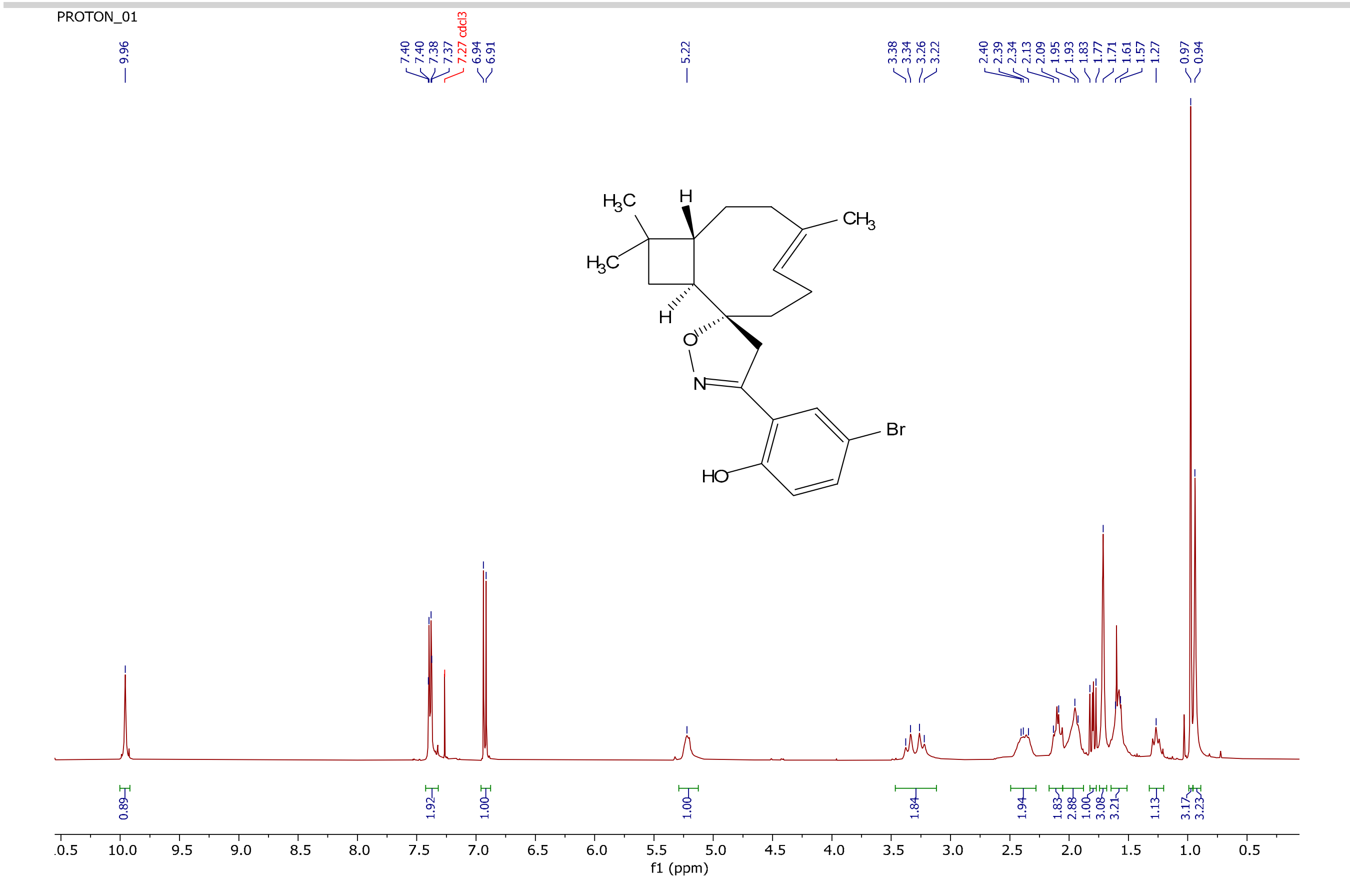

Figure S21. ^{1}H NMR spectra of compound **18a.**

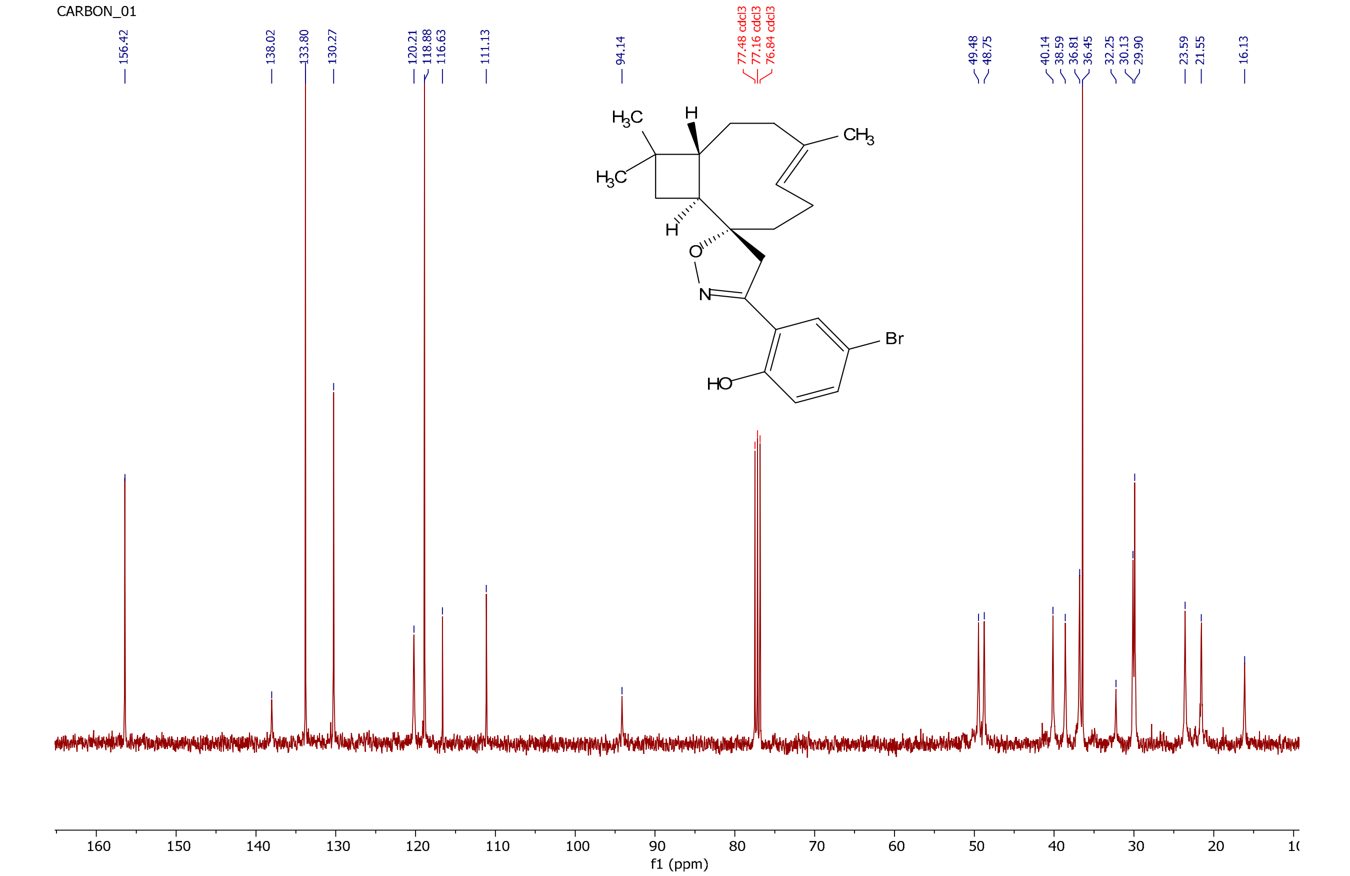

Figure S22. ^{13}C NMR spectra of compound **18a.**

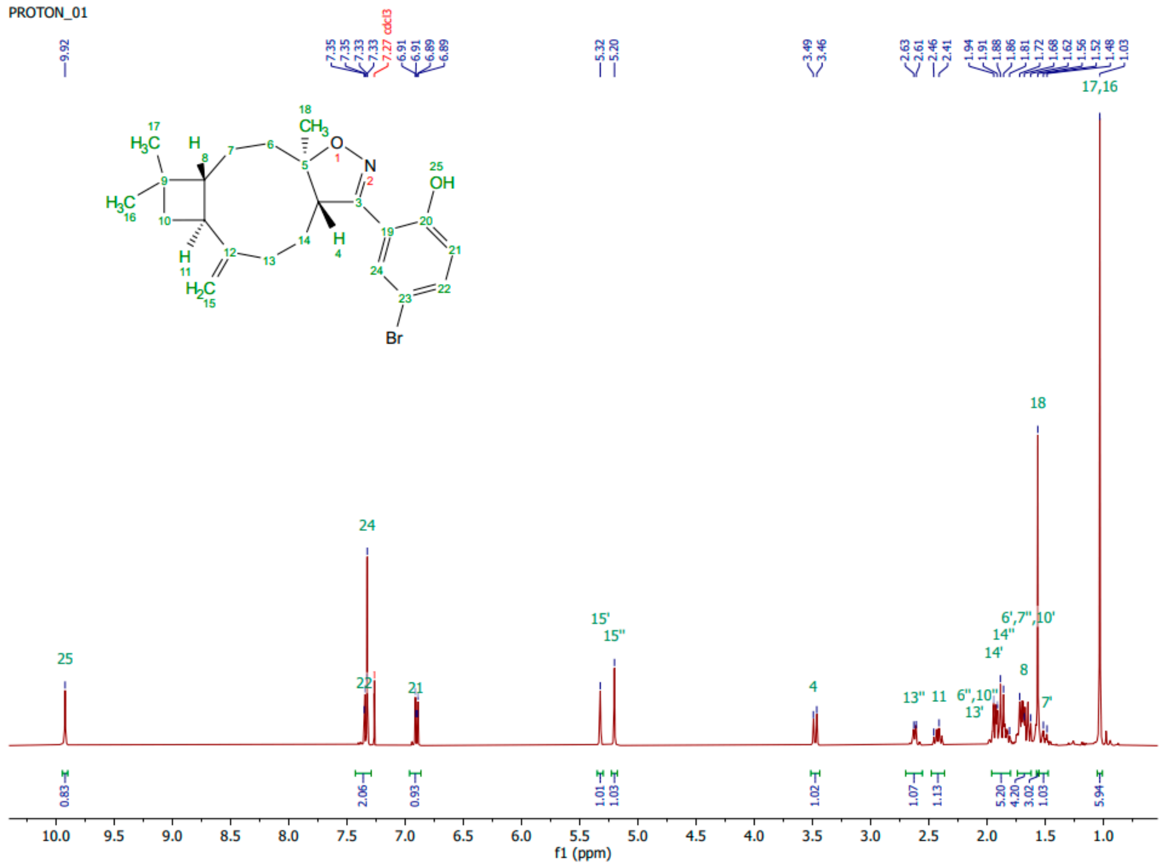

Figure S23. ¹H NMR spectra of compound **18b.**

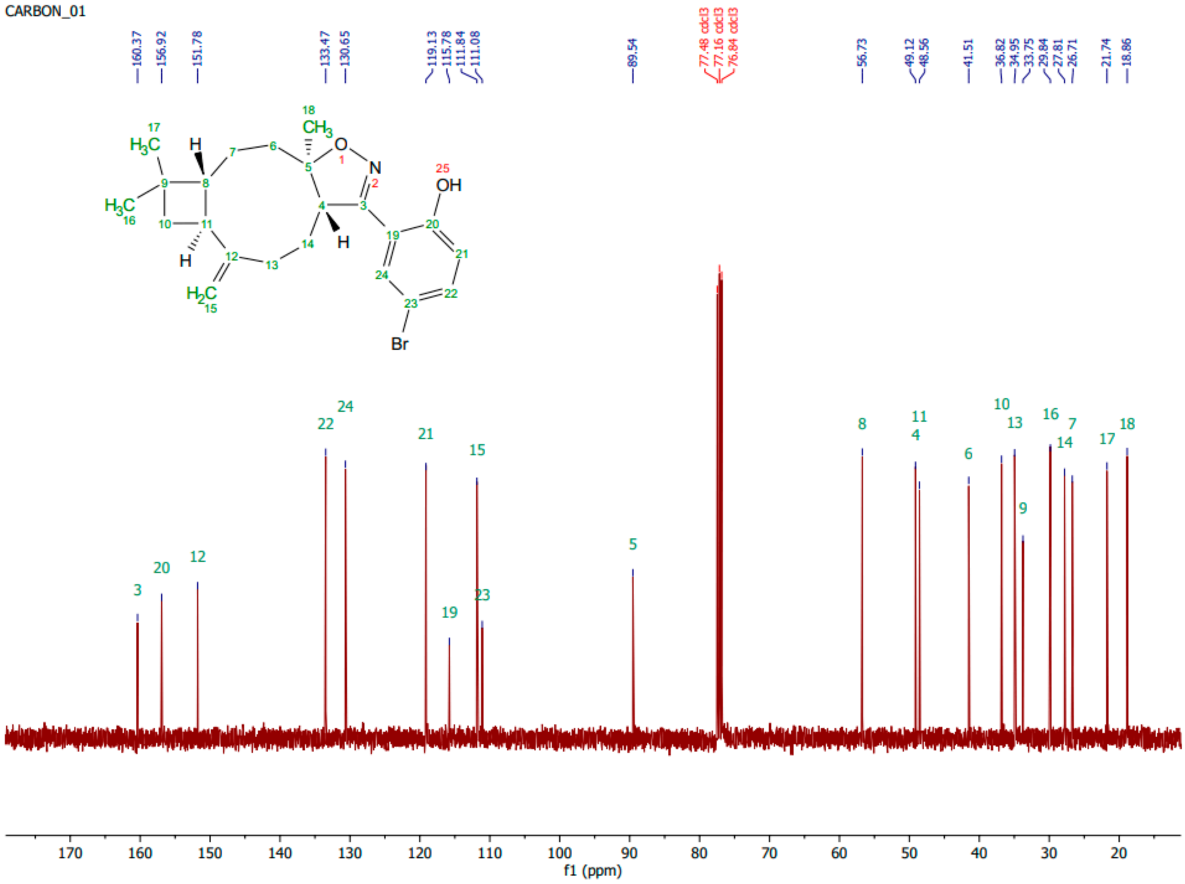

Figure S24. ¹³C NMR spectra of compound **18b.**

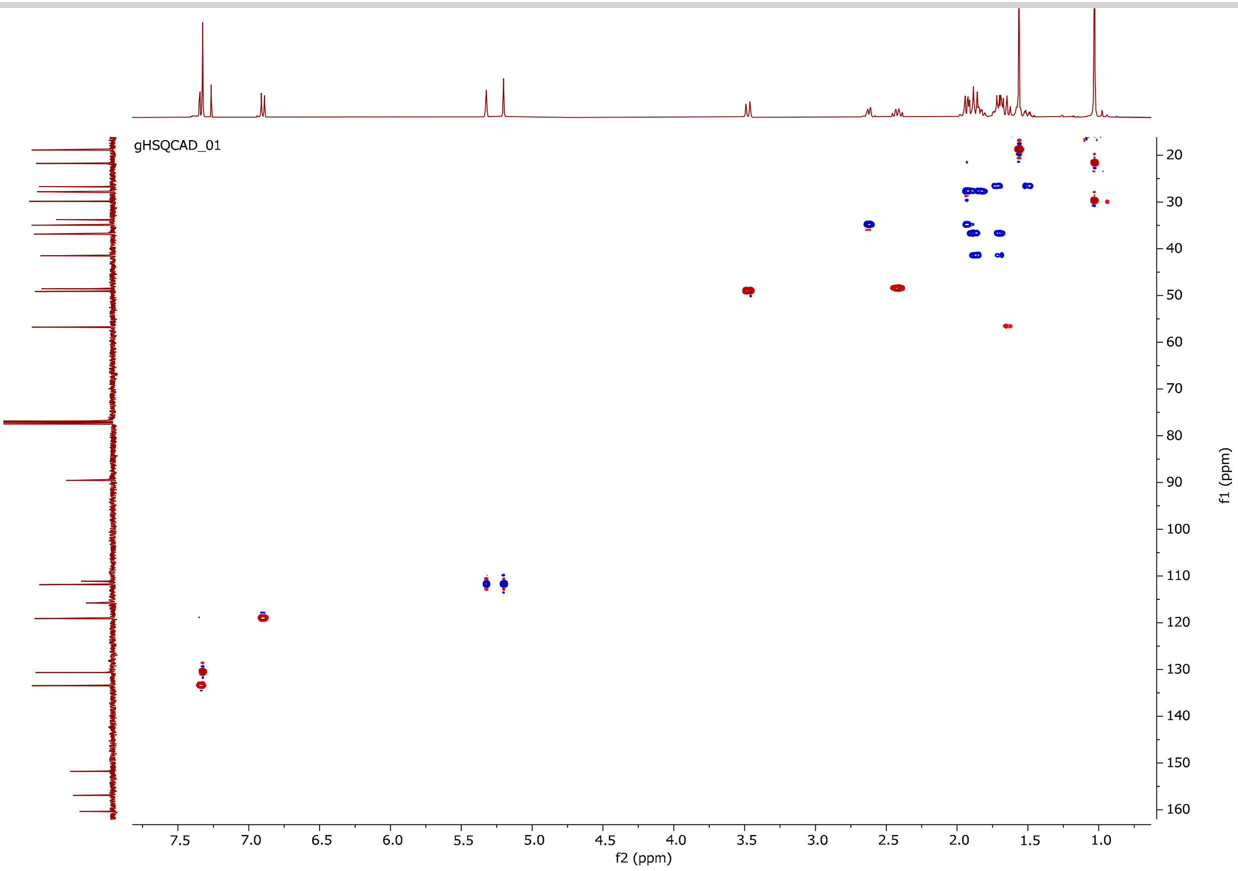

Figure S25. HSQC ^{1}H-^{13}C NMR spectra of compound **18b**.

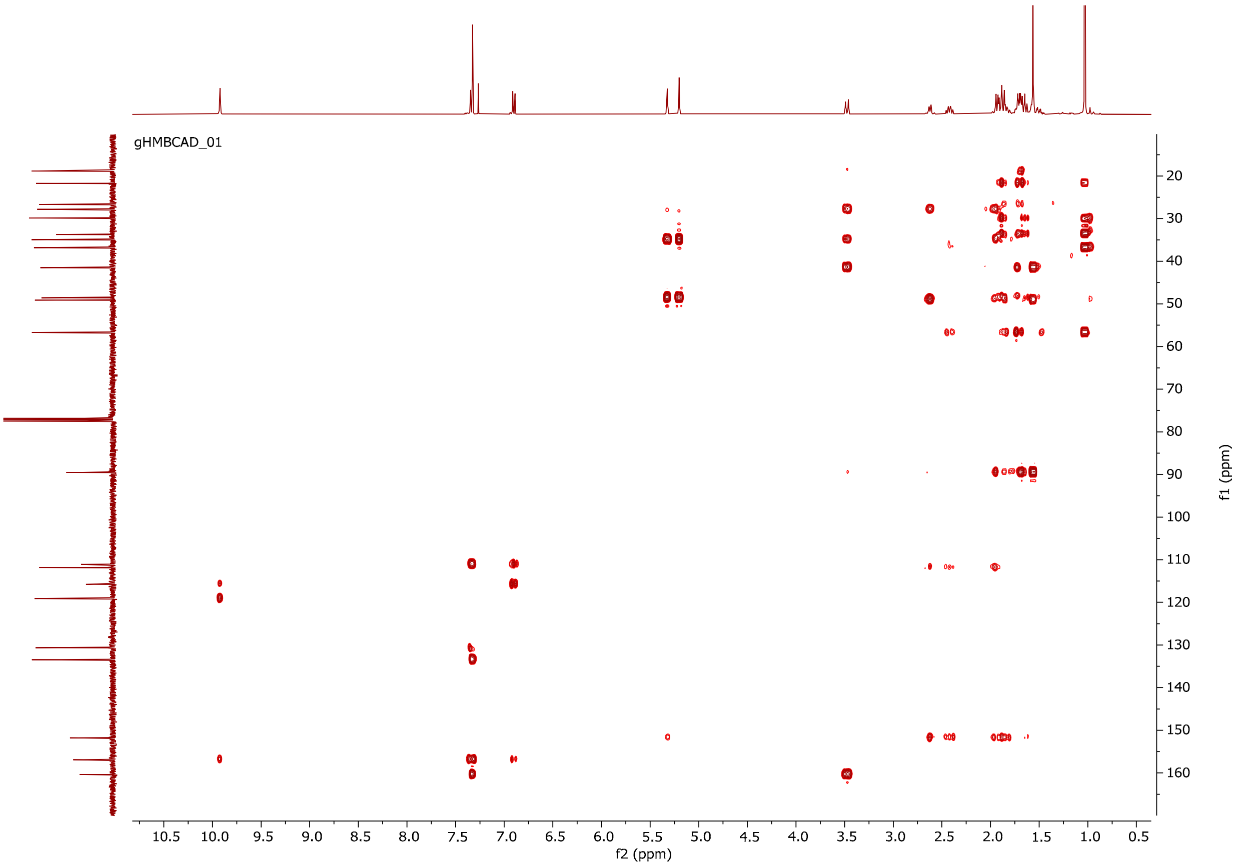

Figure S26. HMBC ^{1}H-^{13}C NMR spectra of compound **18b**.

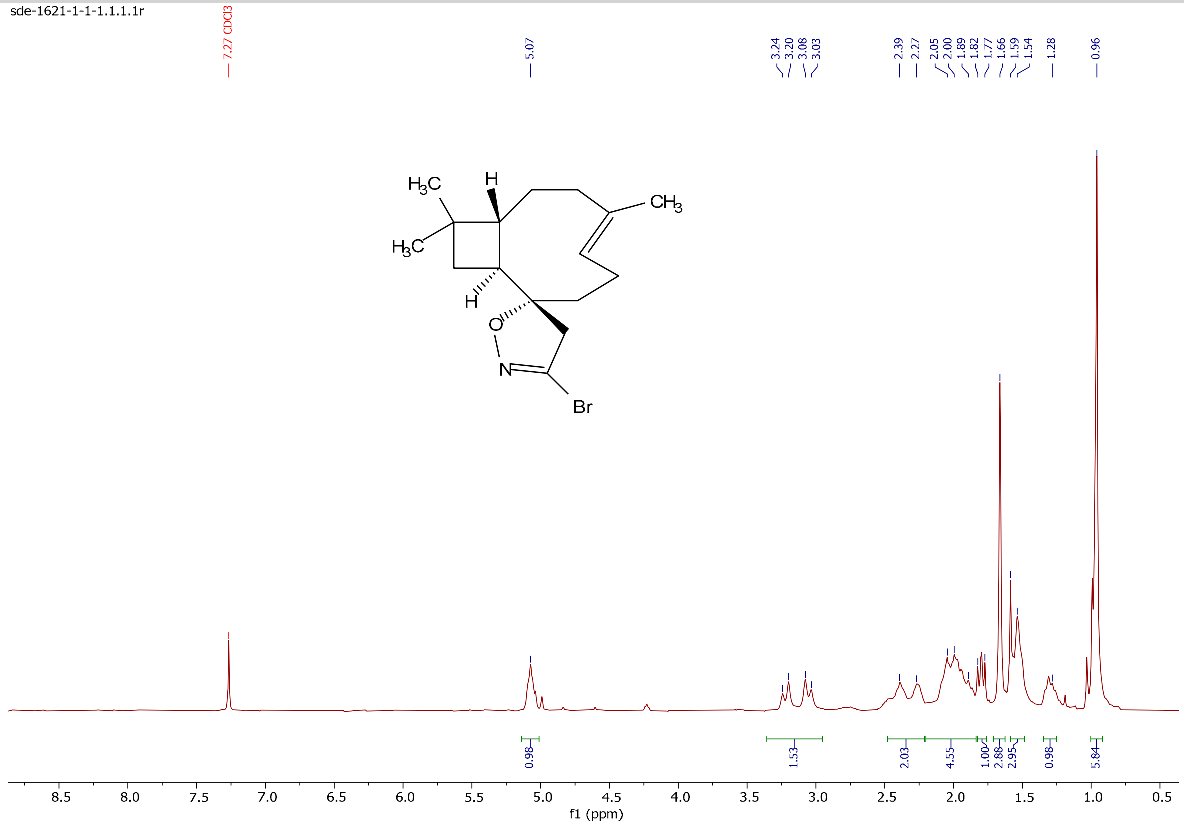

Figure S27. ^{1}H NMR spectra of compound **19a.**

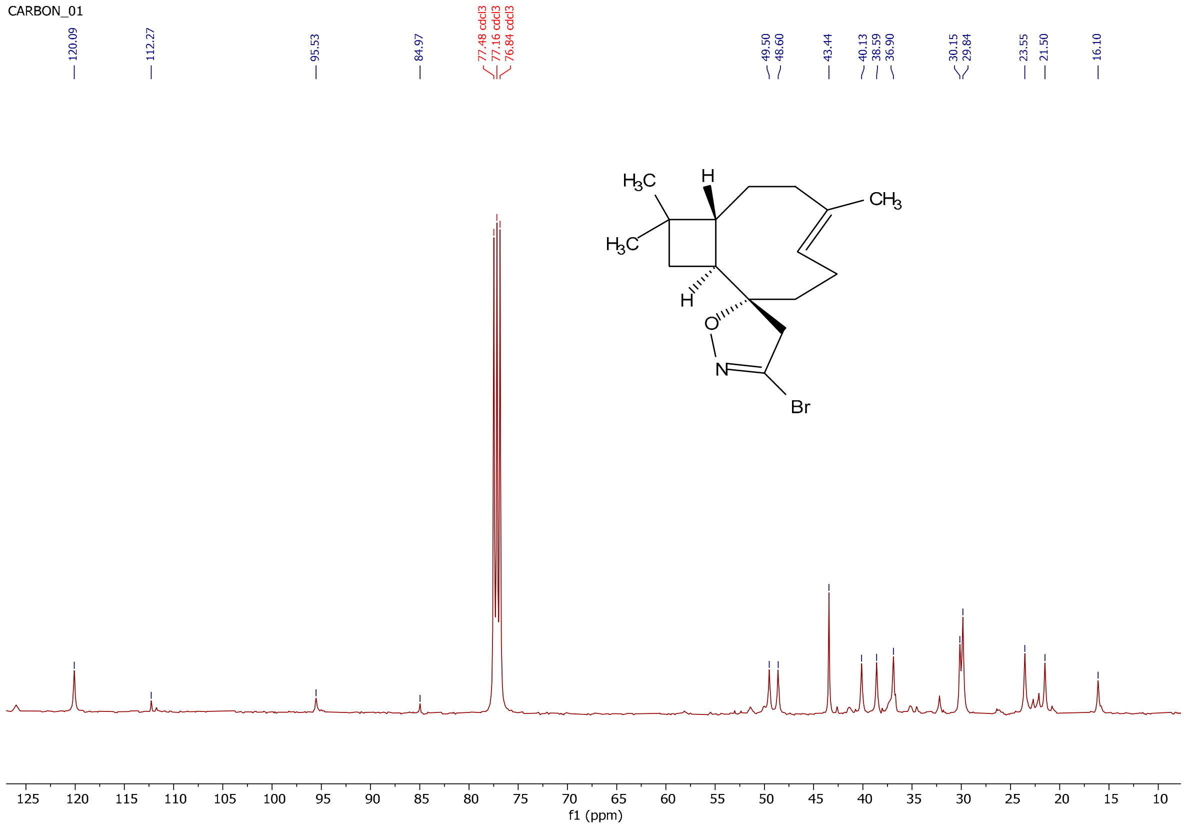

Figure S28. ^{13}C NMR spectra of compound **19a.**

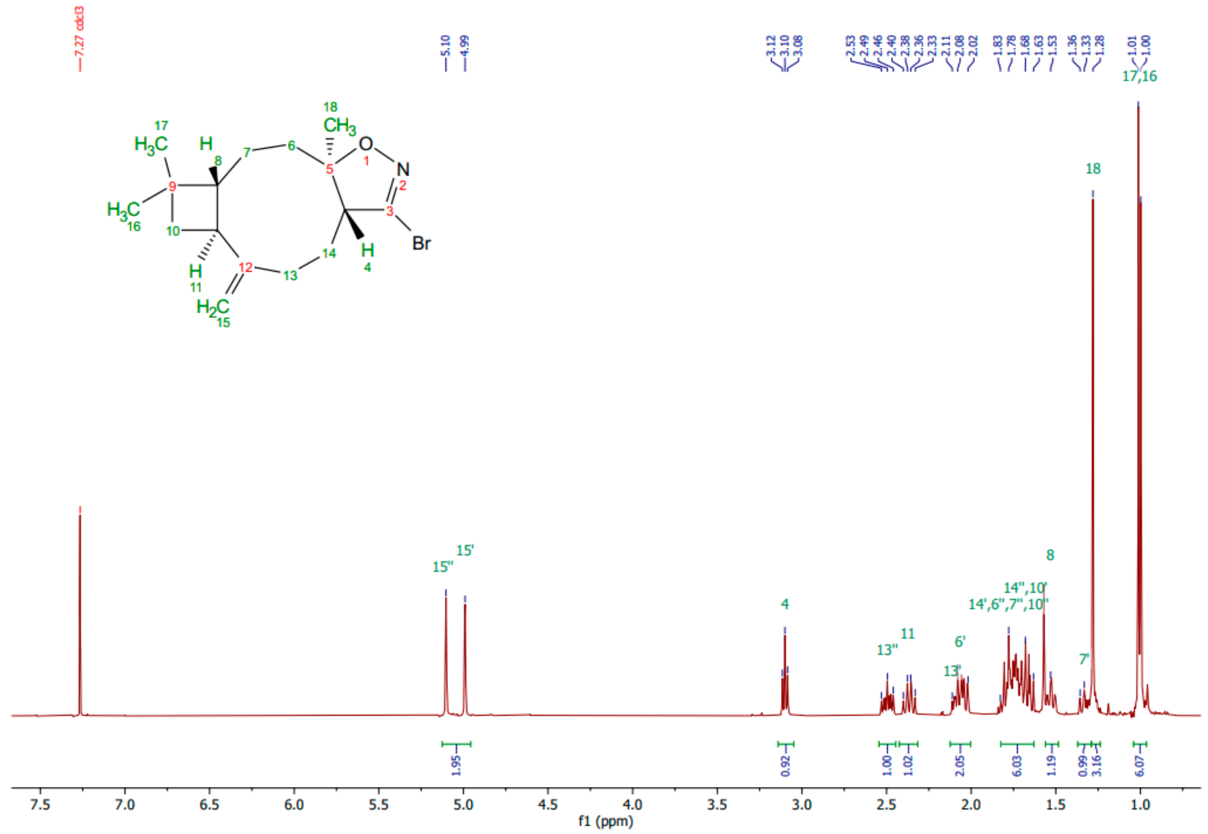

Figure S29. ^{1}H NMR spectra of compound **19b.**

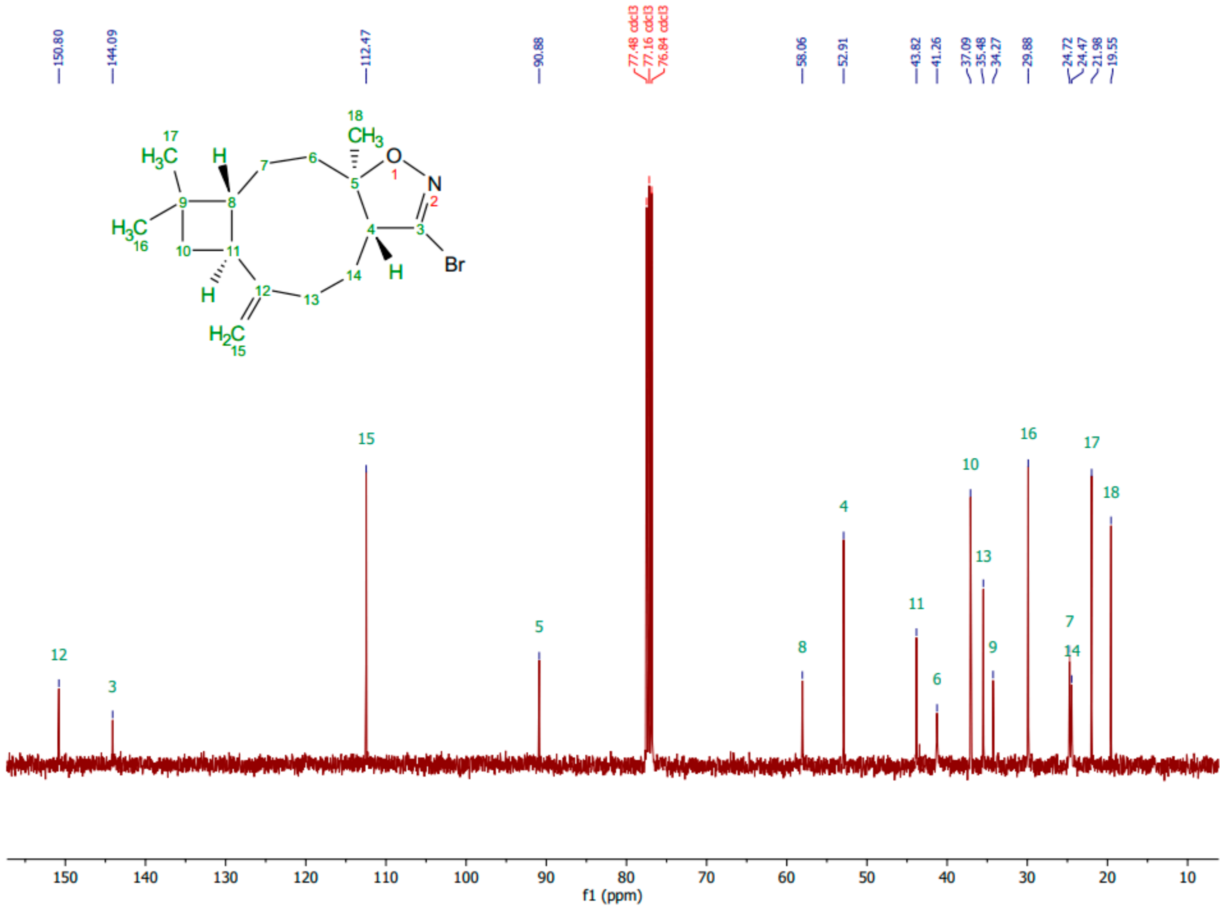

Figure S30. ^{13}C NMR spectra of compound **19b.**

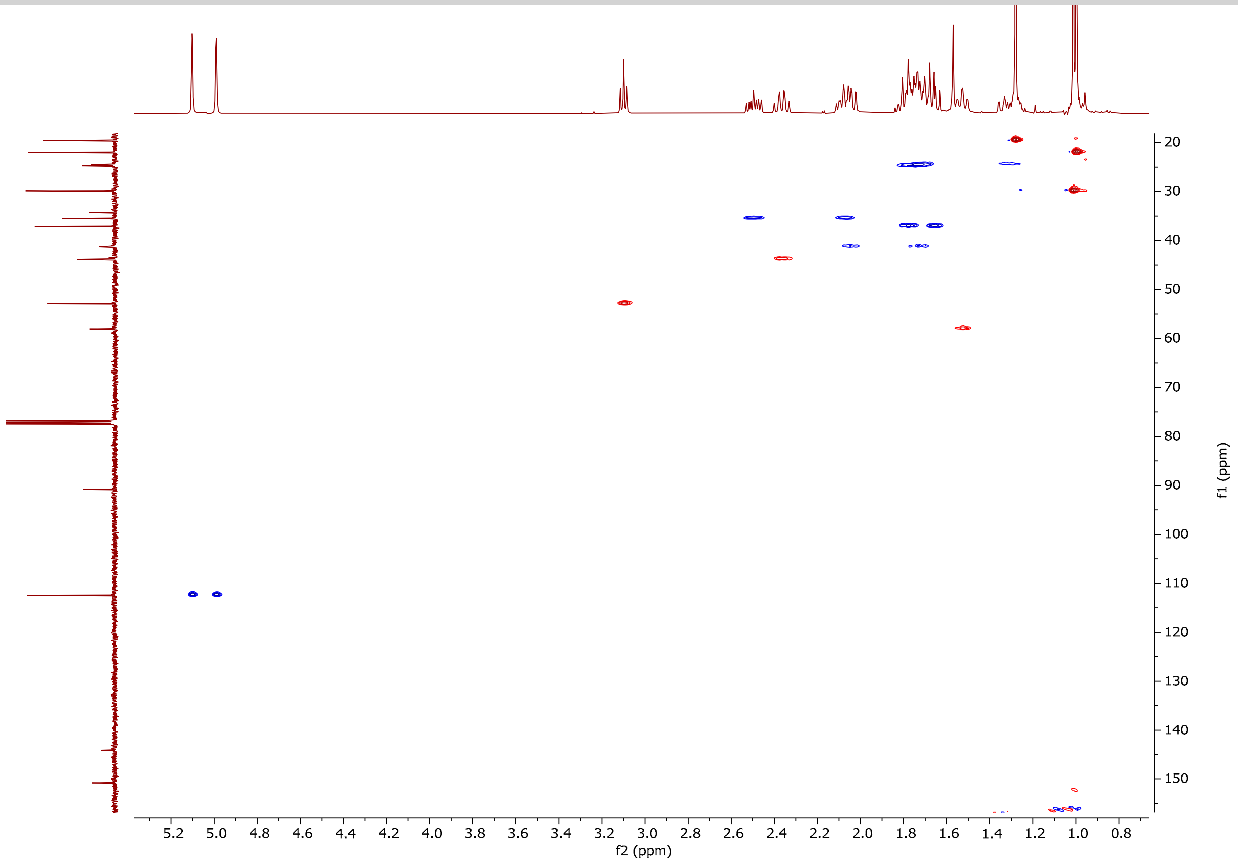

Figure S31. HSQC ^{1}H-^{13}C NMR spectra of compound **19b**.

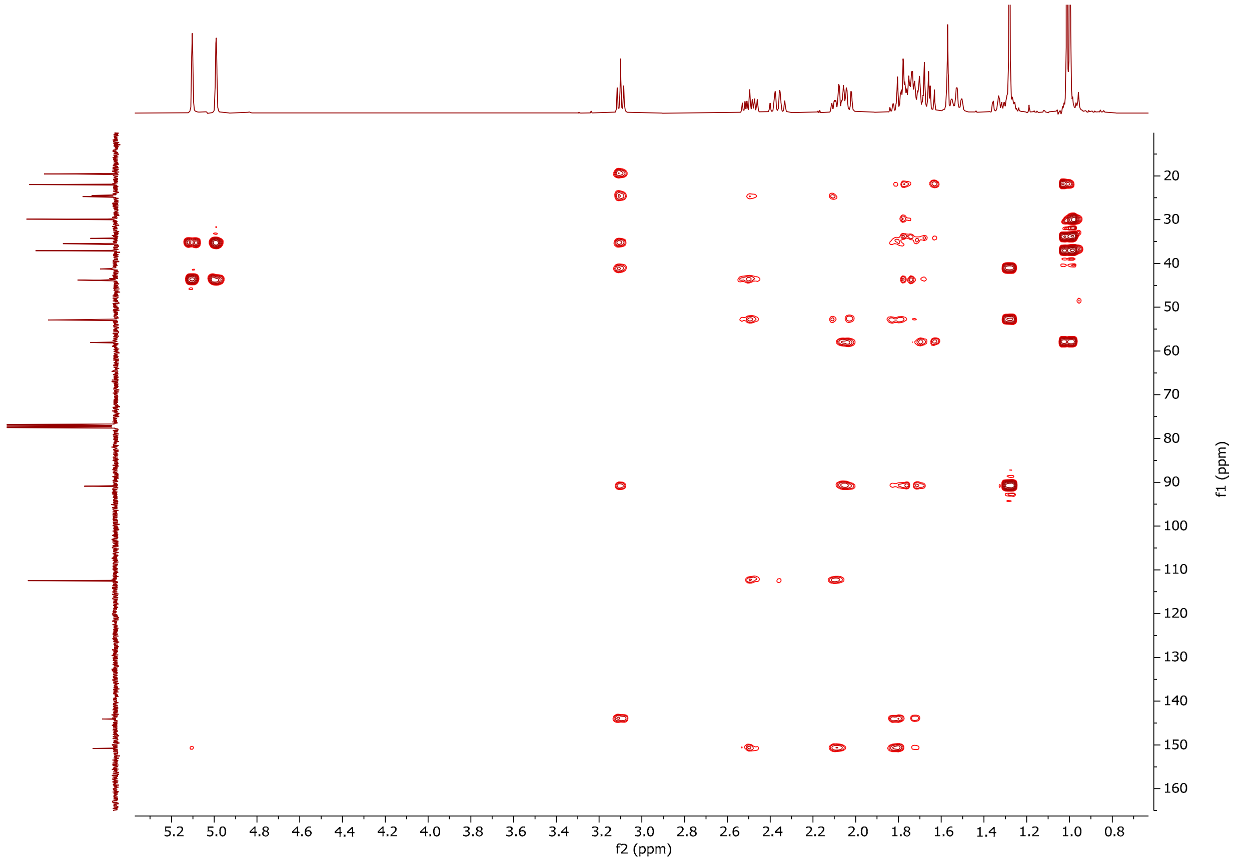

Figure S32. HMBC ^{1}H-^{13}C NMR spectra of compound **19b**.

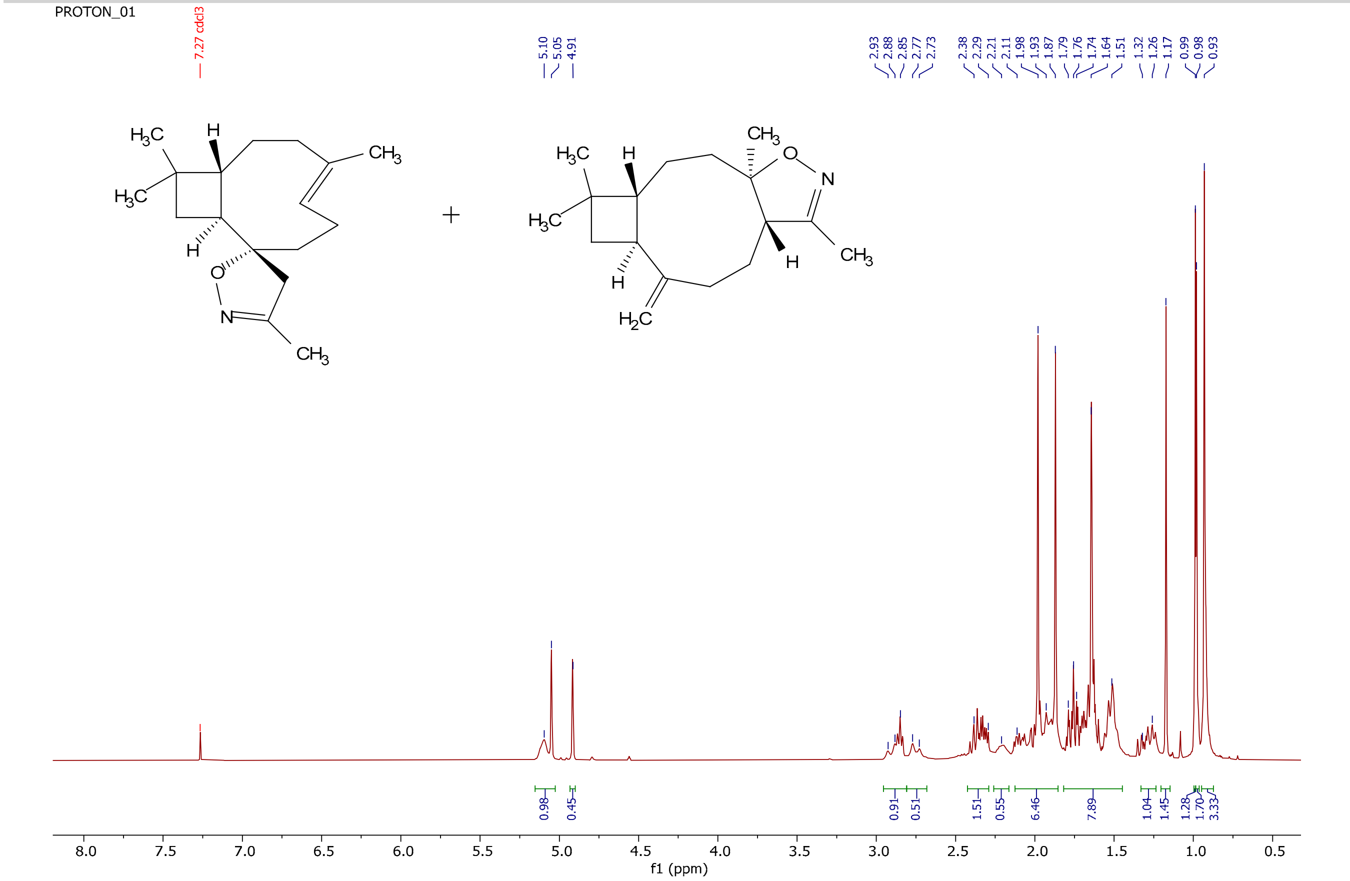

Figure S33. ^{1}H NMR spectra of mixture of compounds **20a** and **20b**.

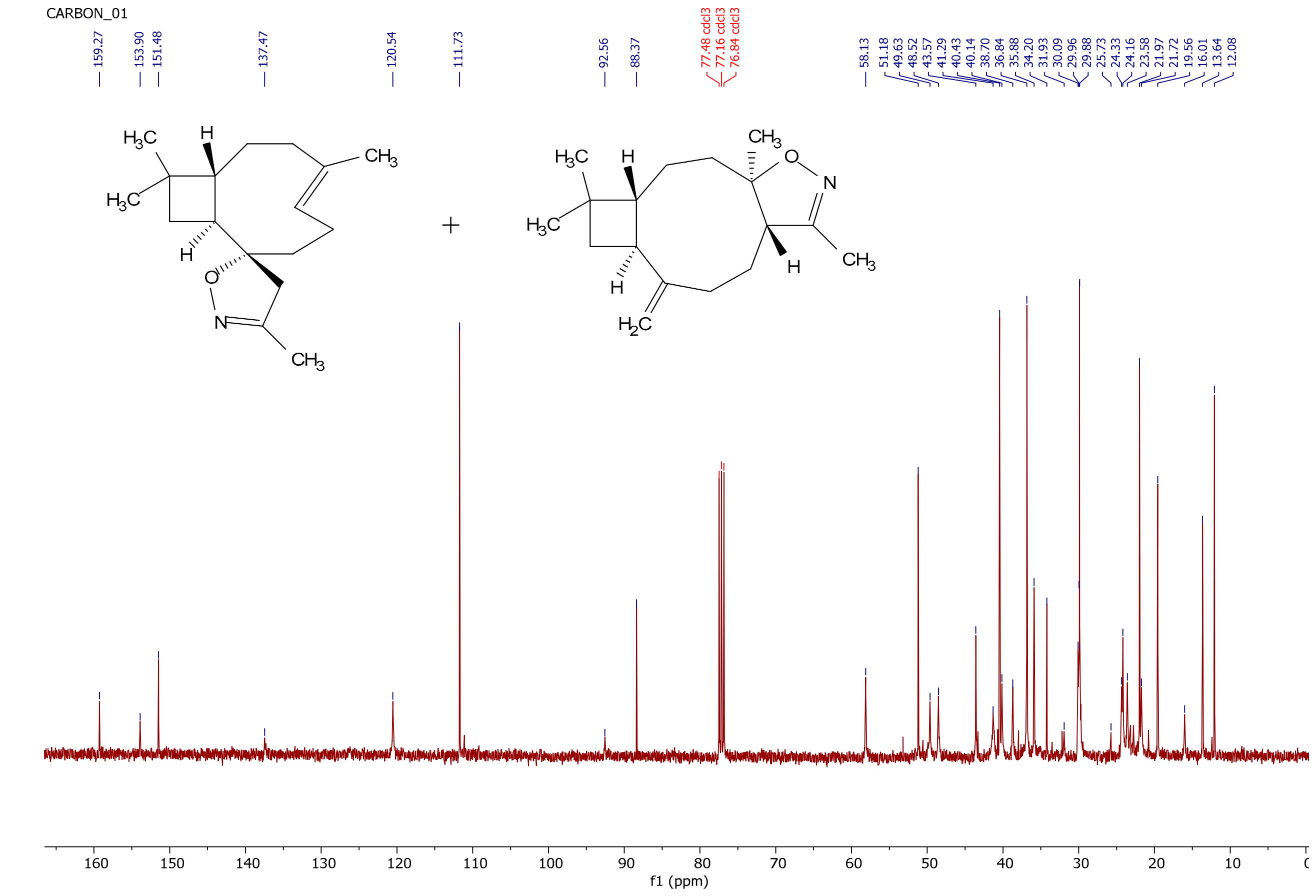

Figure S34. ^{13}C NMR spectra of mixture of compounds **20a** and **20b**.

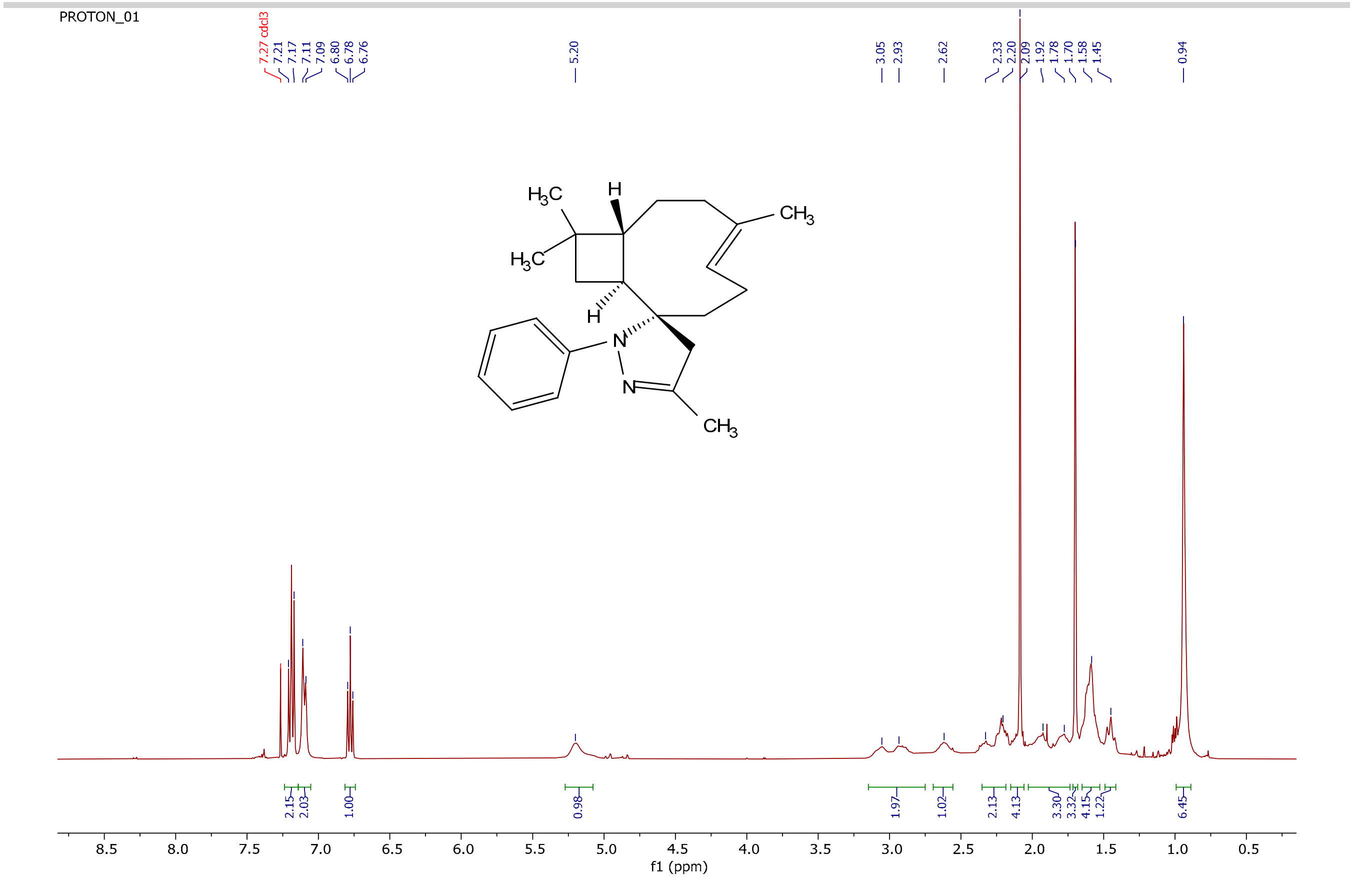

Figure S35. ^{1}H NMR spectra of compound **21a.**

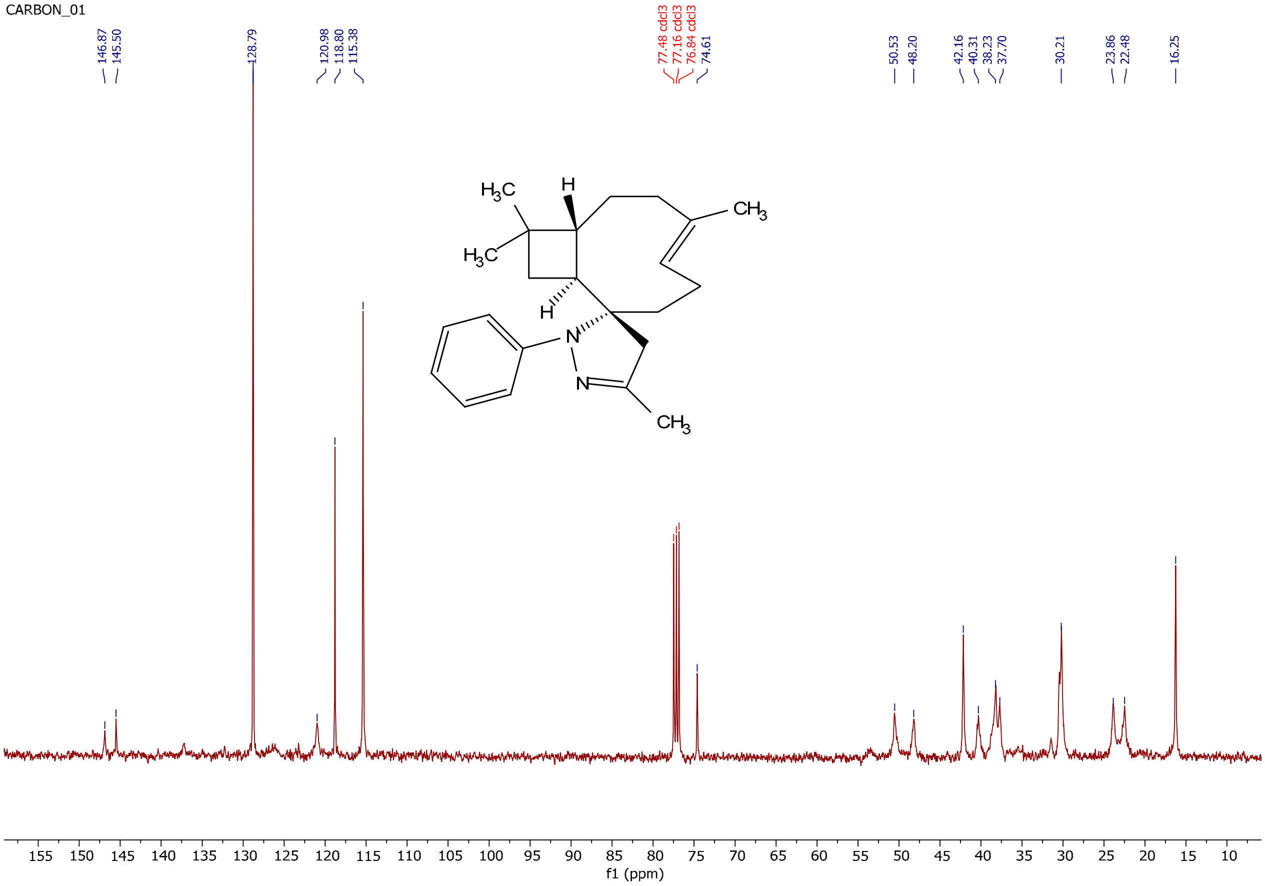

Figure S36. ^{13}C NMR spectra of compound **21a.**

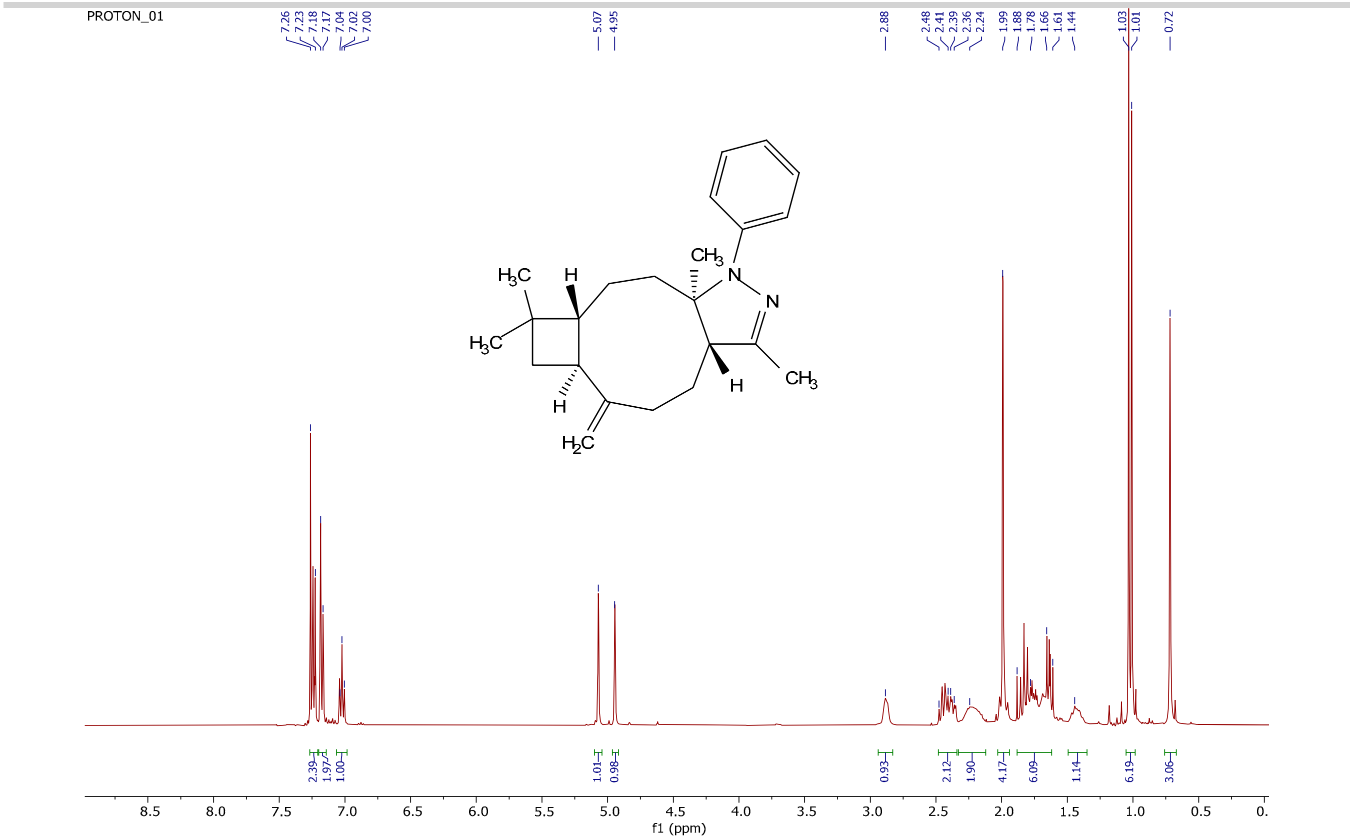

Figure S37. ^{1}H NMR spectra of compound **21b.**

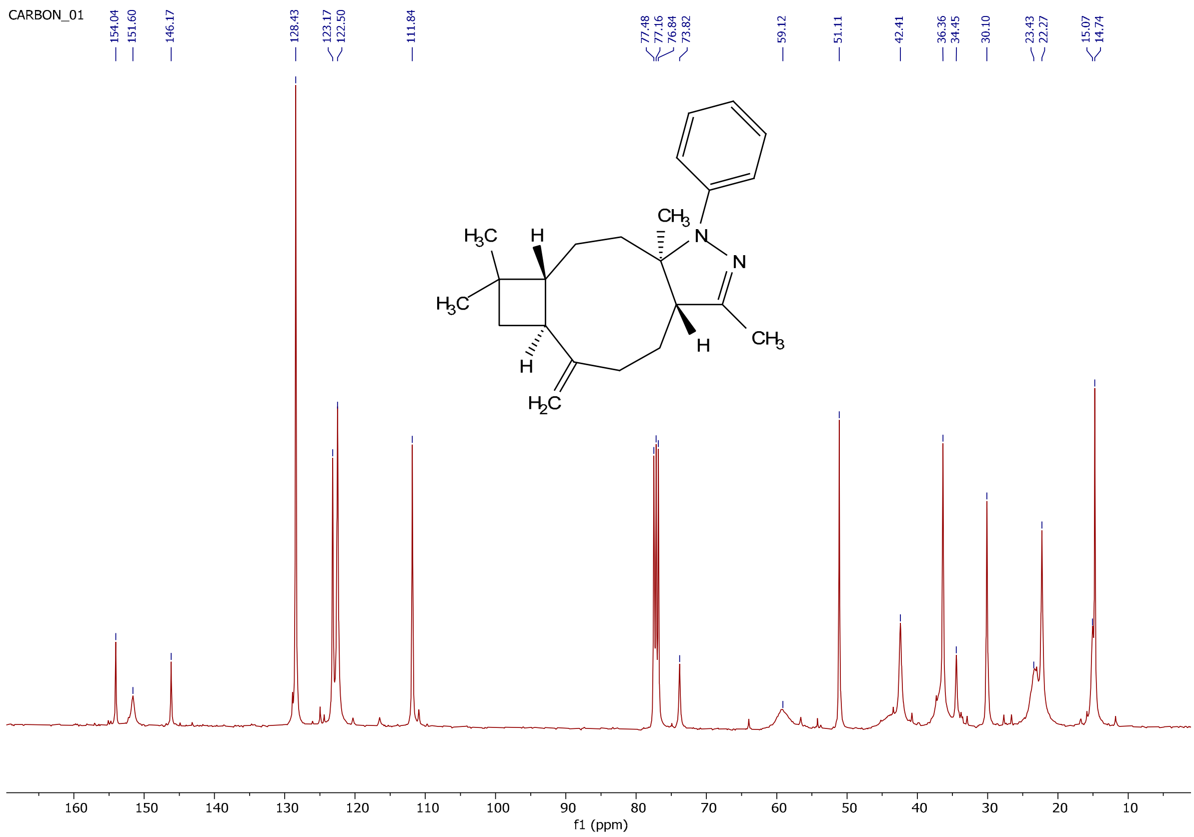

Figure S38. ^{13}C NMR spectra of compound **21b.**

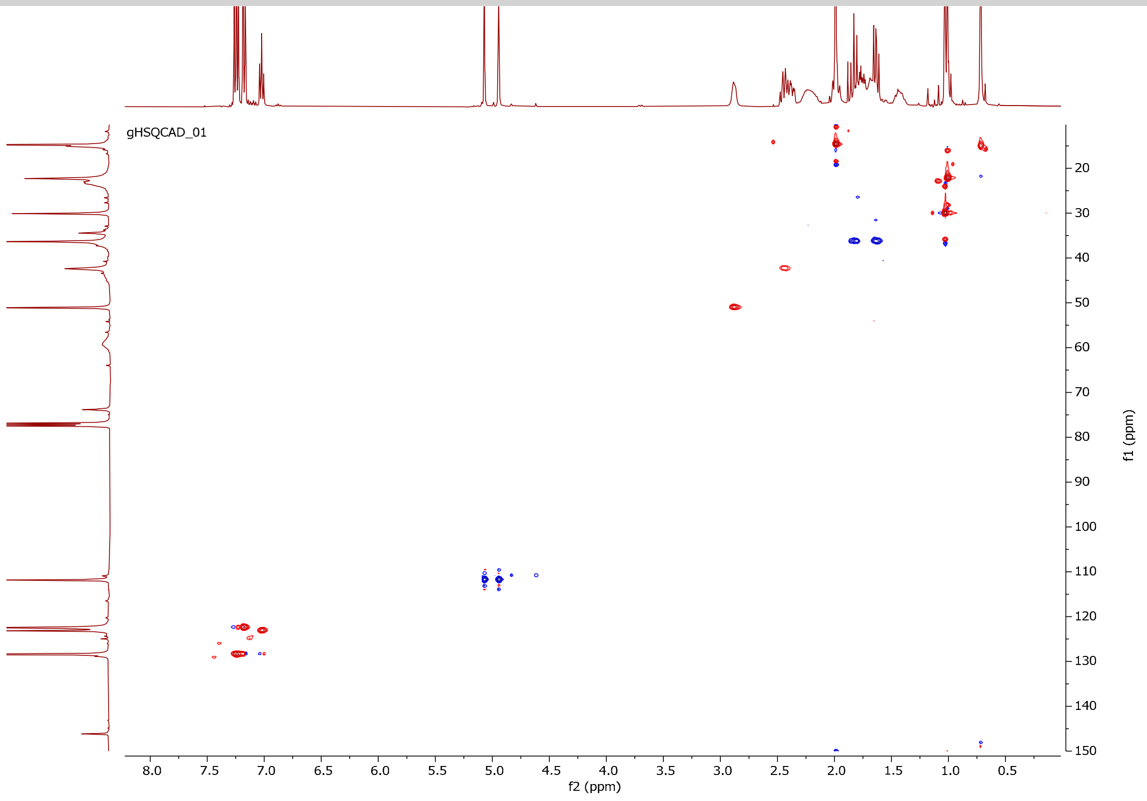

Figure S39. HSQC ^{1}H-^{13}C NMR spectra of compound **21b**.

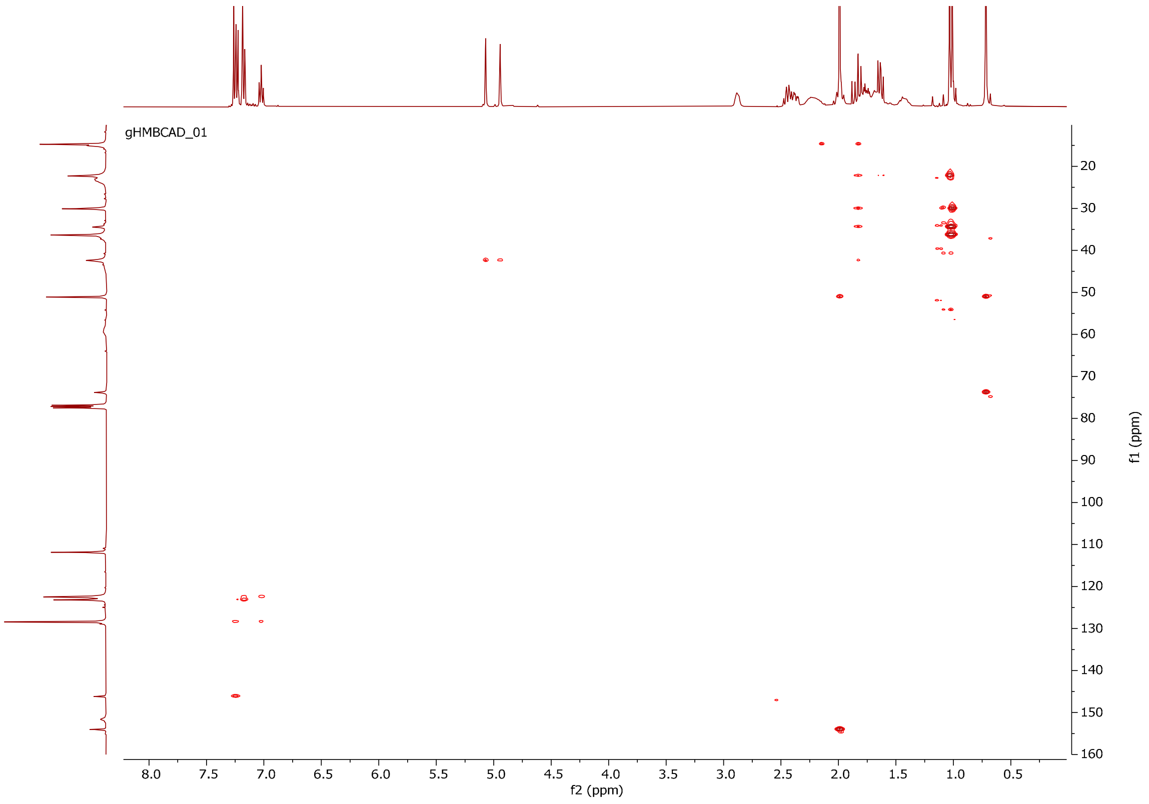

Figure S40. HMBC ^{1}H-^{13}C NMR spectra of compound **21b**.

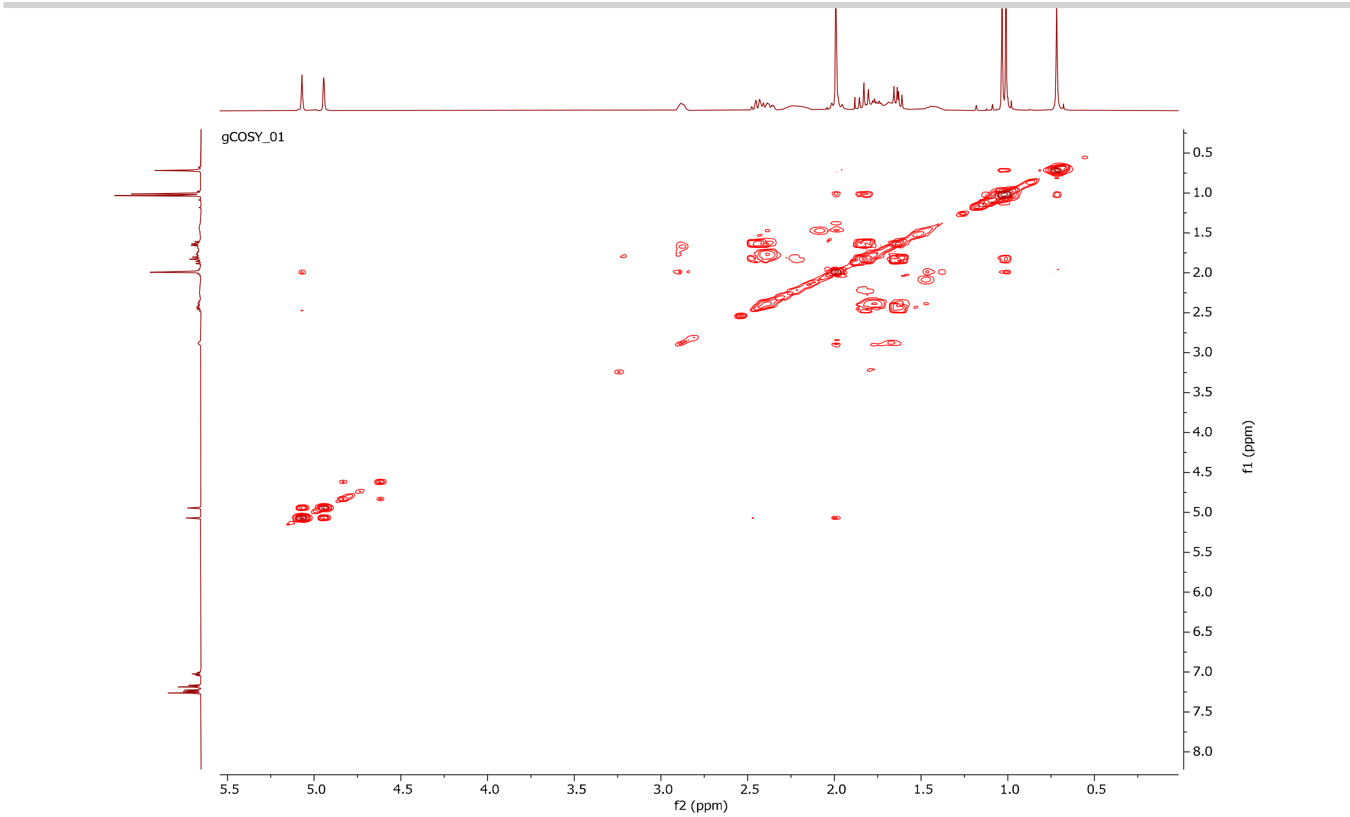

Figure S41. COESY ^{1}H-^{1}H NMR spectra of compound **21b**.

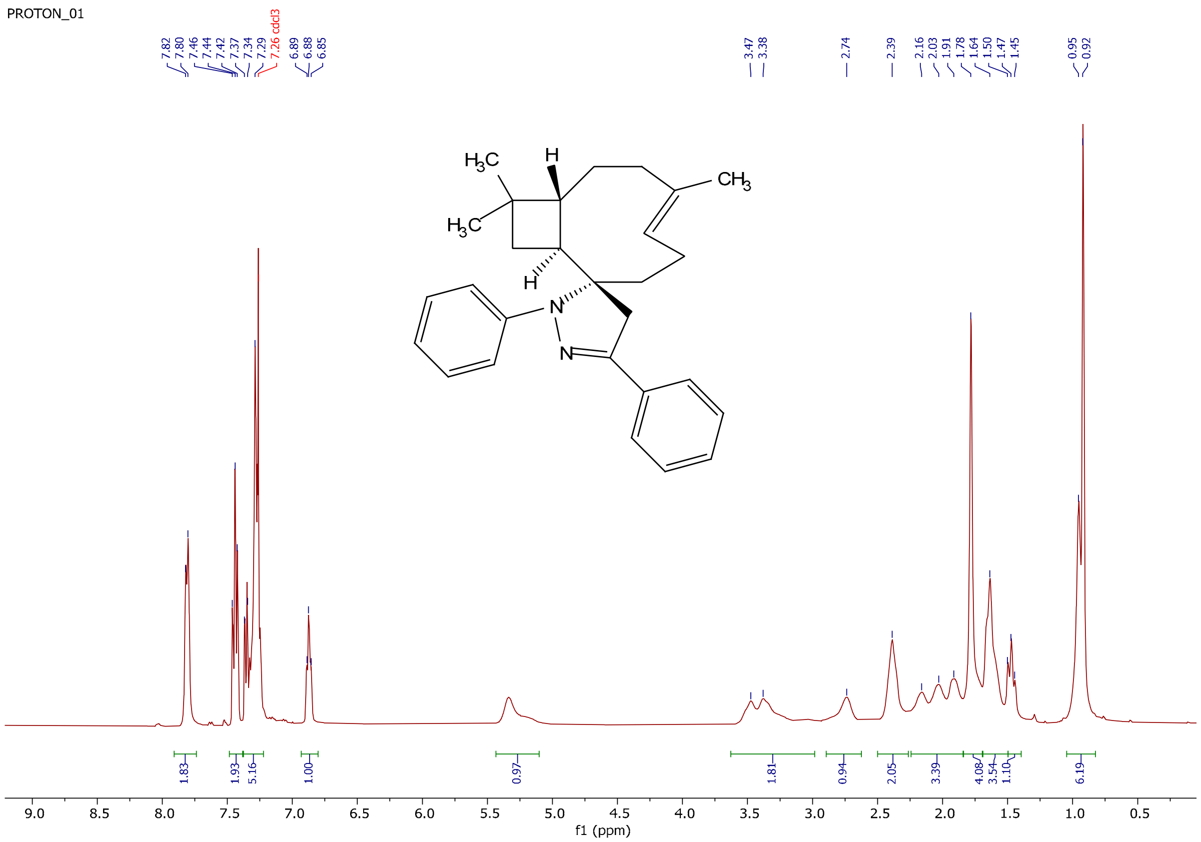

Figure S42. ^{1}H NMR spectra of compound **22a.**

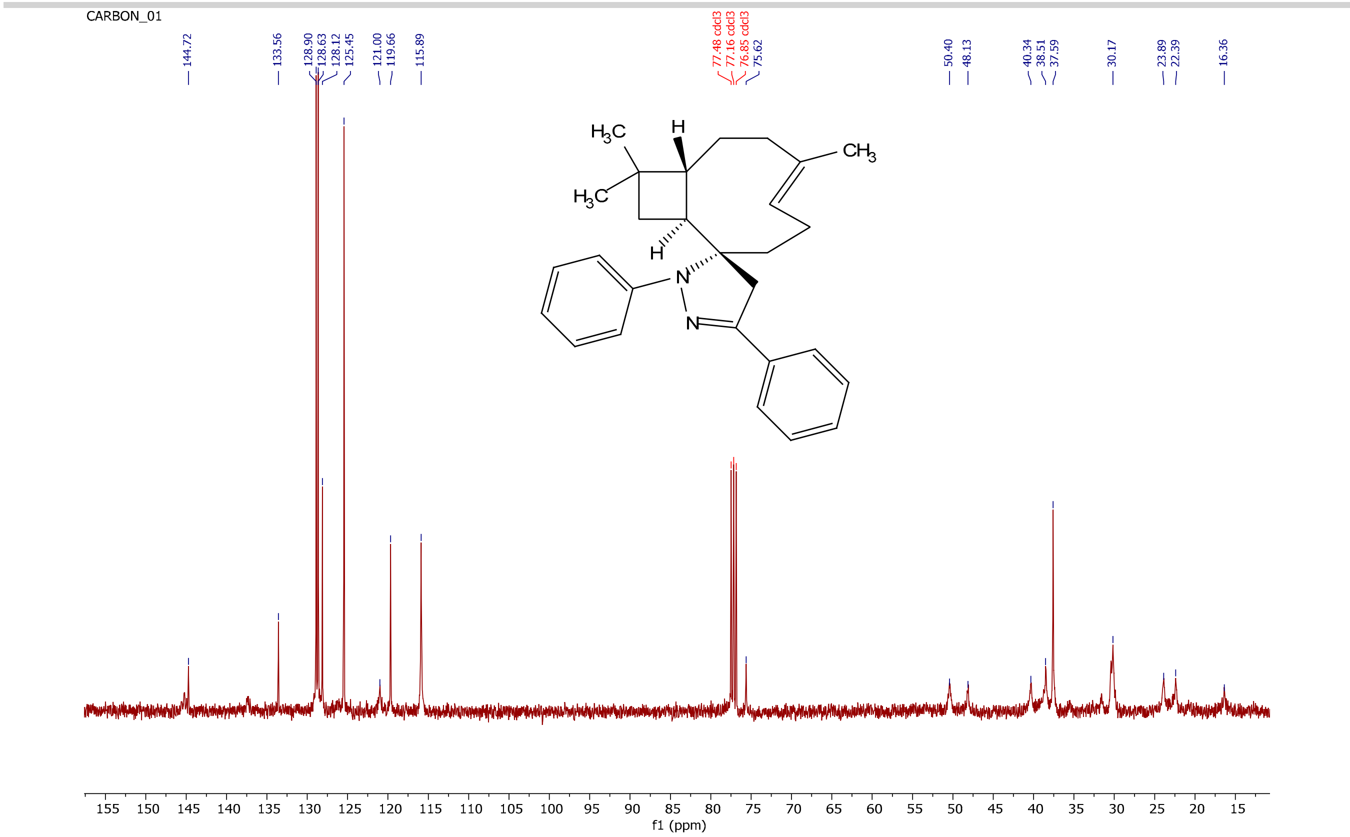

Figure S43. ^{13}C NMR spectra of compound **22a.**

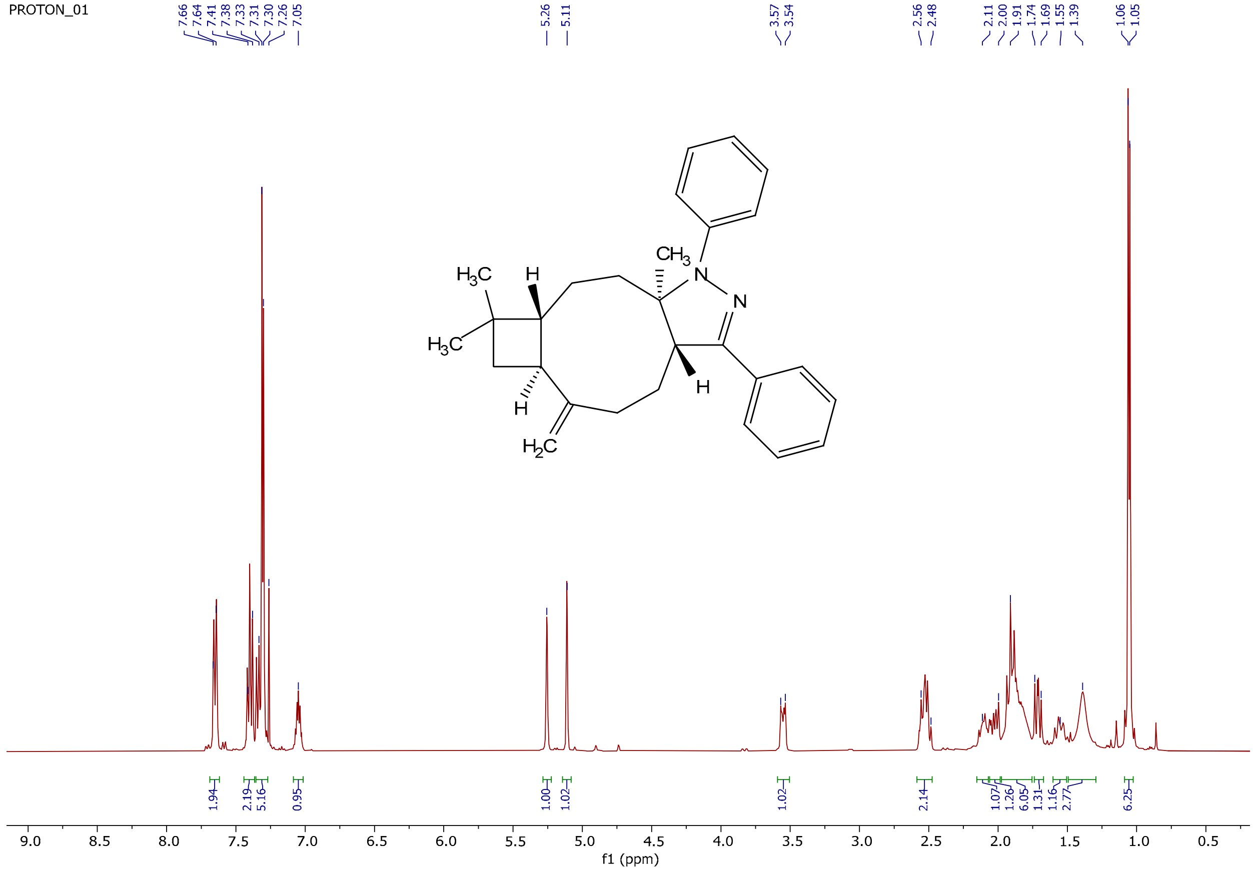

Figure S44. ^{1}H NMR spectra of compound **22b.**

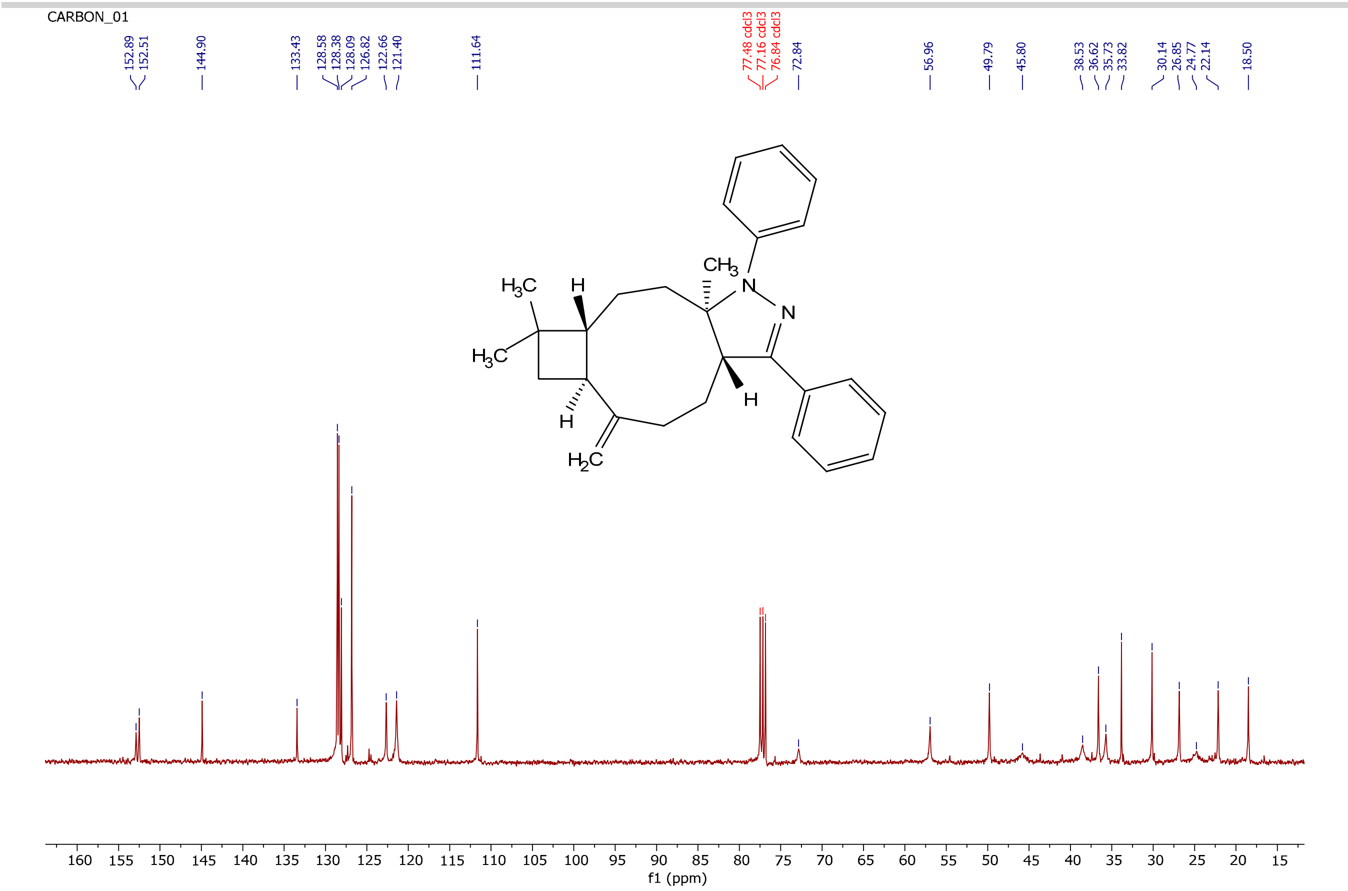

Figure S45. ^{13}C NMR spectra of compound **22b.**

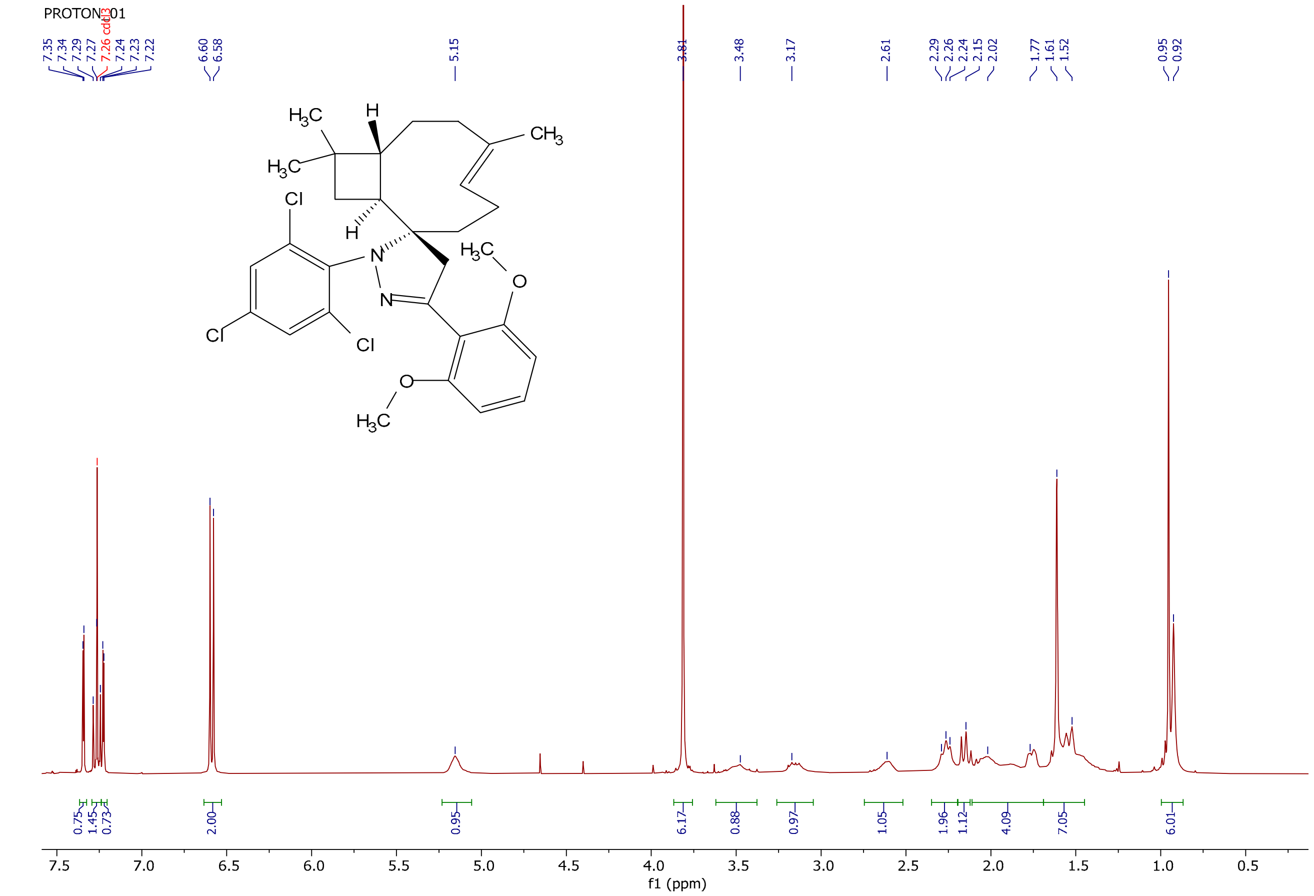

Figure S46. ^{1}H NMR spectra of compound **23a.**

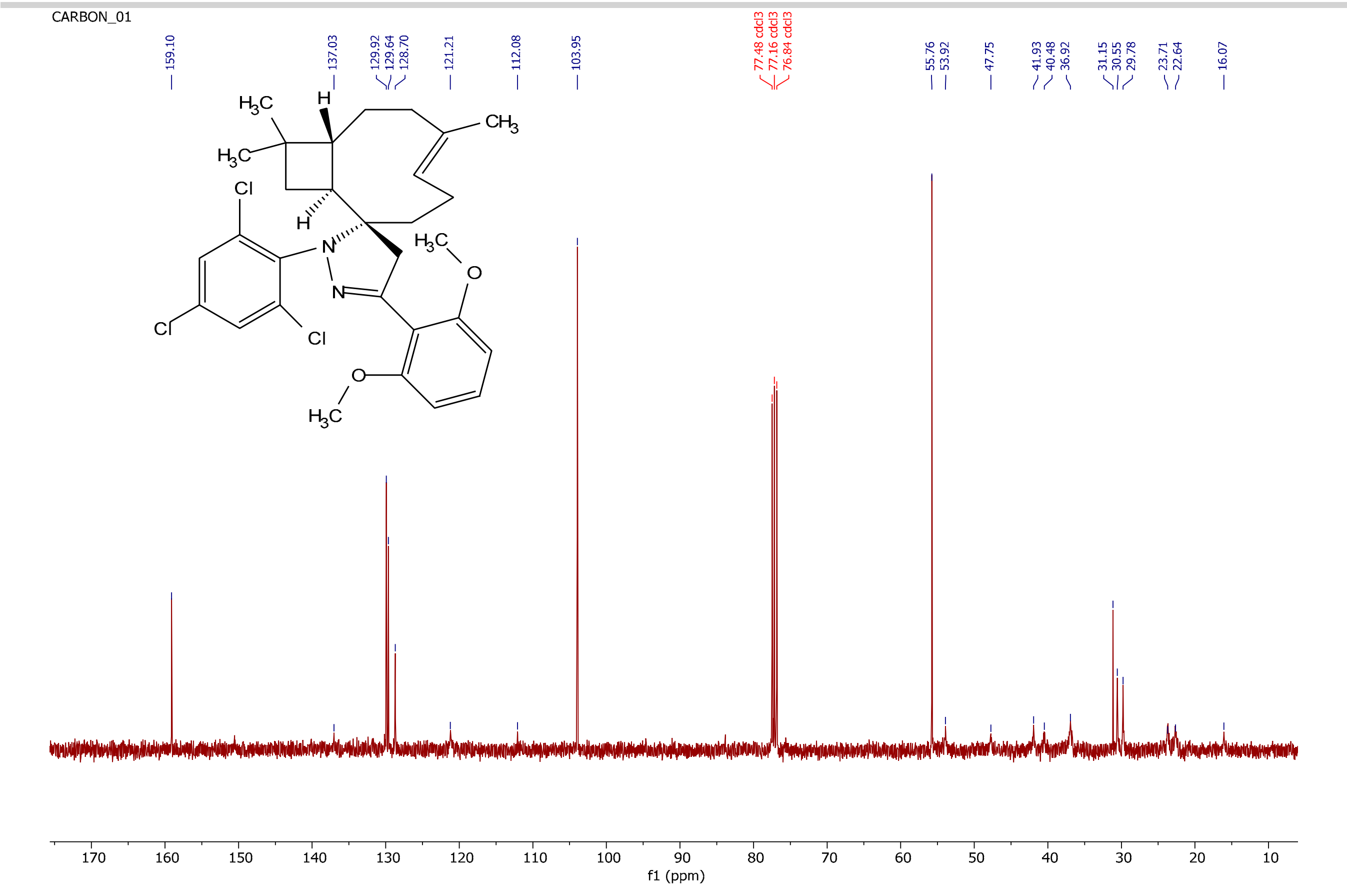

Figure S47. ^{13}C NMR spectra of compound **23a.**

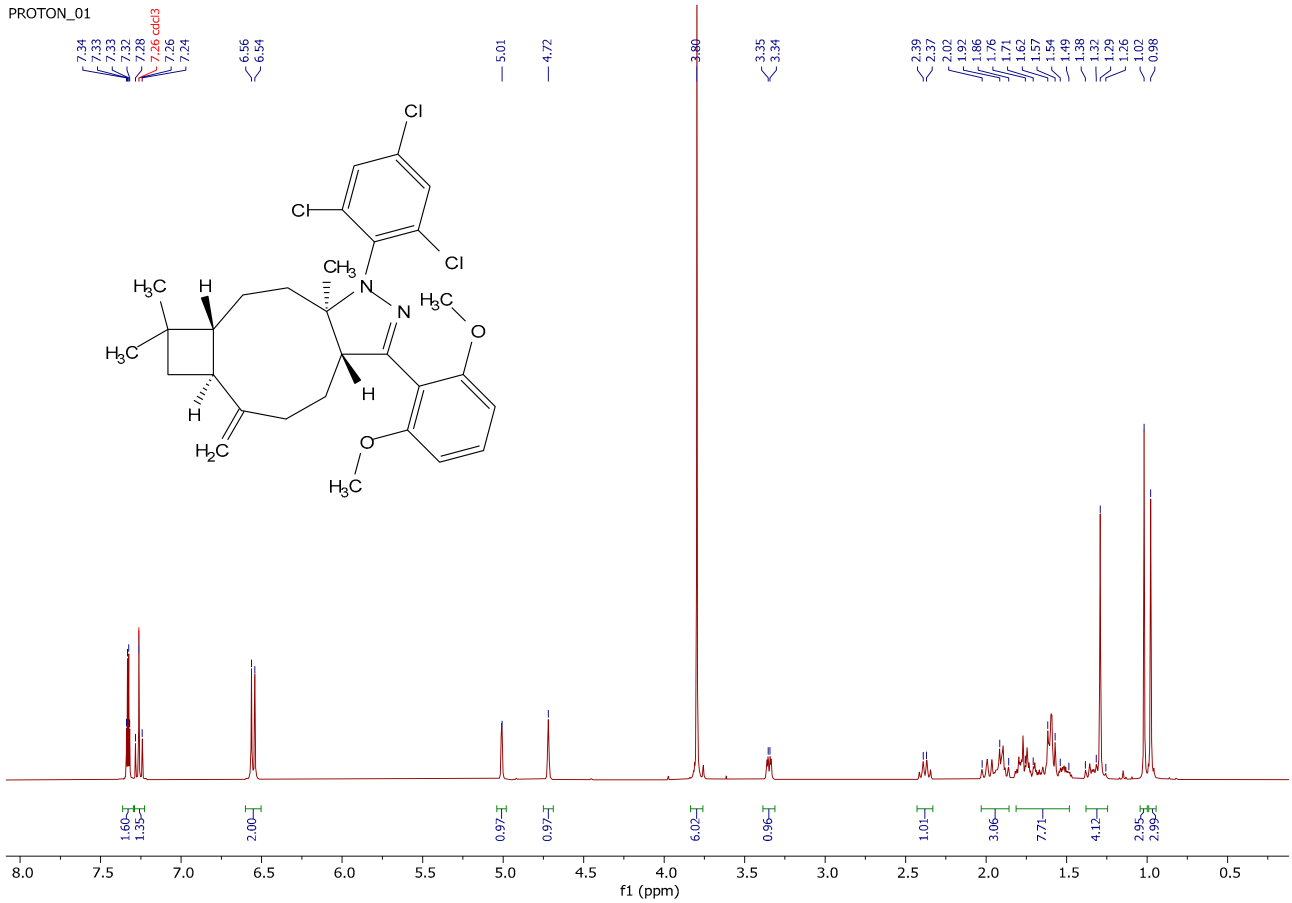

Figure S48. ^{1}H NMR spectra of compound **23b.**

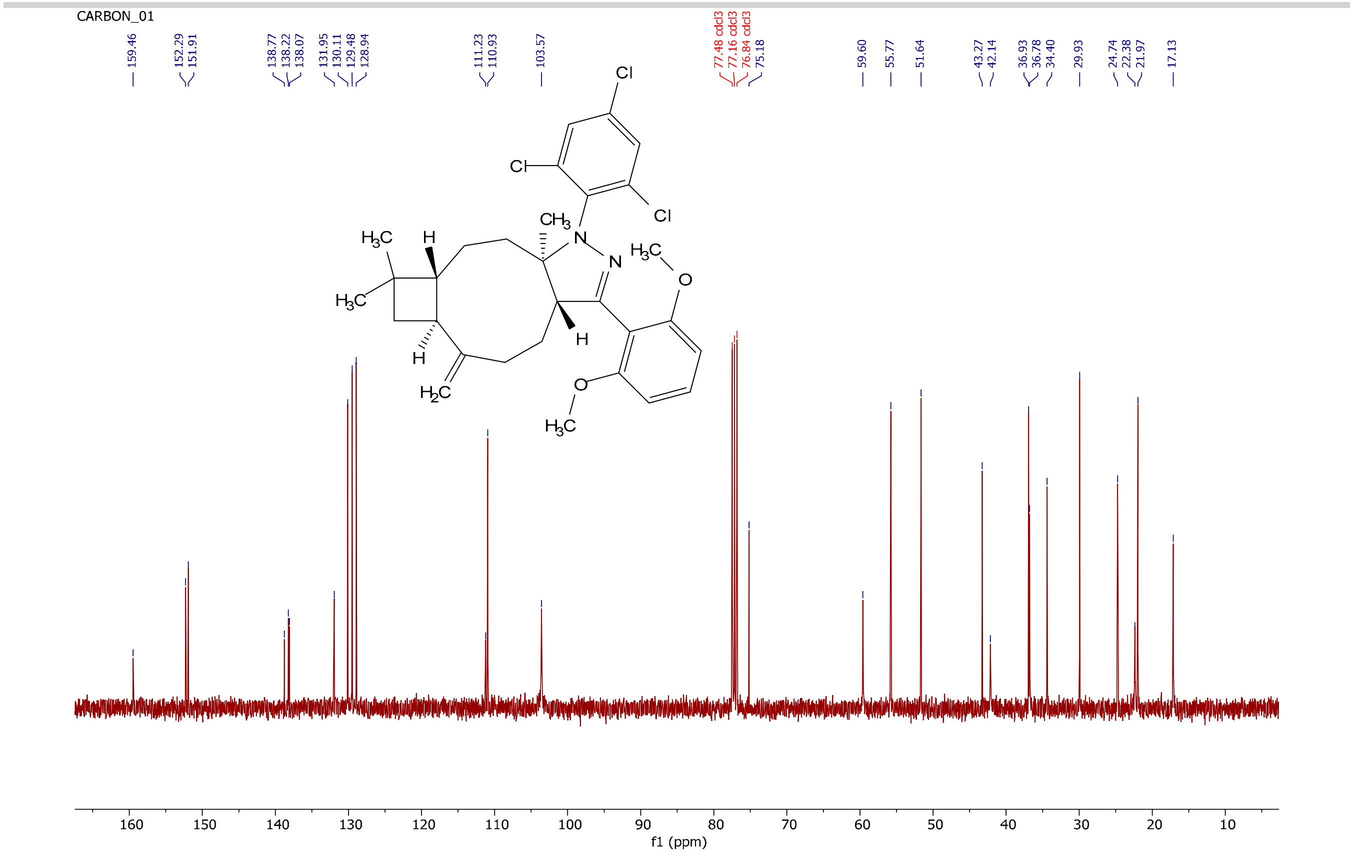

Figure S49. ^{13}C NMR spectra of compound **23b.**

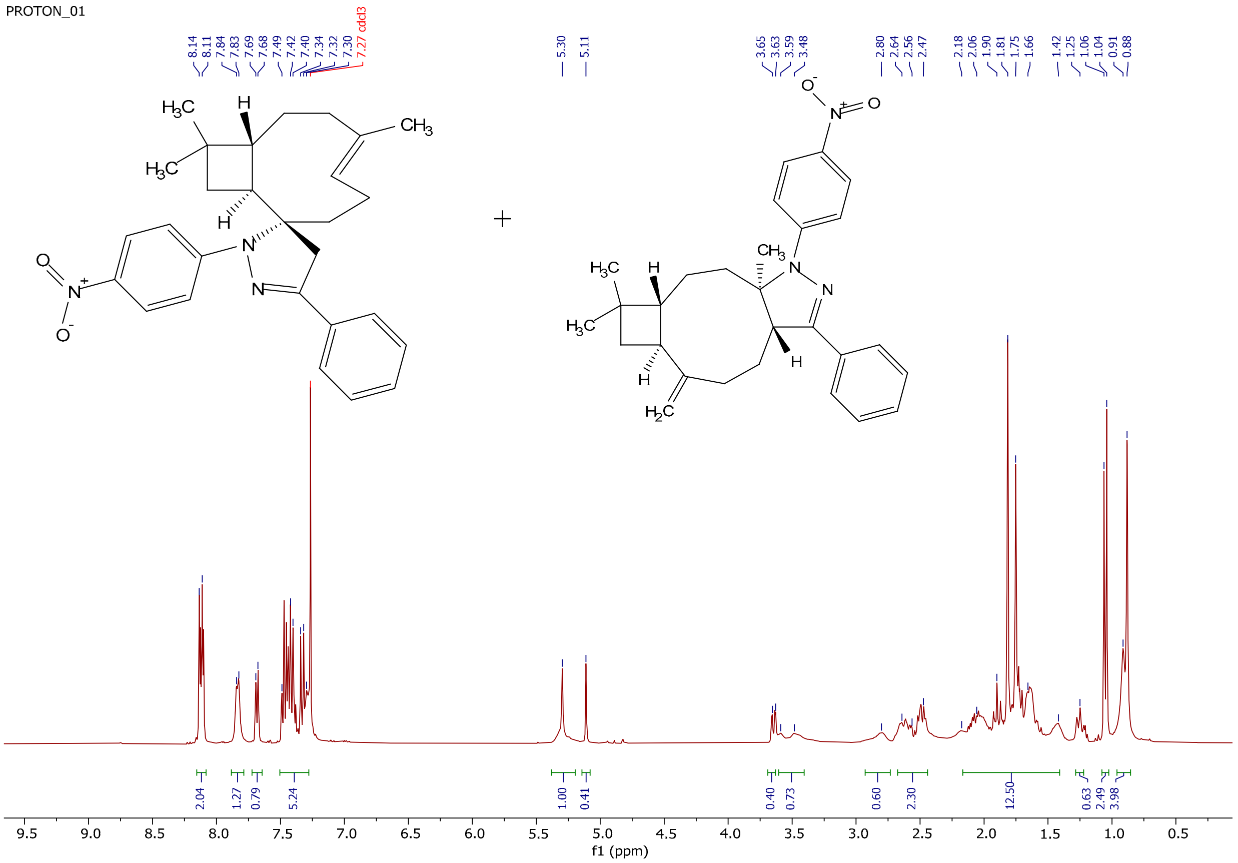

Figure S50. ^{1}H NMR spectra of mixture of compounds **24a** and **24b**.

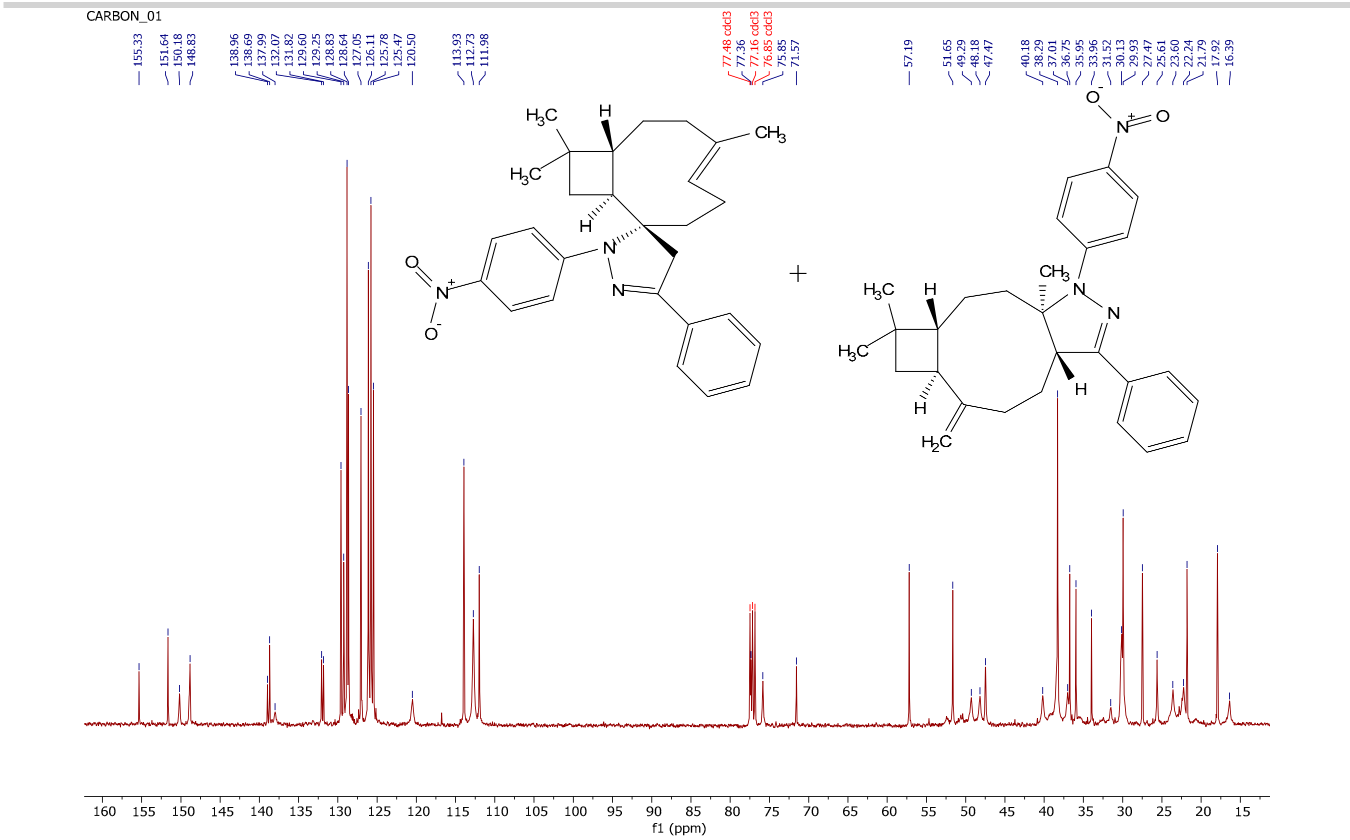

Figure S51. ^{13}C NMR spectra of mixture of compounds **24a** and **24b**.

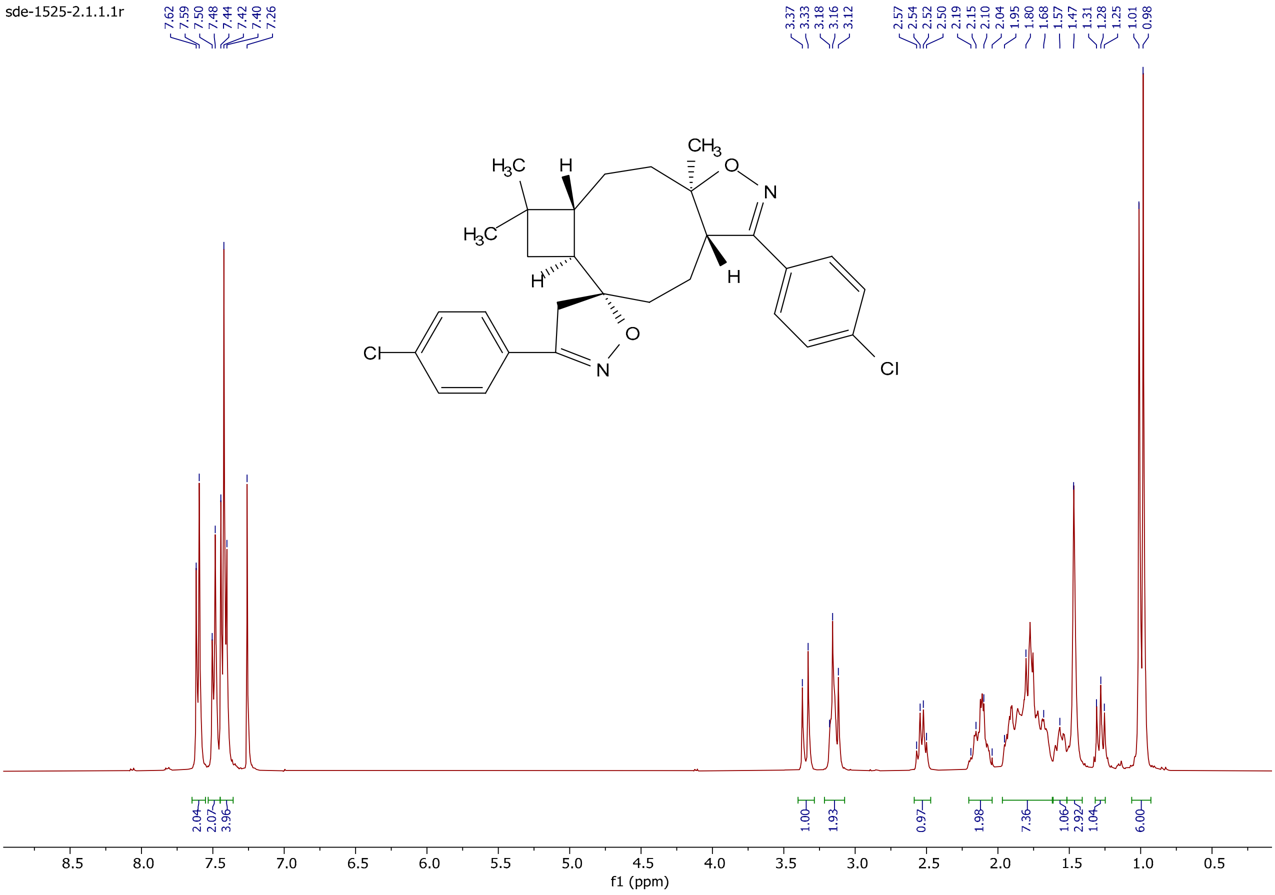

Figure S52. ^{1}H NMR spectra of compound **25.**

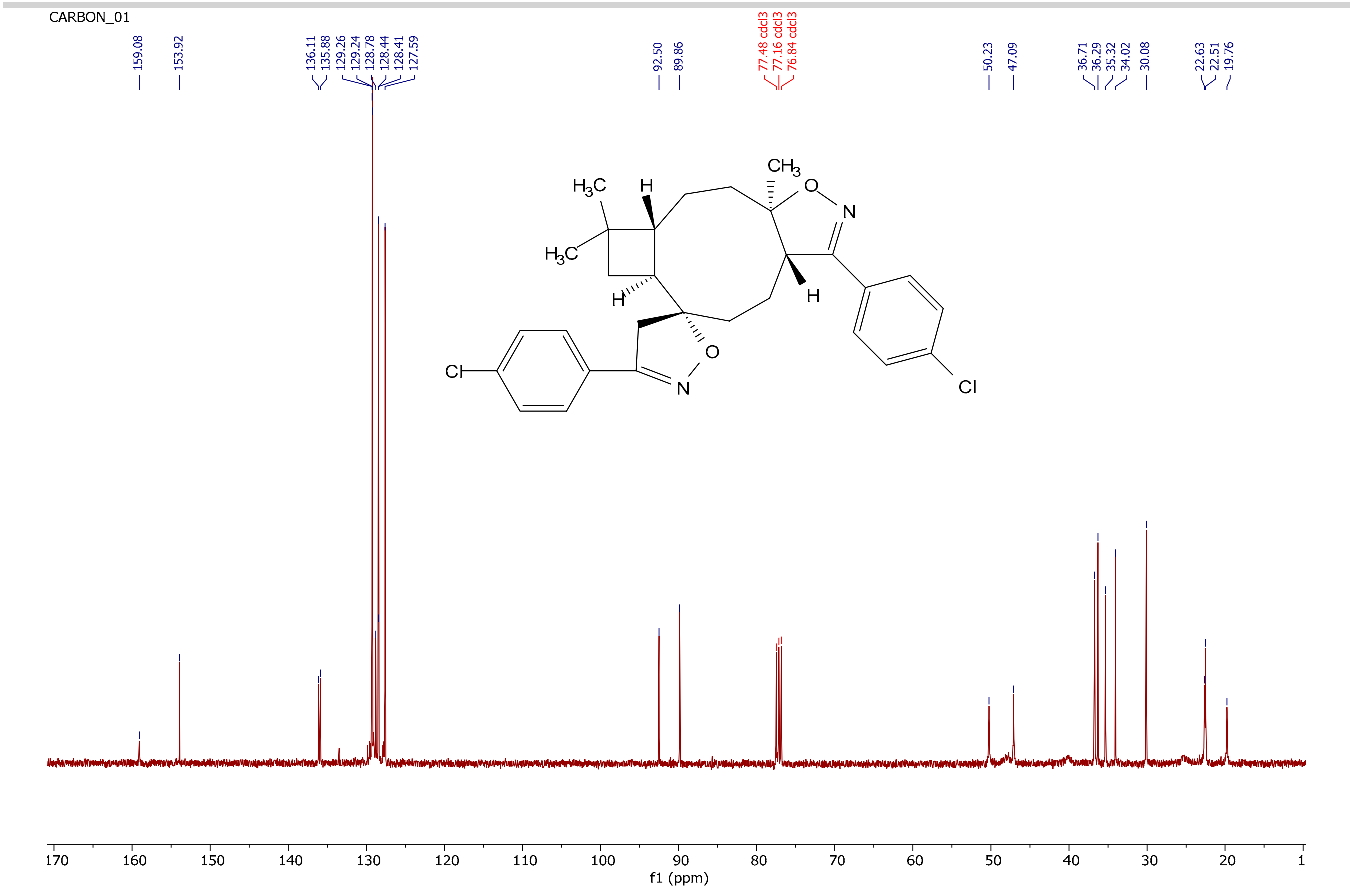

Figure S53. ^{13}C NMR spectra of compound **25.**

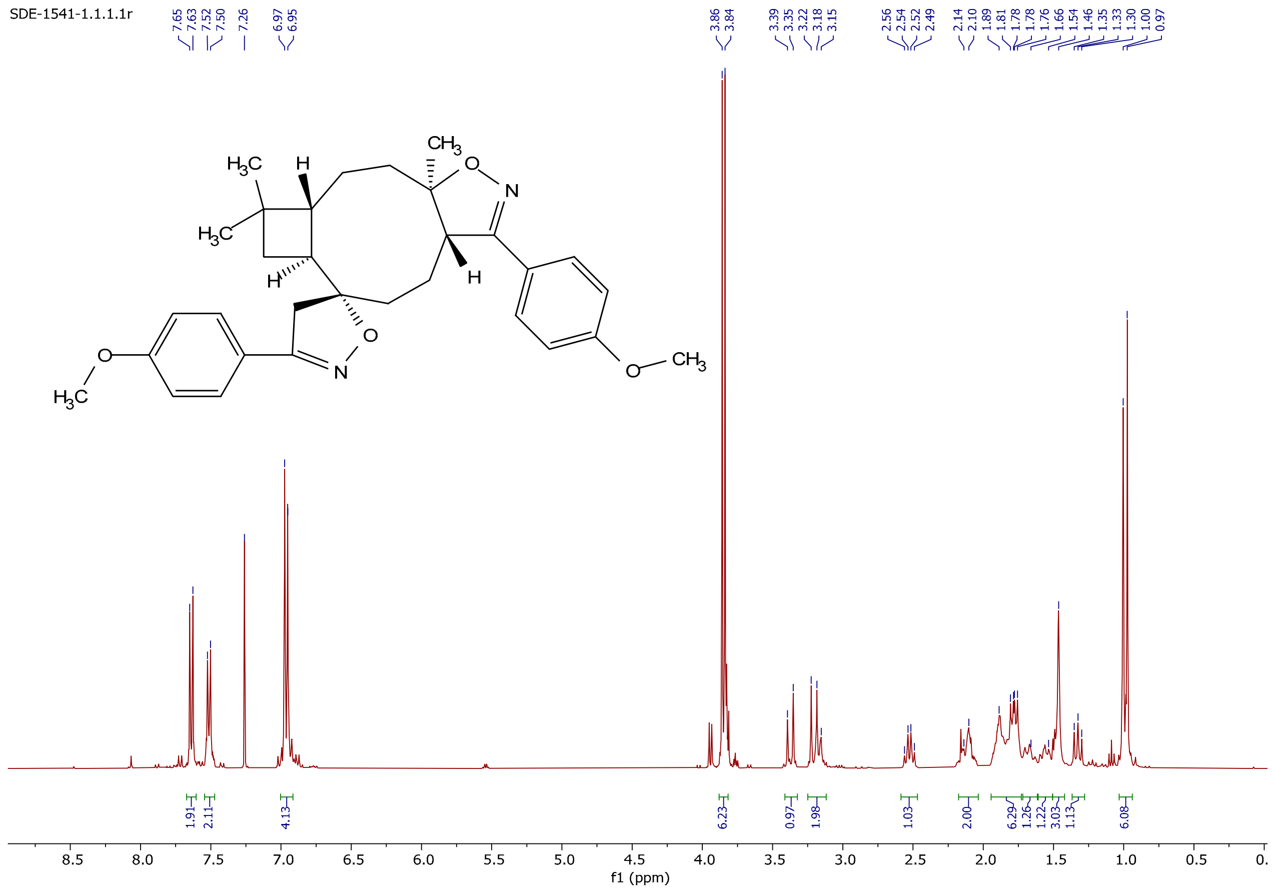

Figure S54. ^{1}H NMR spectra of compound **26.**

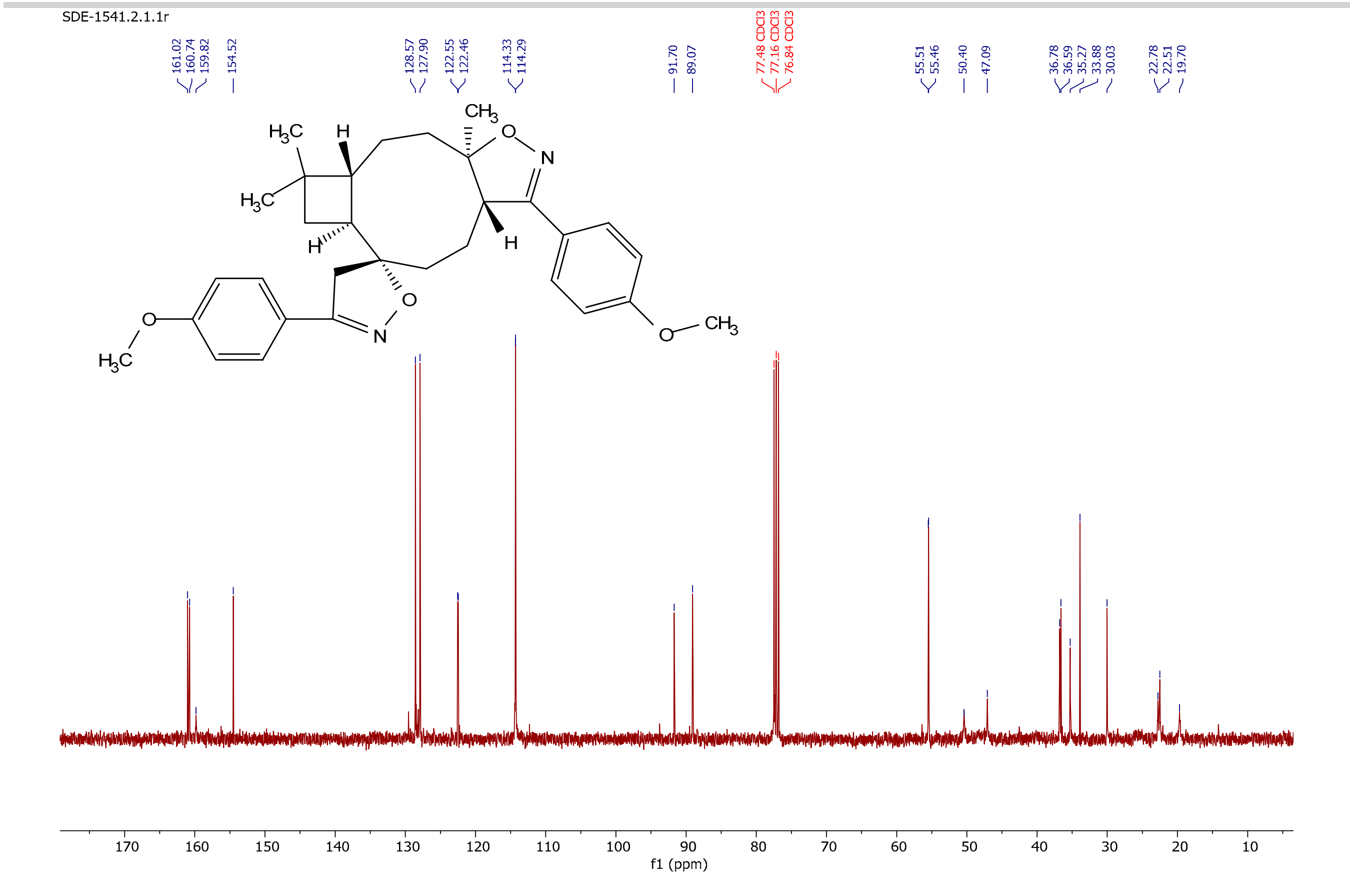

Figure S55. ^{13}C NMR spectra of compound **26.**

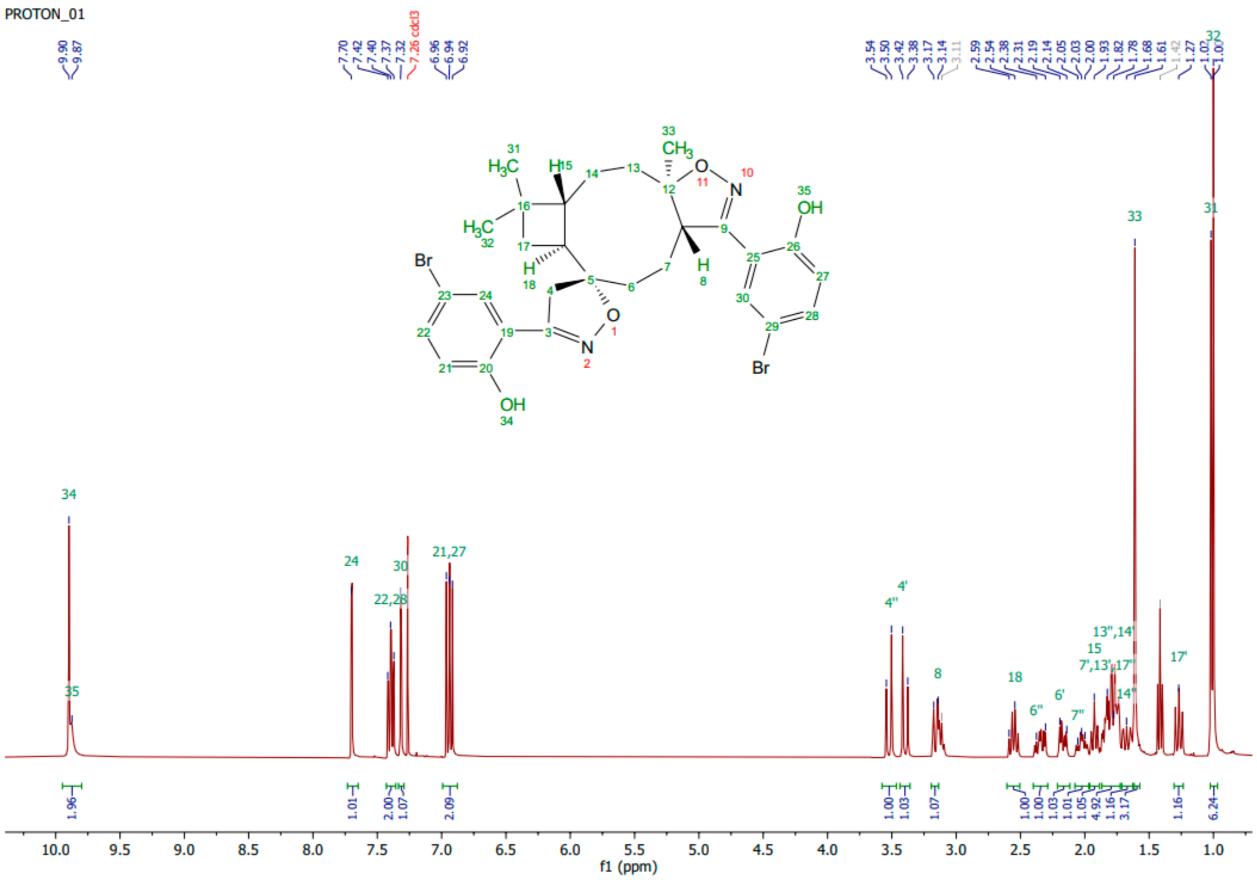

Figure S56. ^{1}H NMR spectra of compound **27.**

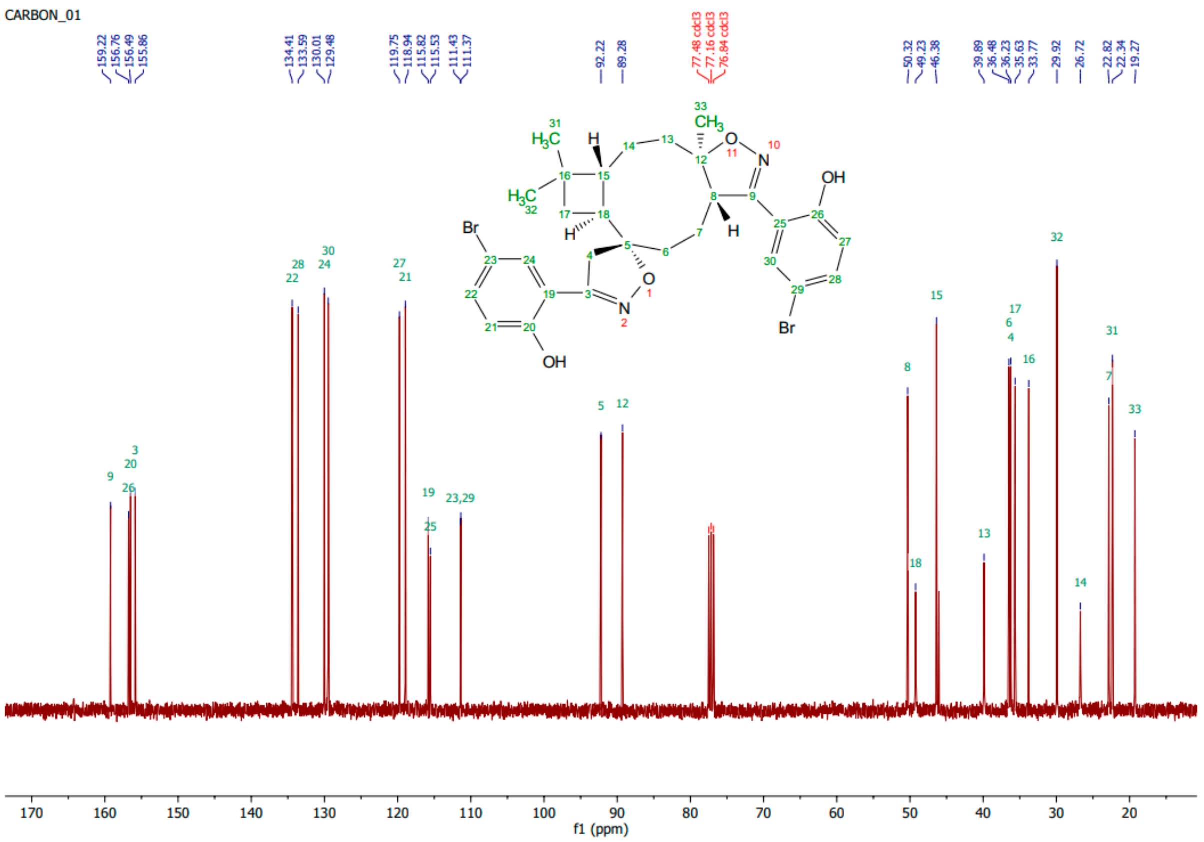

Figure S67. ^{13}C NMR spectra of compound **27.**

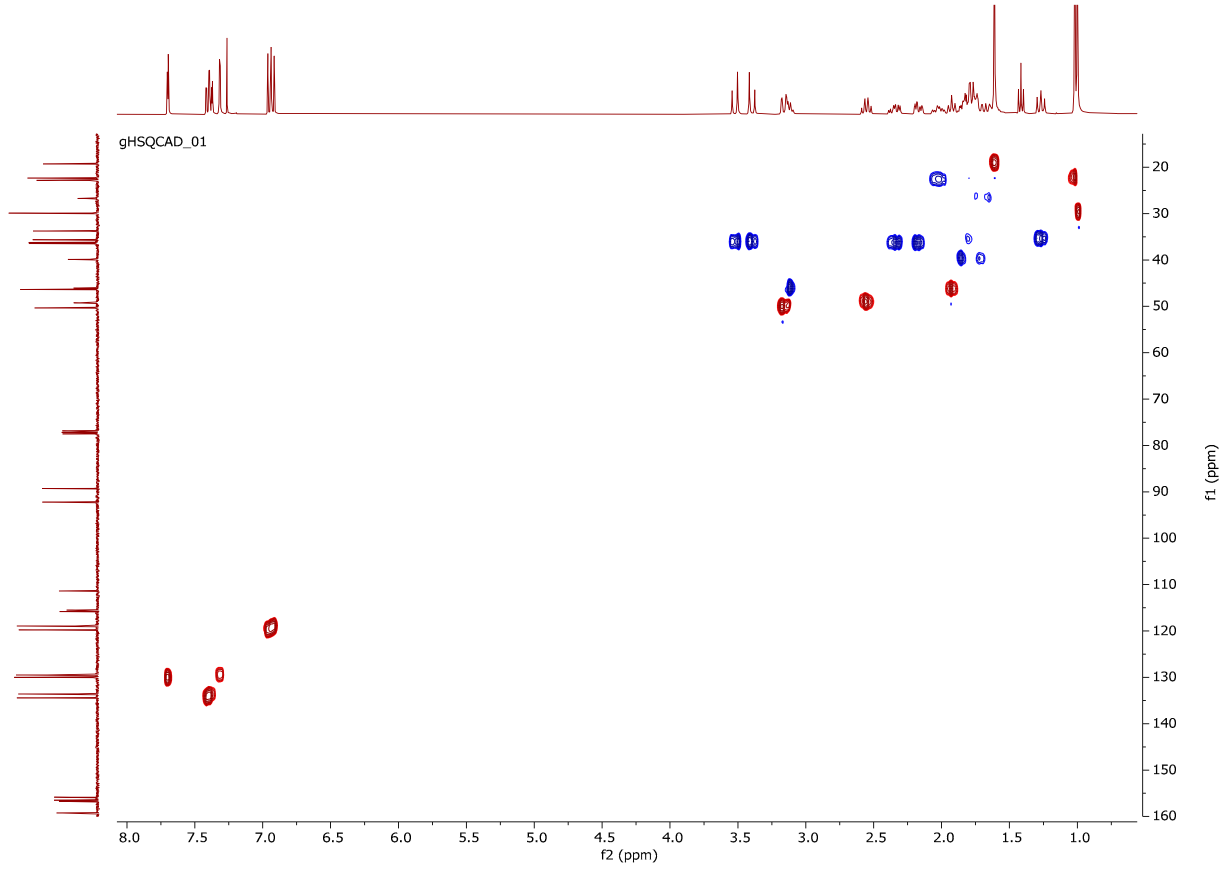

Figure S58. HSQC ^{1}H-^{13}C NMR spectra of compound **27**.

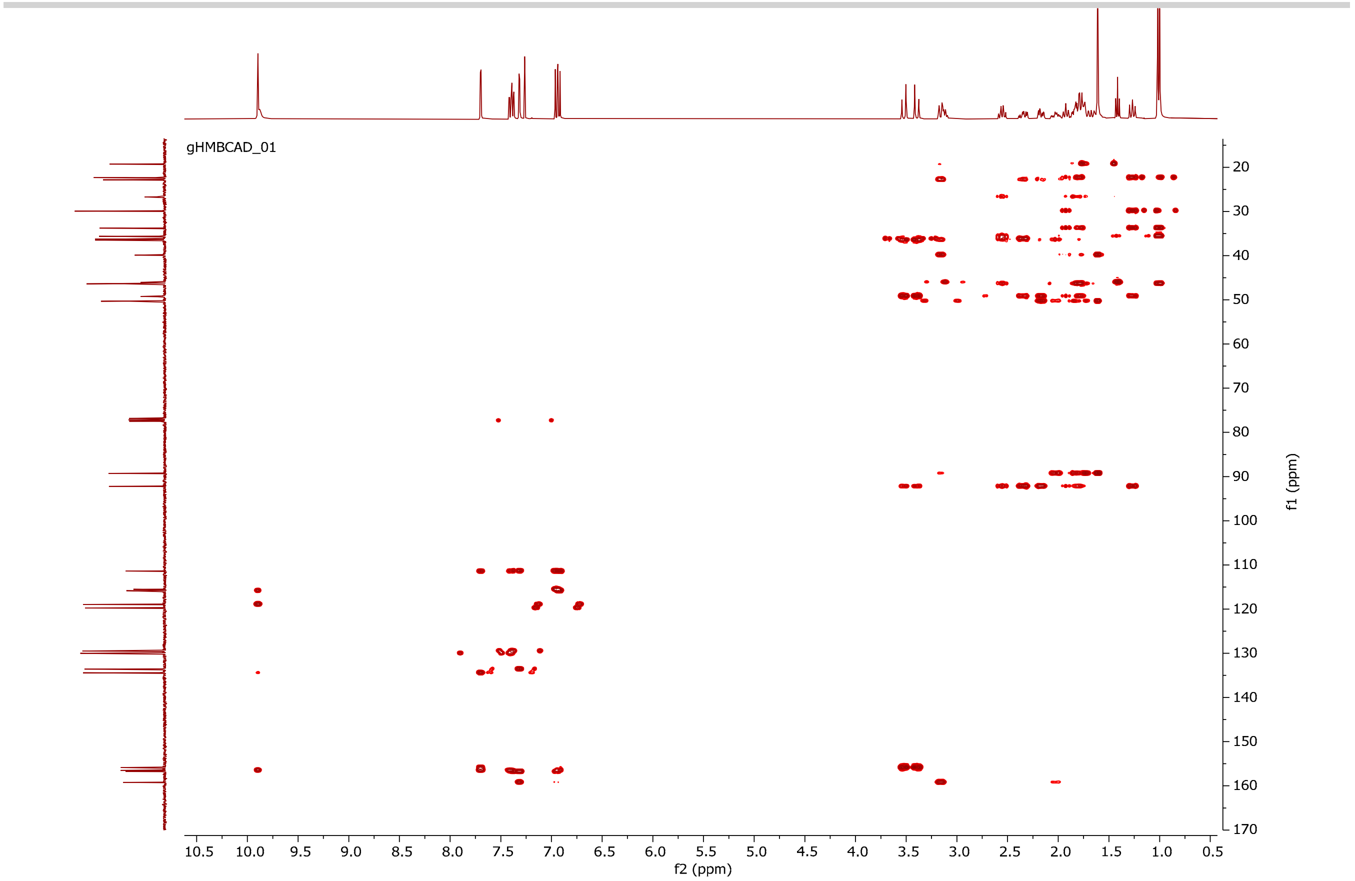

Figure S59. HMBC ^{1}H-^{13}C NMR spectra of compound **27**.

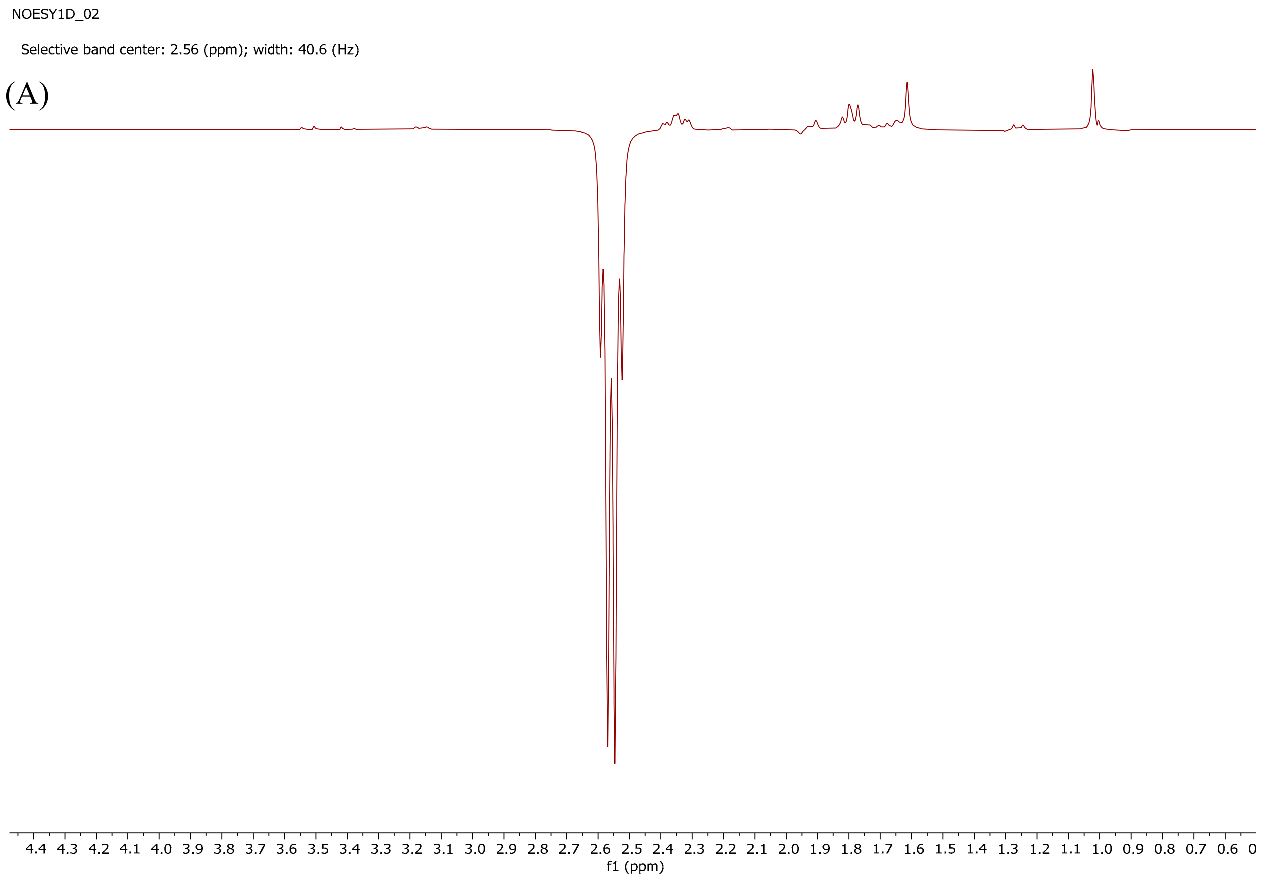

NOESY1D_03

Selective band center: 3.52 (ppm); width: 27.6 (Hz)

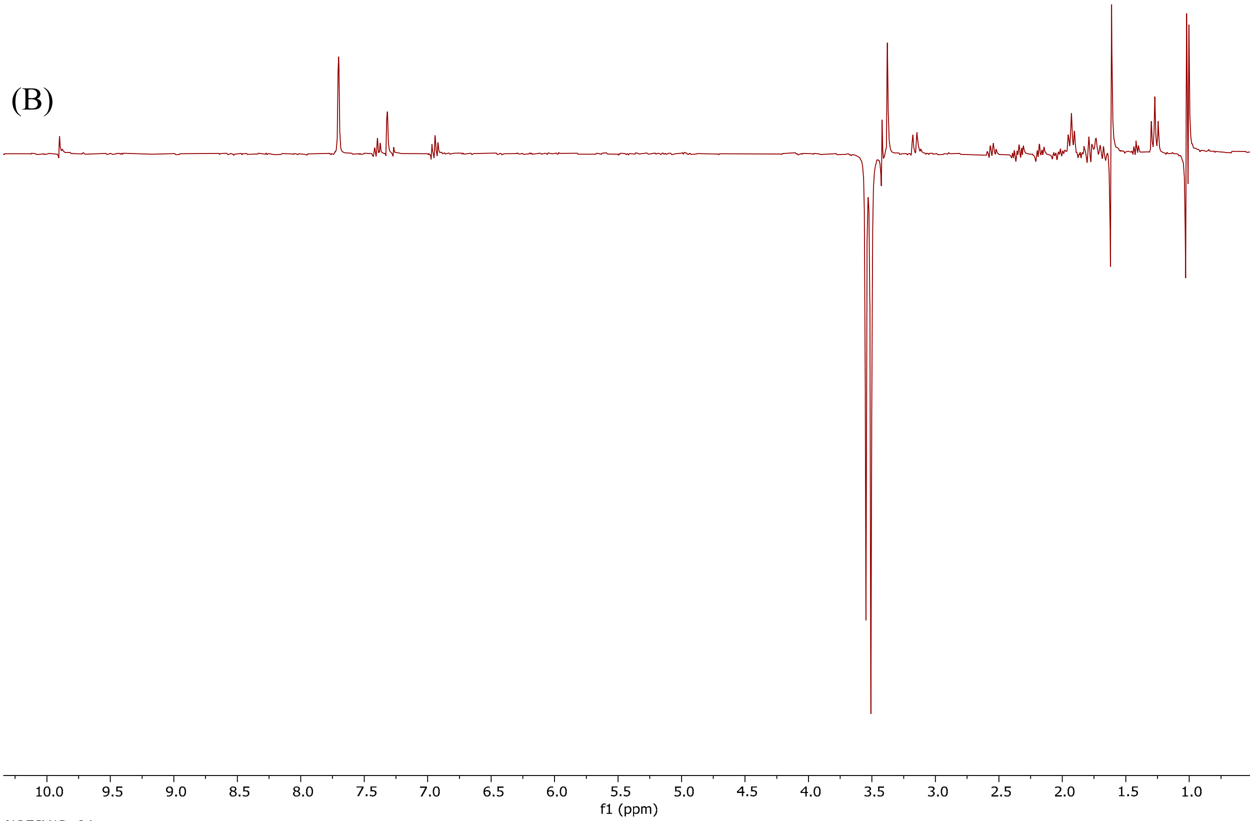

NOESY1D_04

Selective band center: 3.40 (ppm); width: 23.6 (Hz)

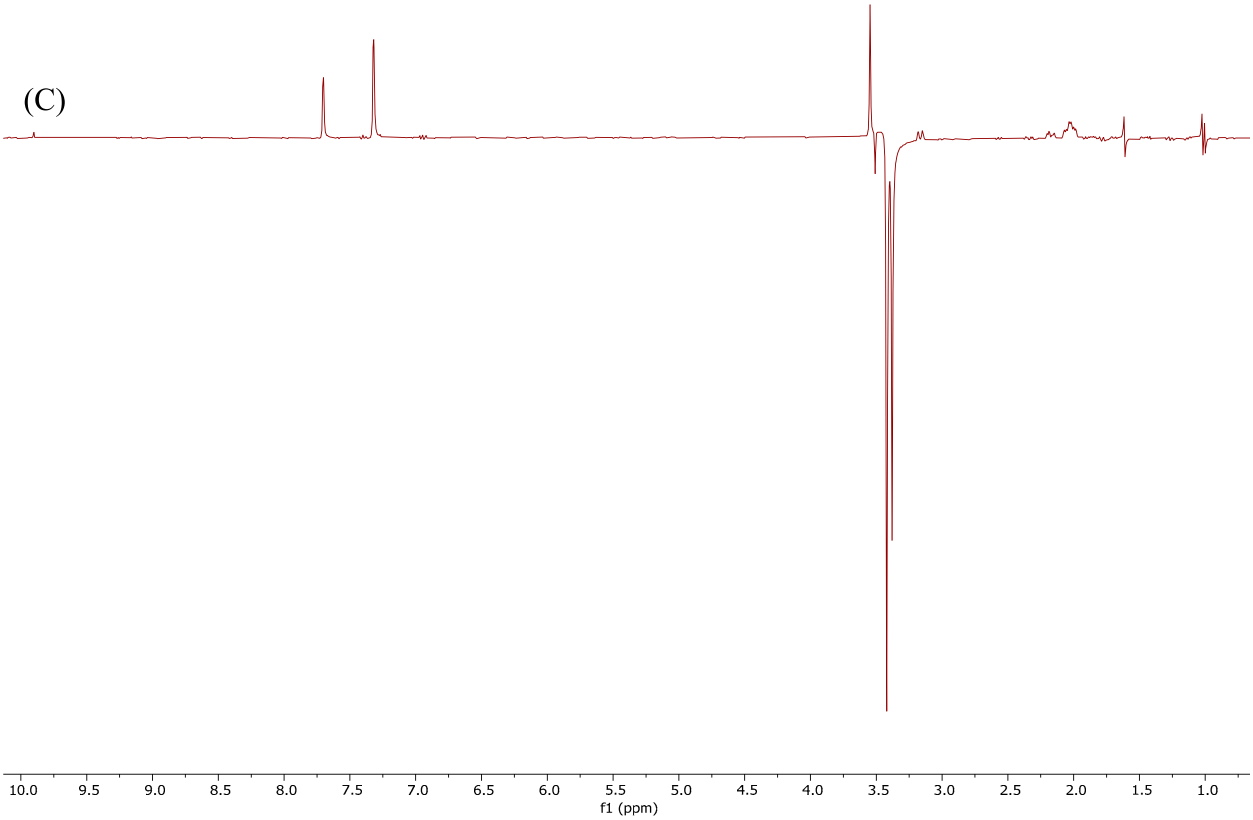

NOESY1D_05

Selective band center: 1.27 (ppm); width: 29.0 (Hz)

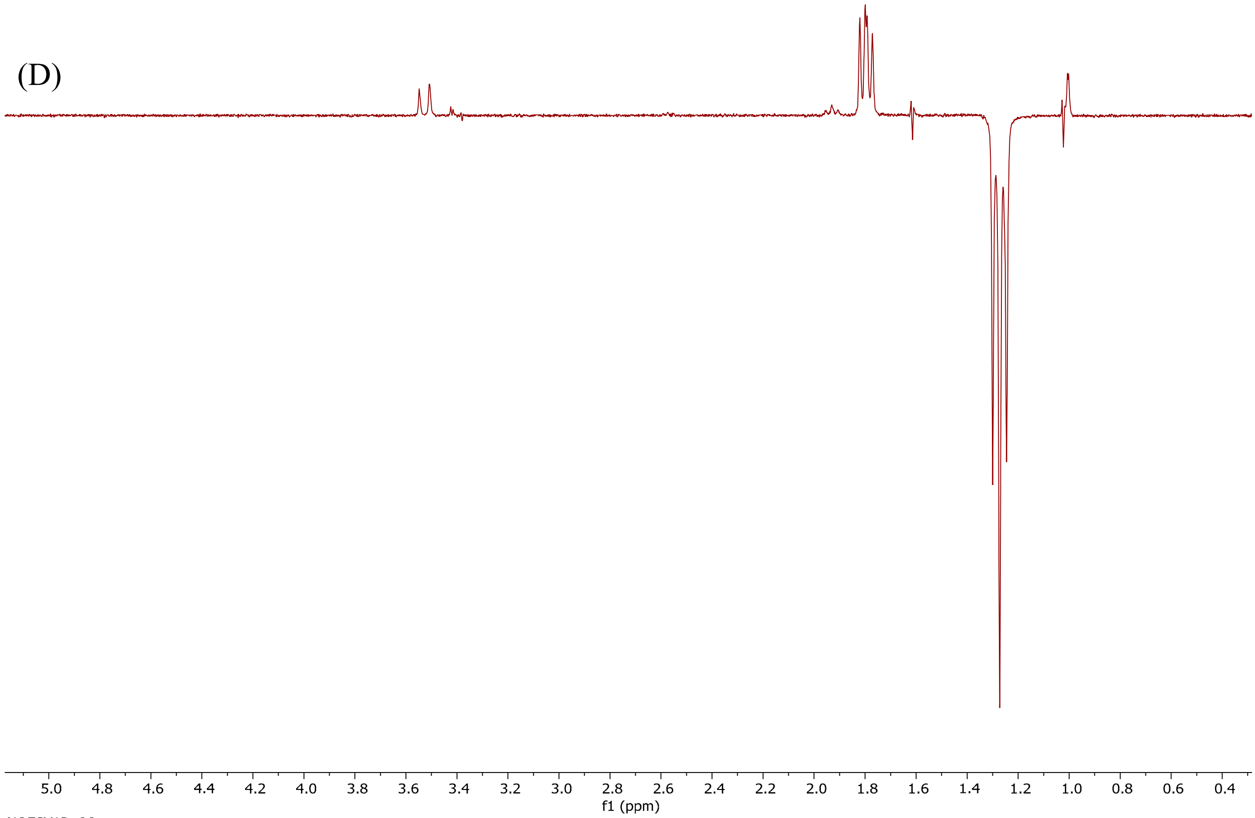

NOESY1D_06

Selective band center: 1.93 (ppm); width: 28.2 (Hz)

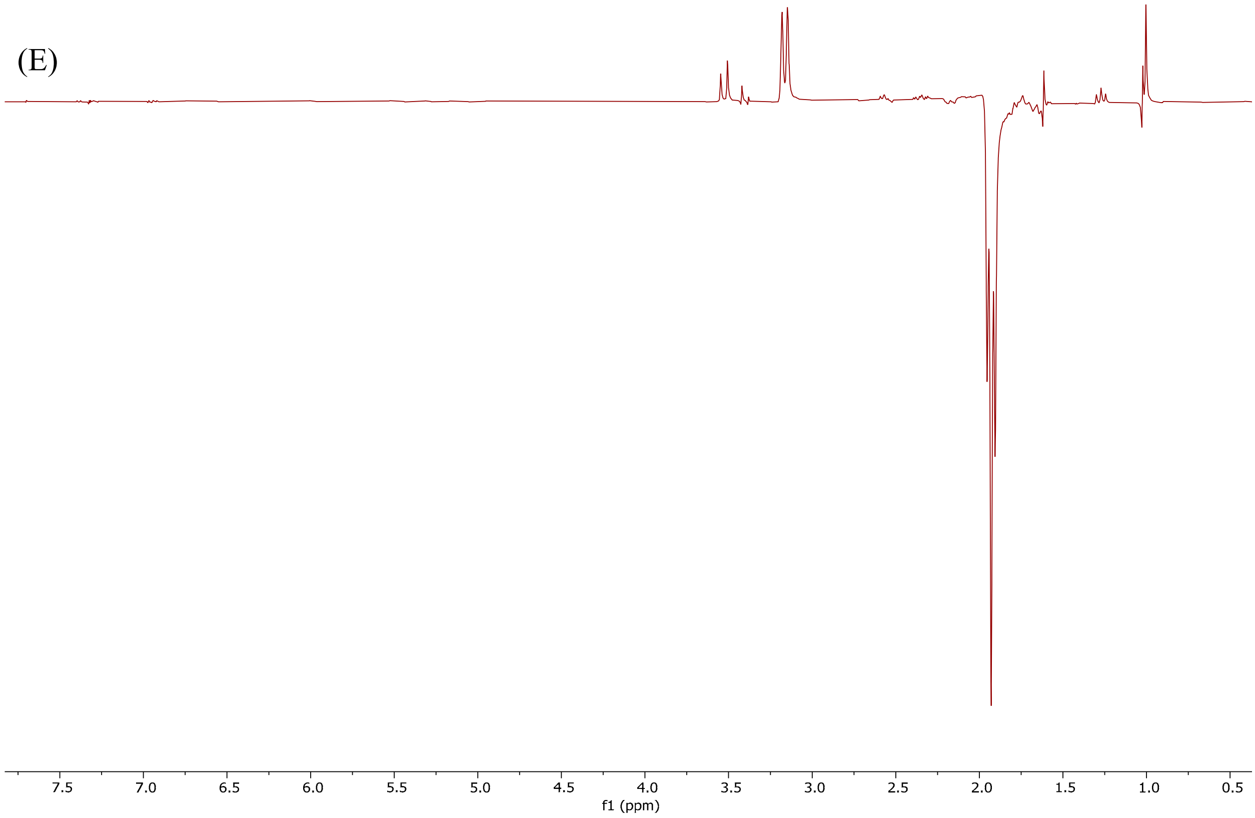

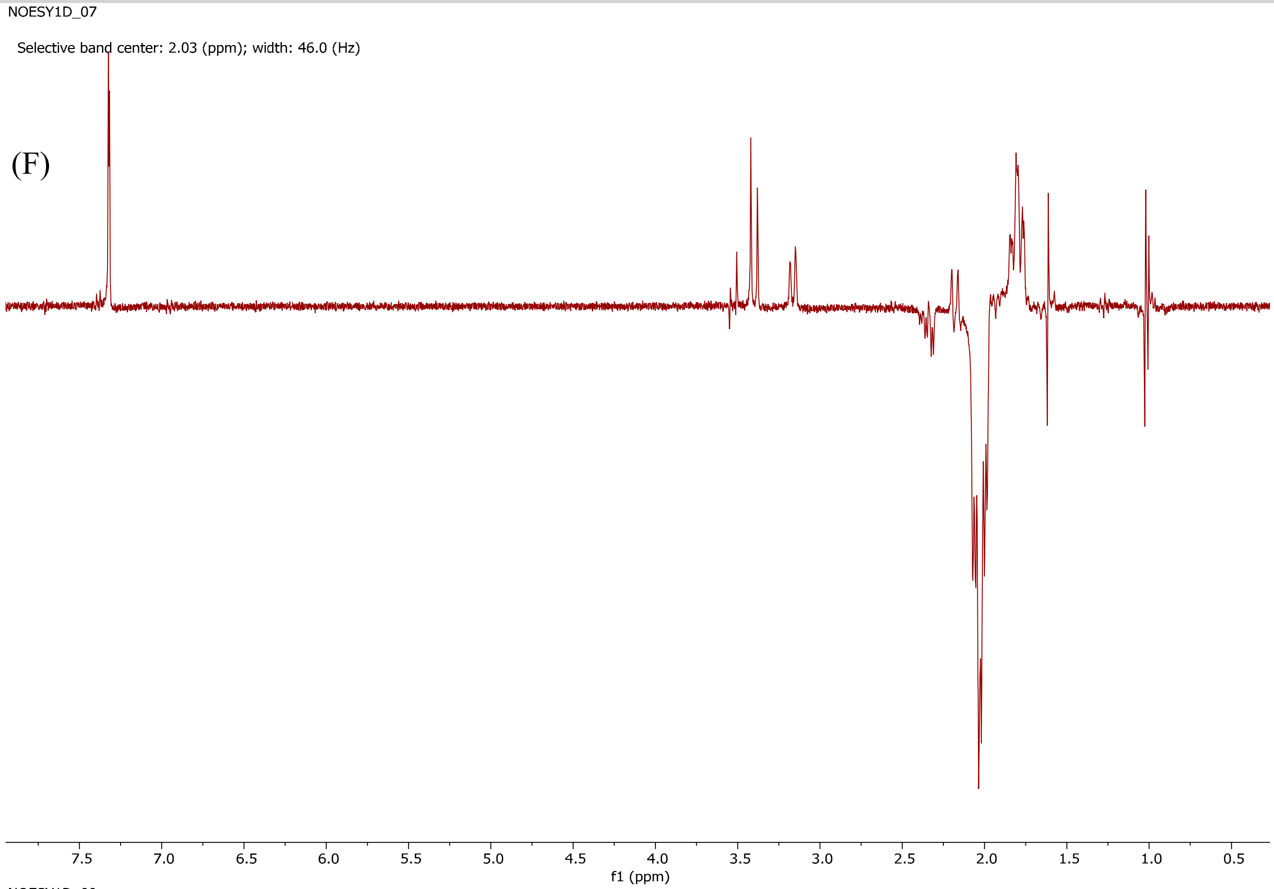

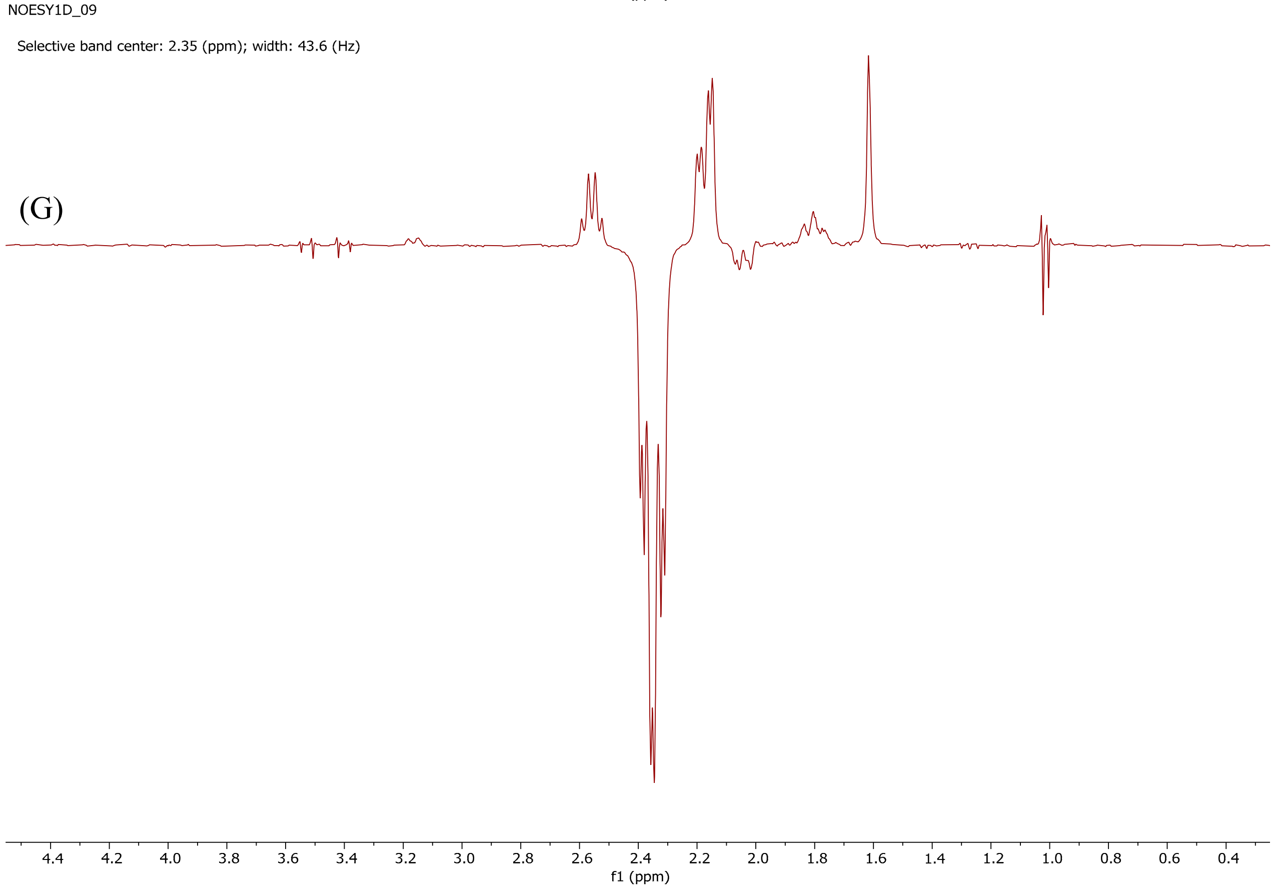

Figure S60. ^{1}H NOESY1D NMR spectra compound **27**.

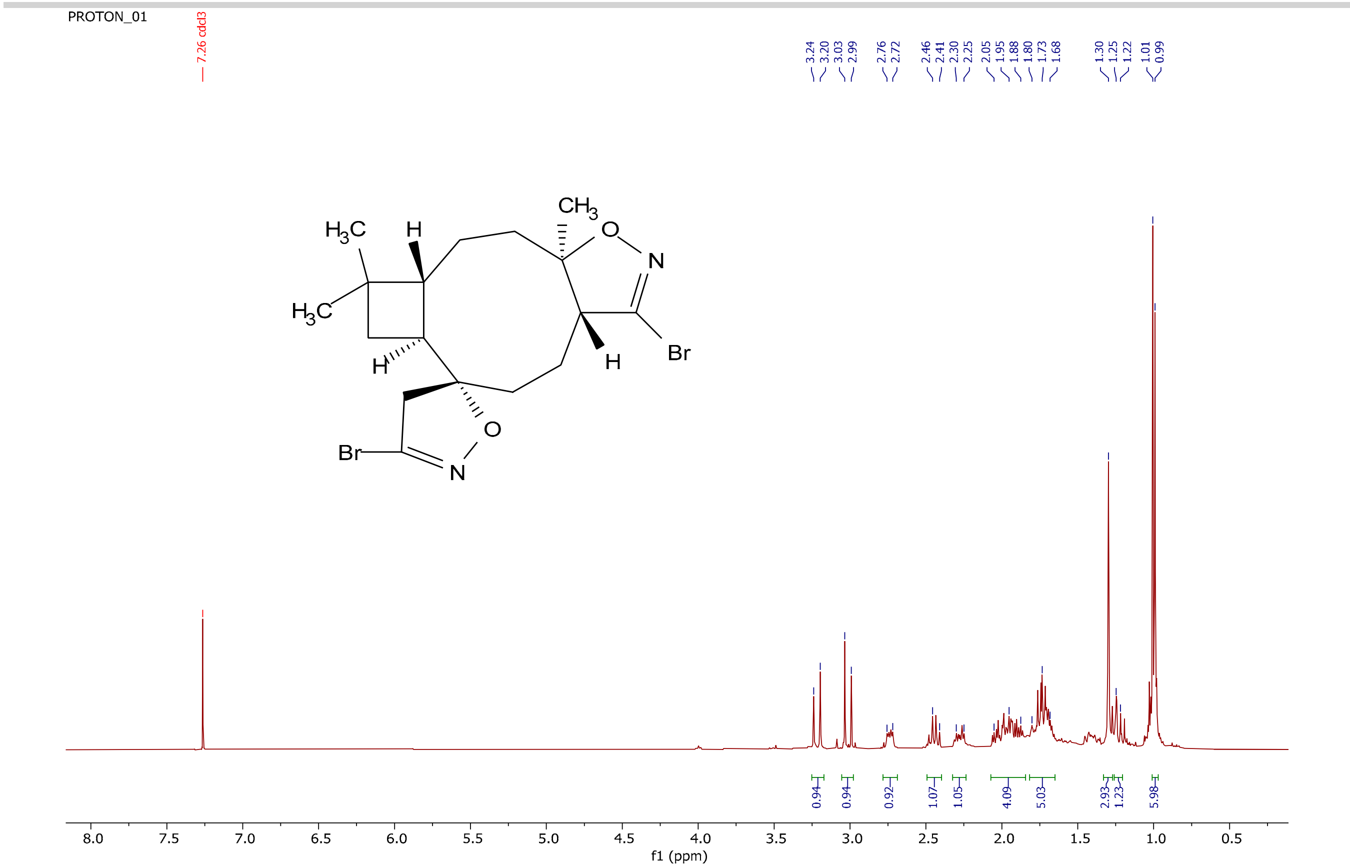

Figure S61. ¹H NMR spectra of compound **28.**

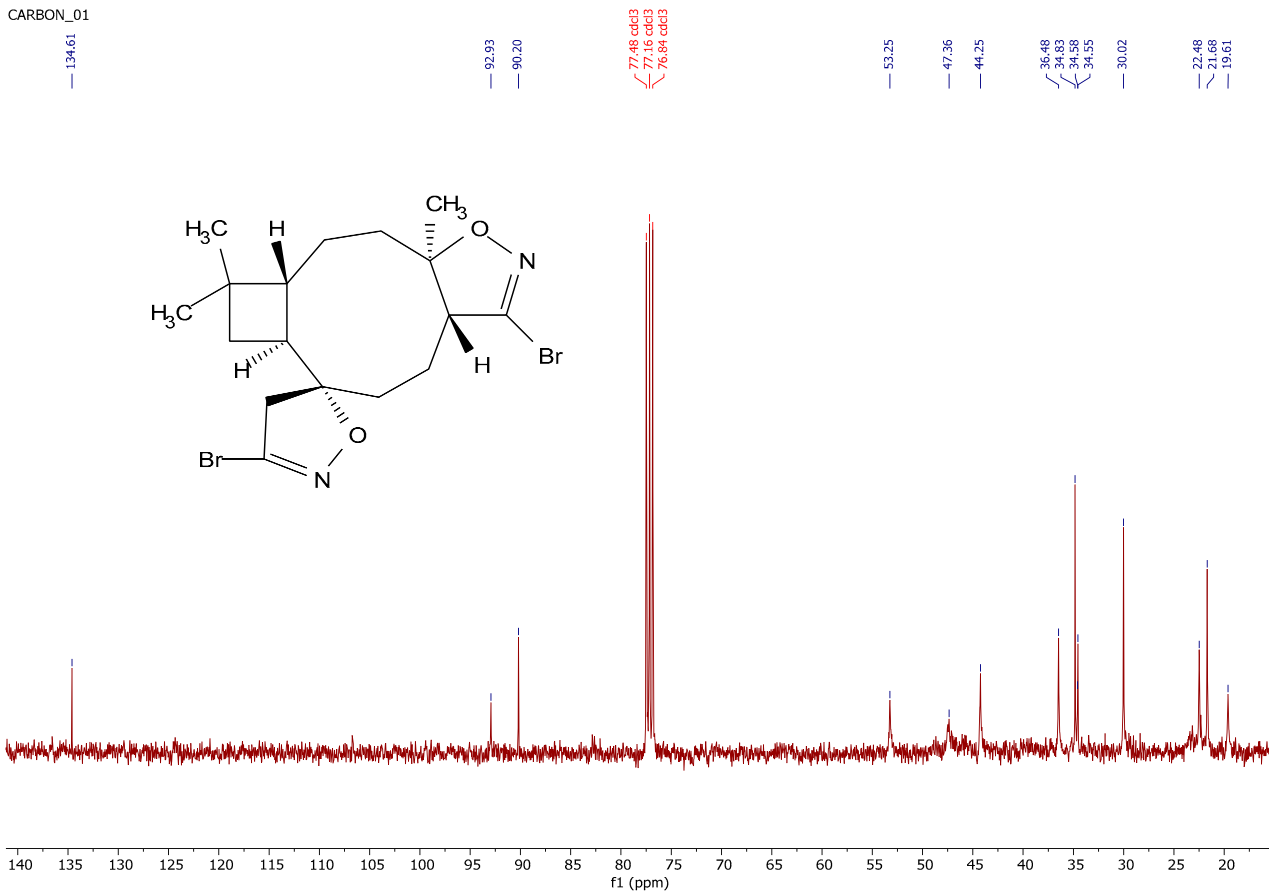

Figure S62. ¹³C NMR spectra of compound **28.**

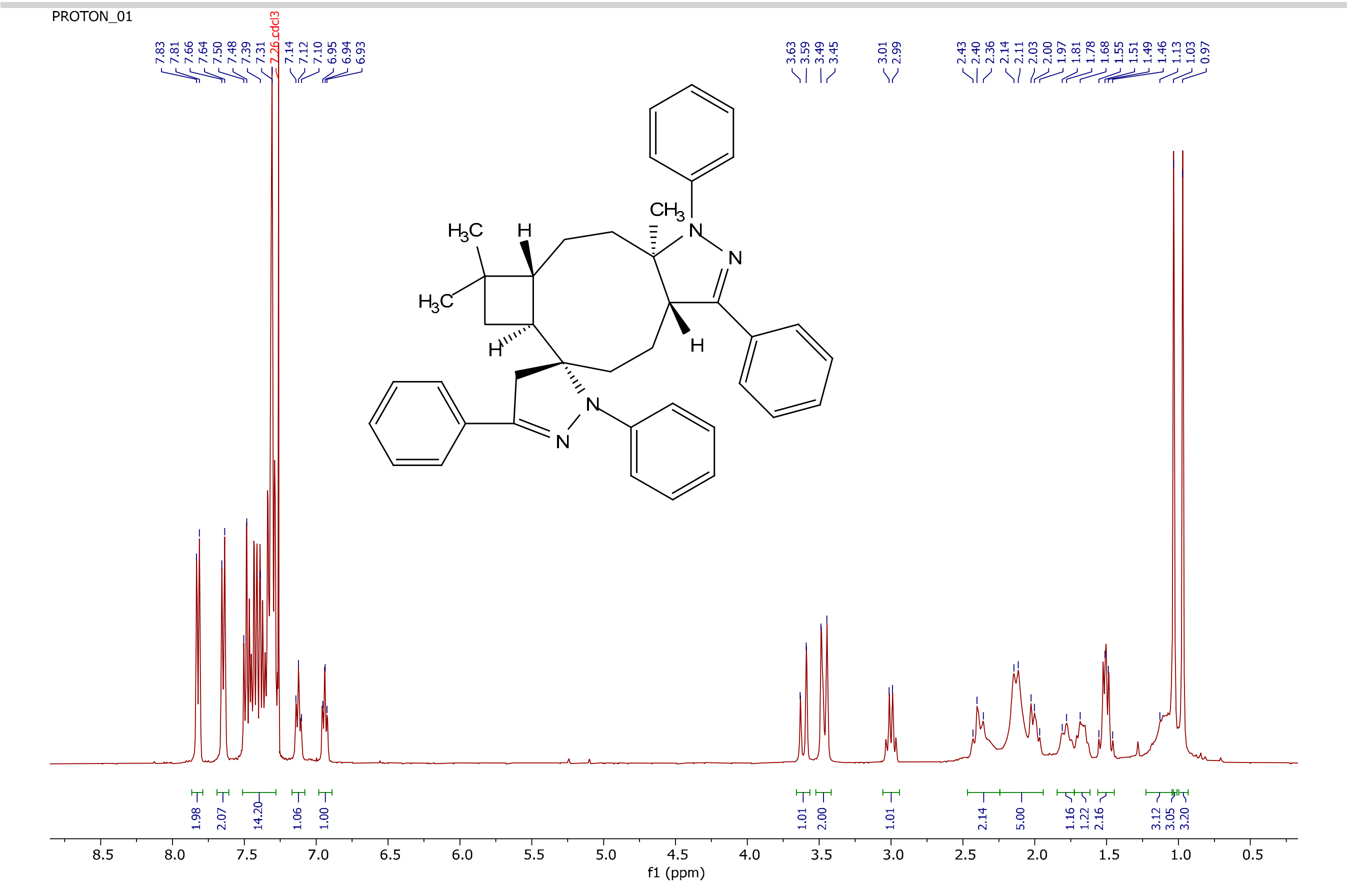

Figure S63. ^{1}H NMR spectra of compound **29.**

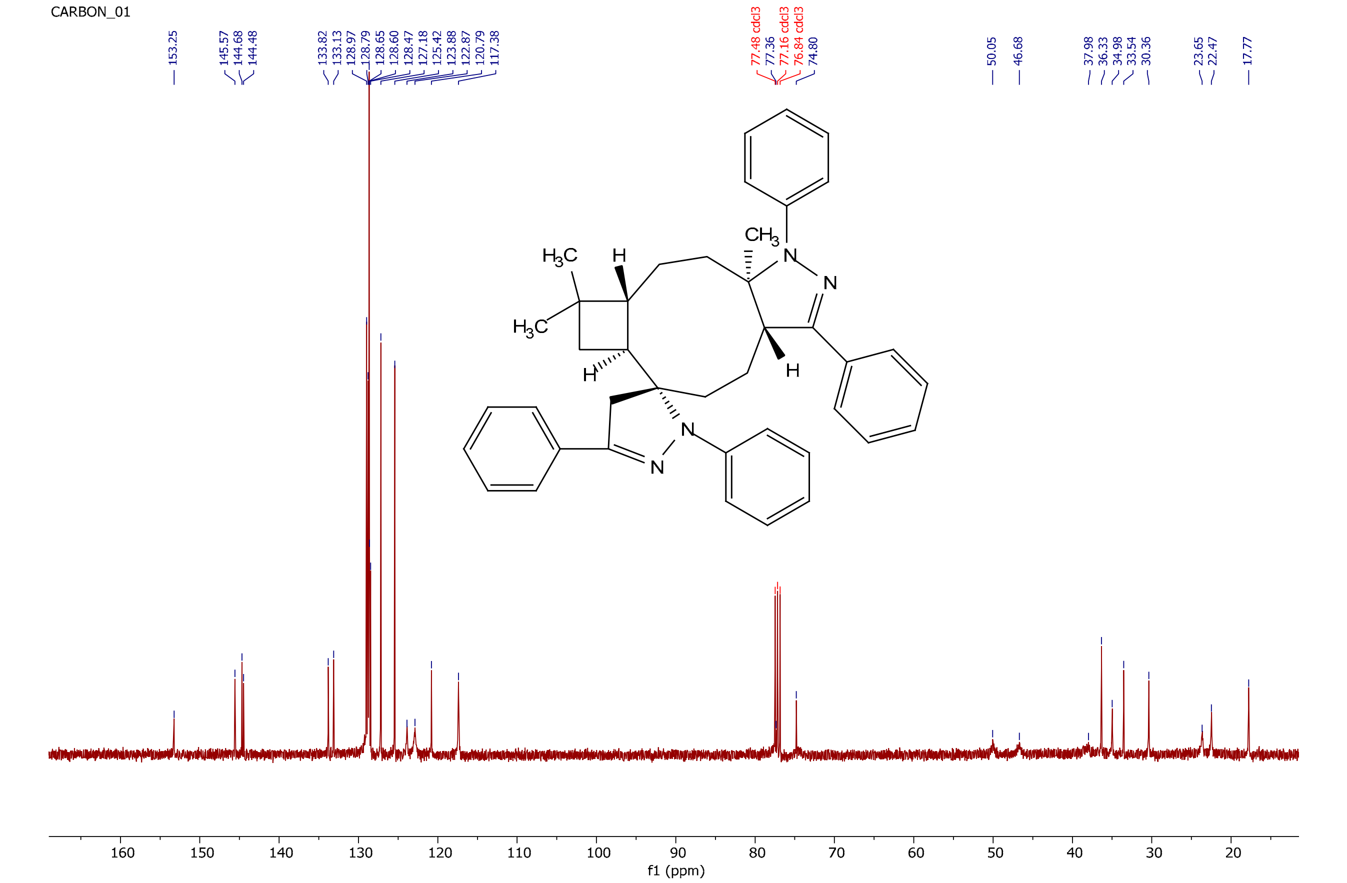

Figure S64. ^{13}C NMR spectra of compound **29.**

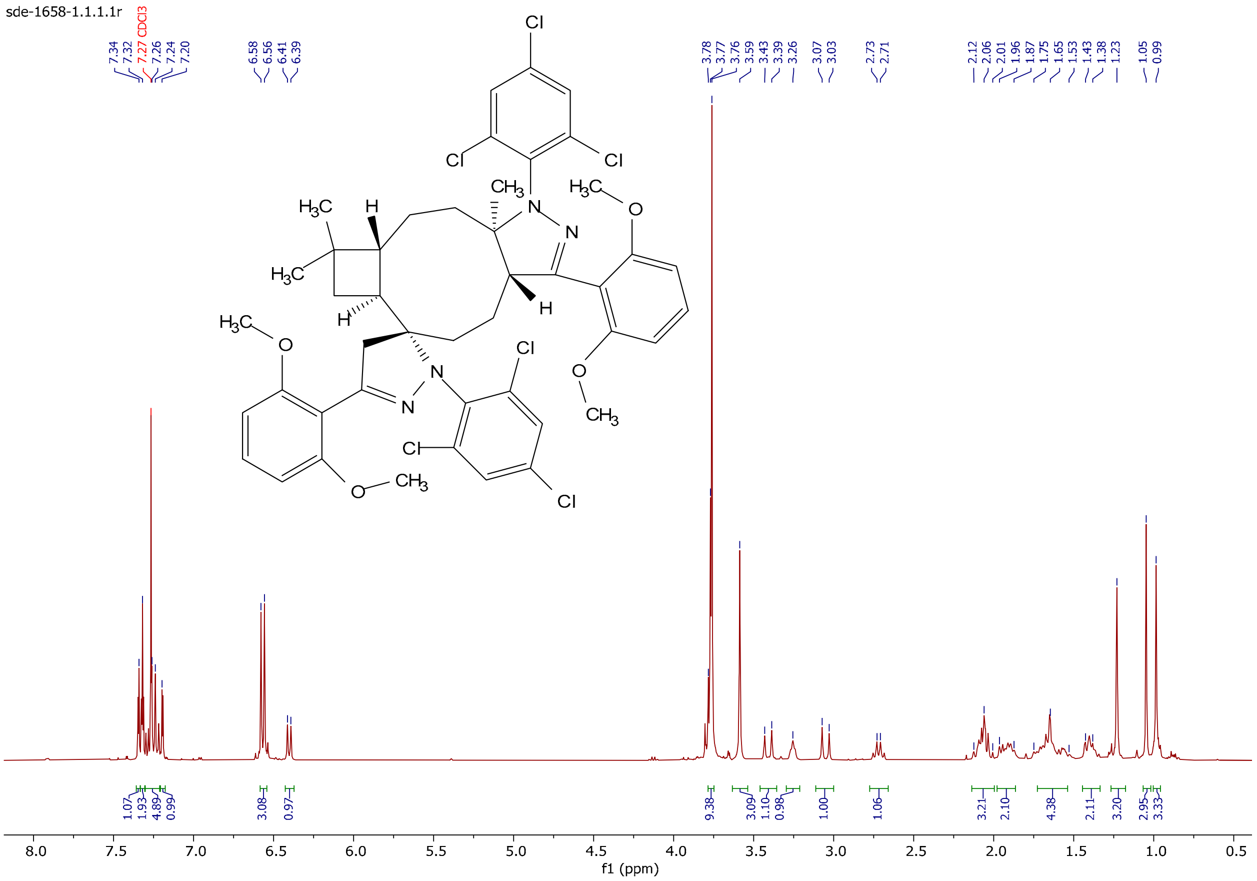

Figure S65. ^{1}H NMR spectra of compound **30.**

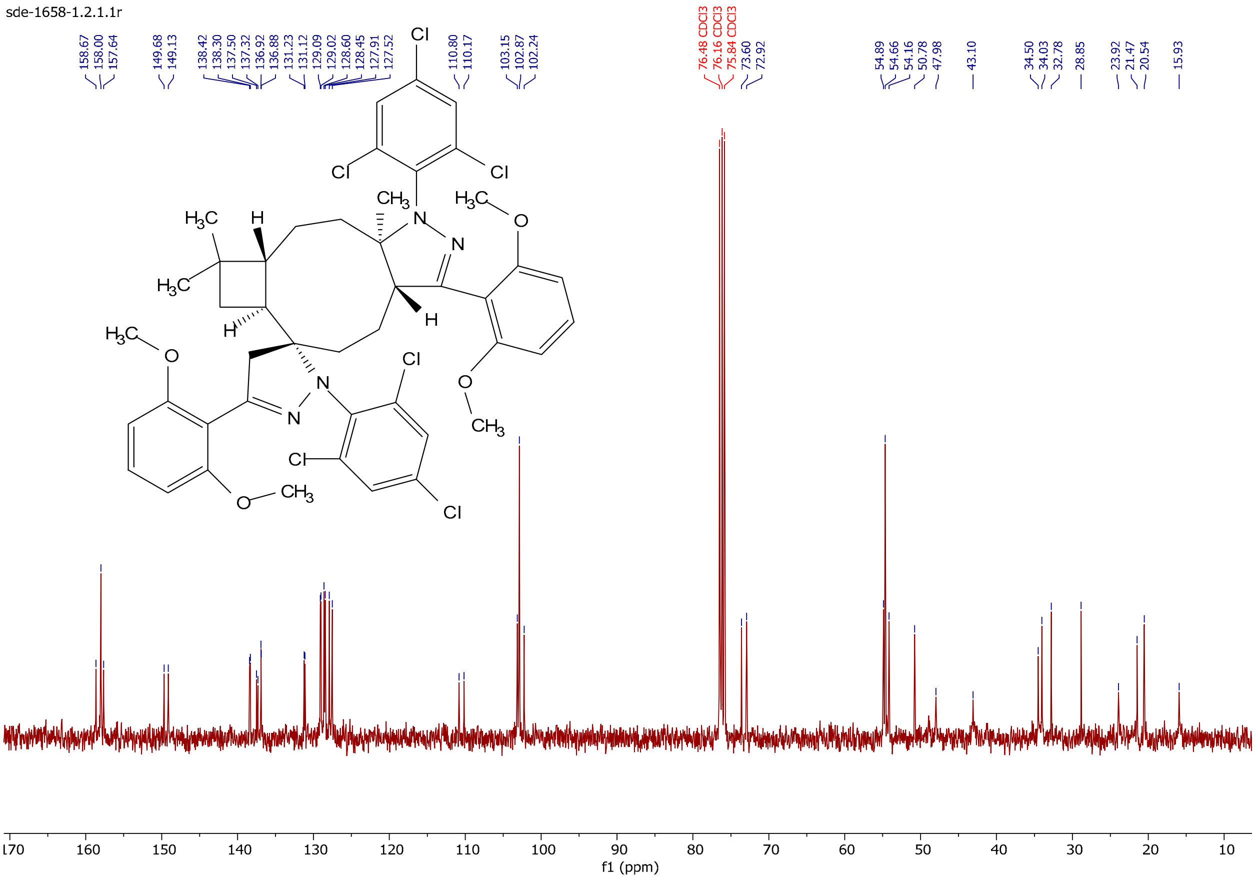

Figure S66. ^{13}C NMR spectra of compound **30.**

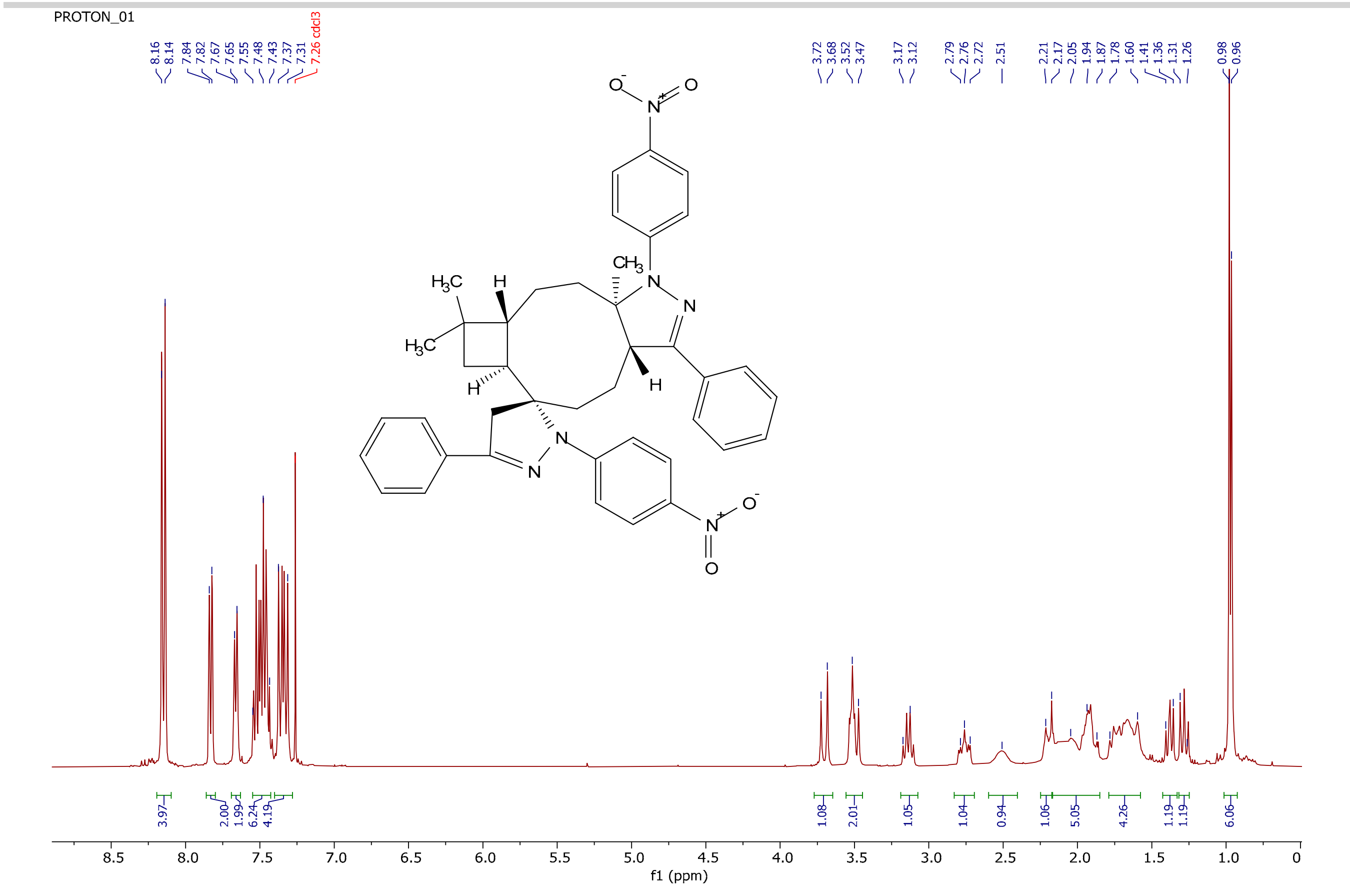

Figure S67. ^{1}H NMR spectra of compound **31.**

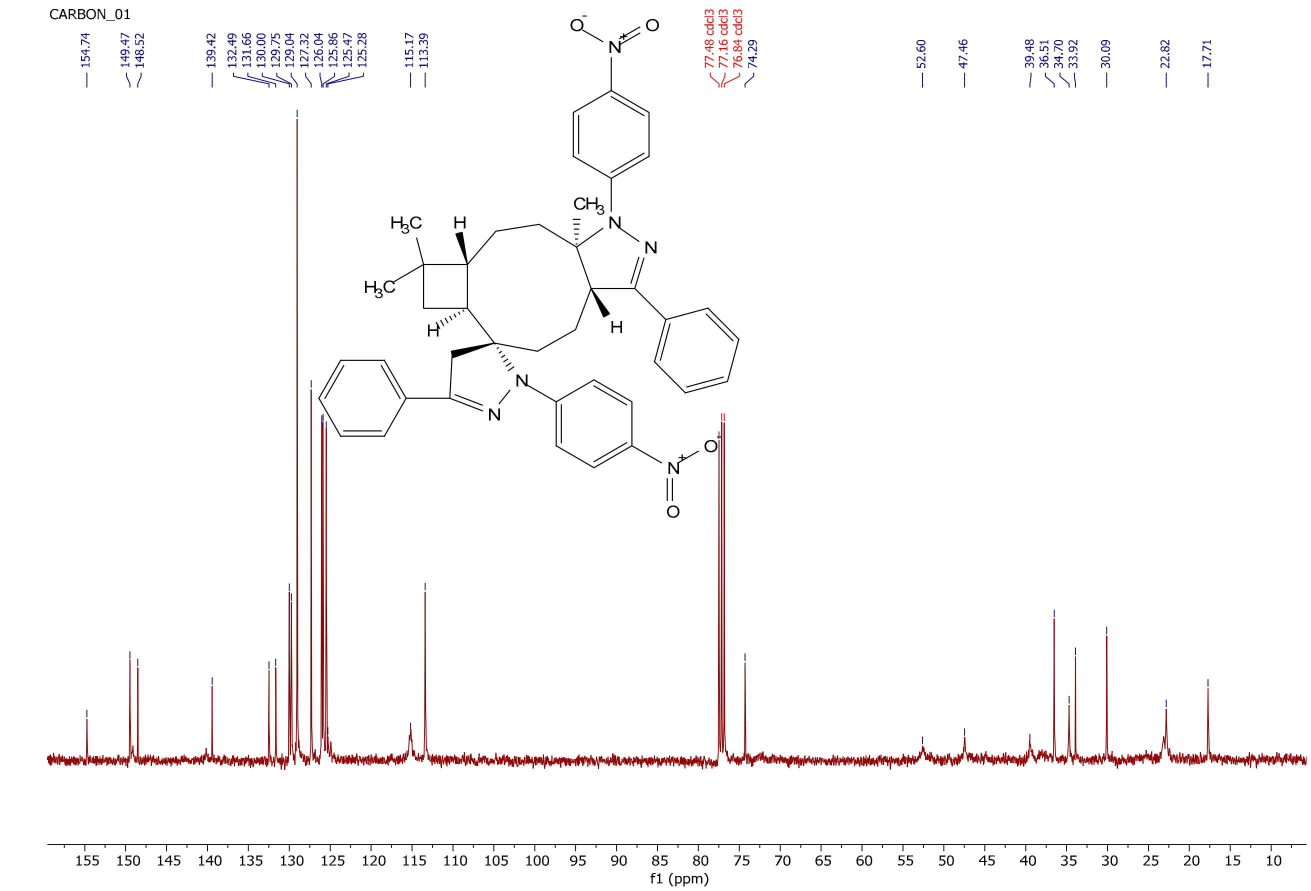

Figure S68. ^{13}C NMR spectra of compound **31.**

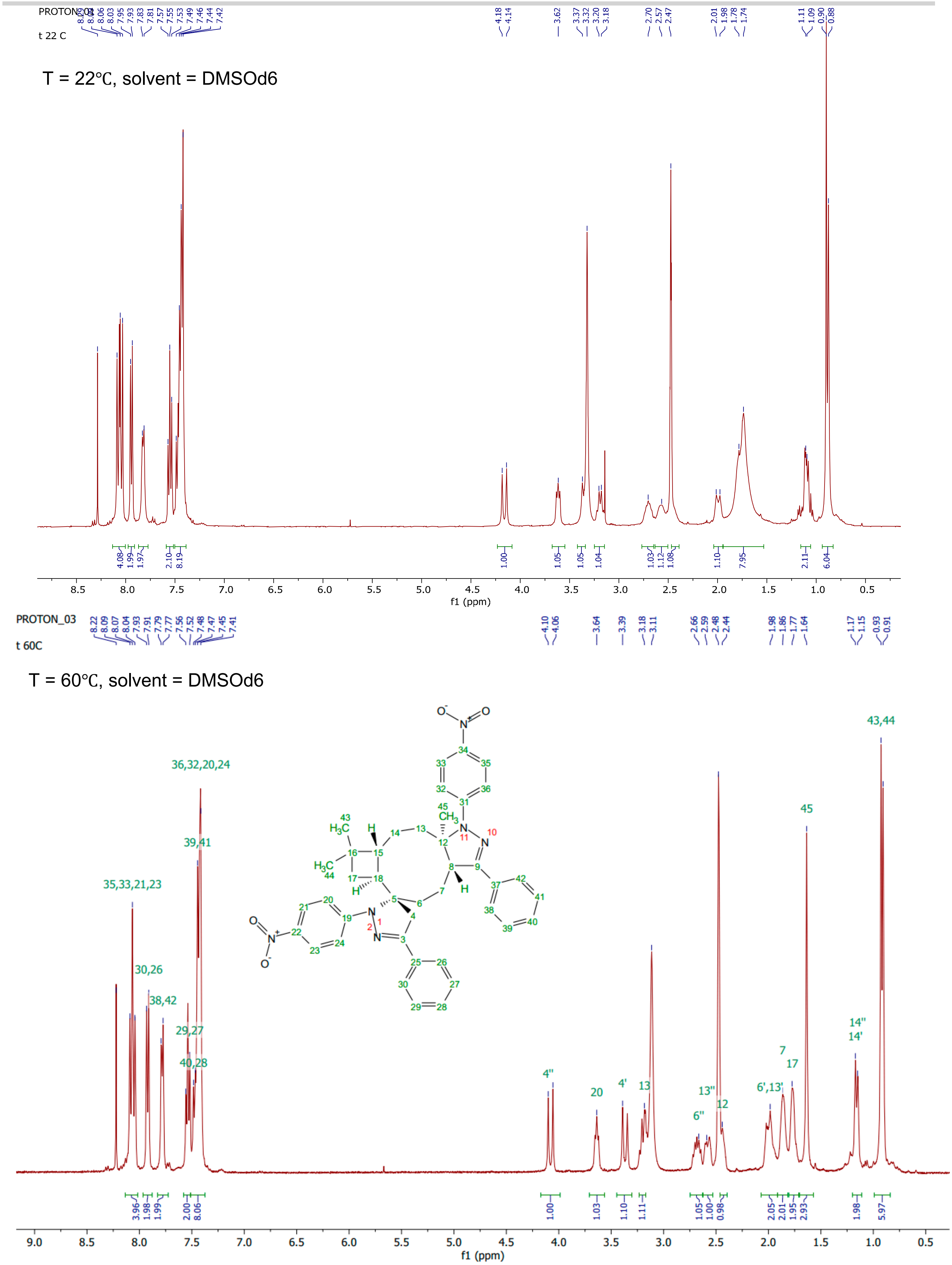

Figure S69. ¹H NMR spectra of compound **31** at different temperatures**.**

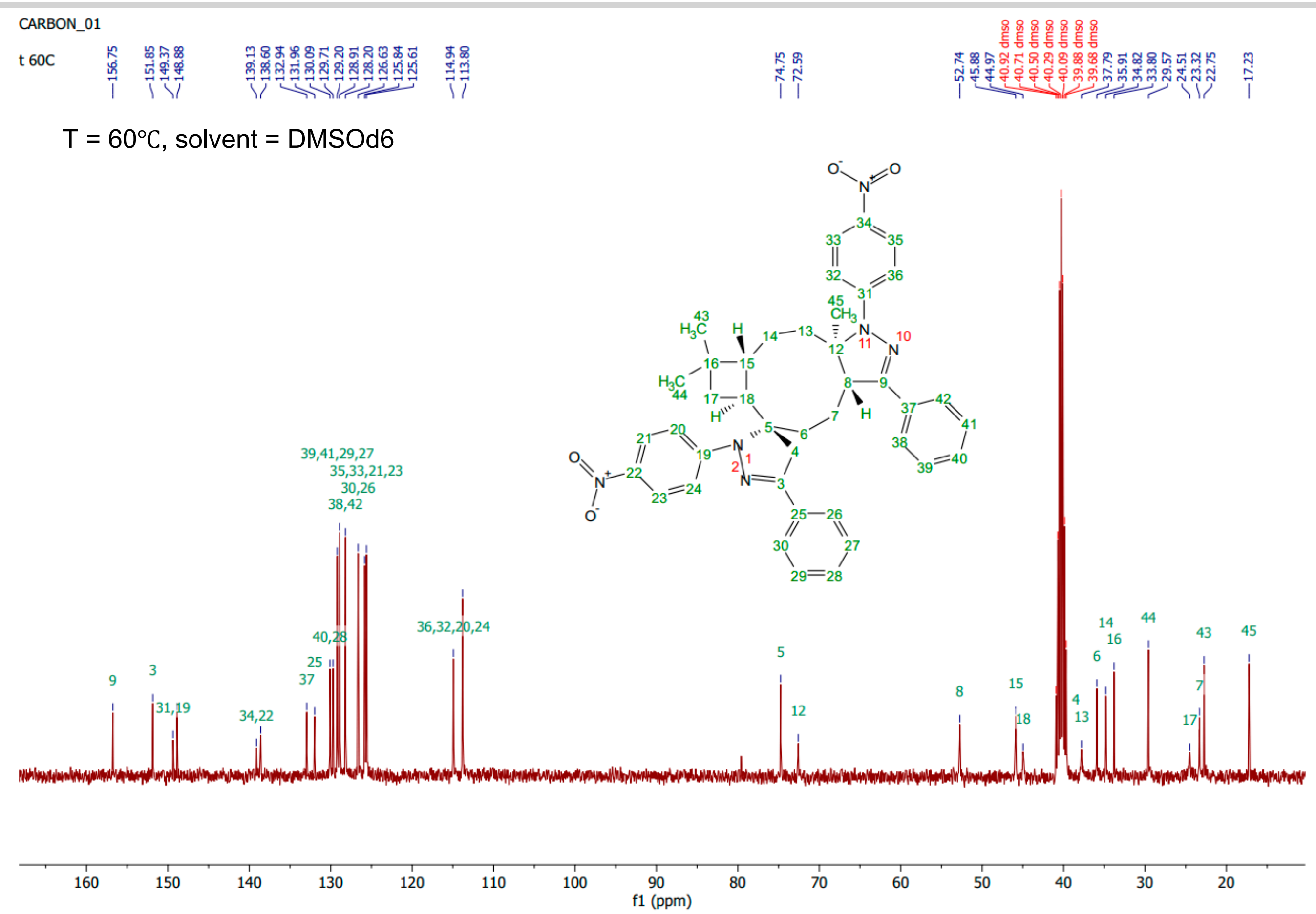

Figure S70. ^{13}C NMR spectra of compound **31** (T = 60℃).

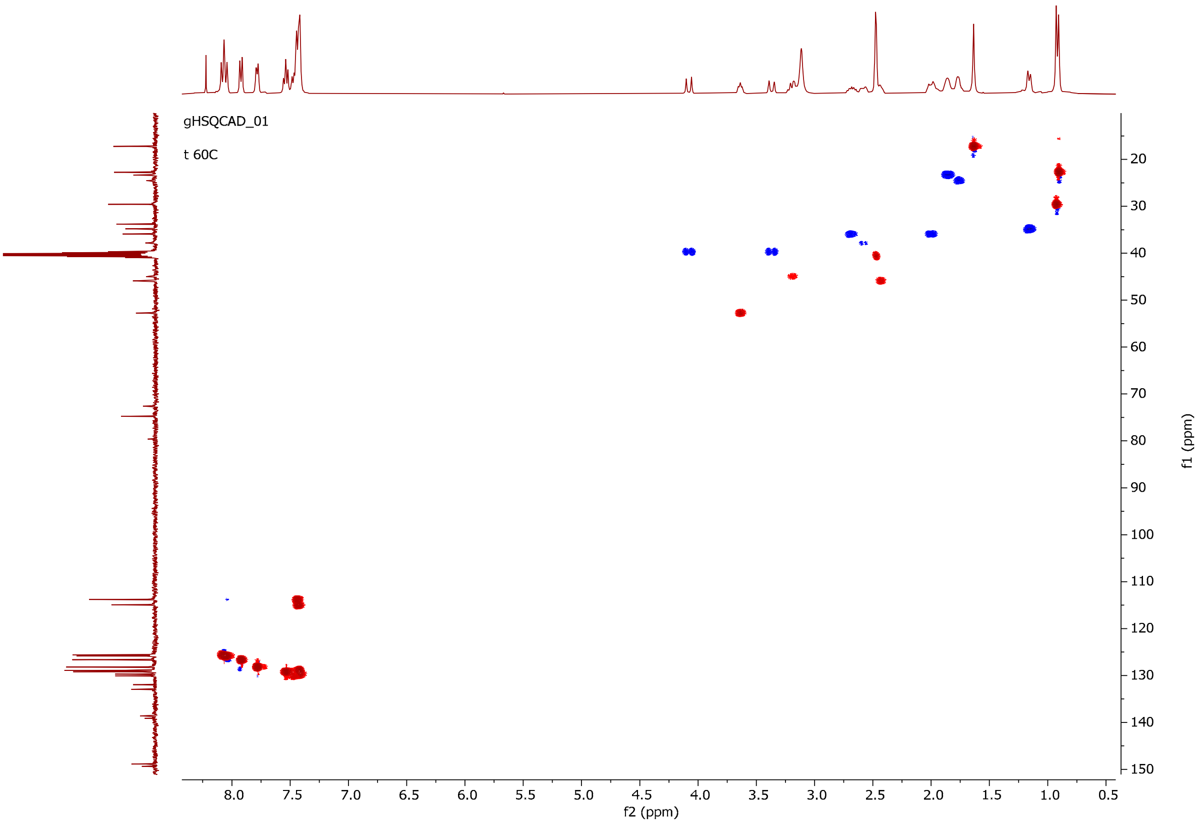

Figure S71. HSQC ^{1}H-^{13}C NMR spectra of compound **31** (T = 60℃).

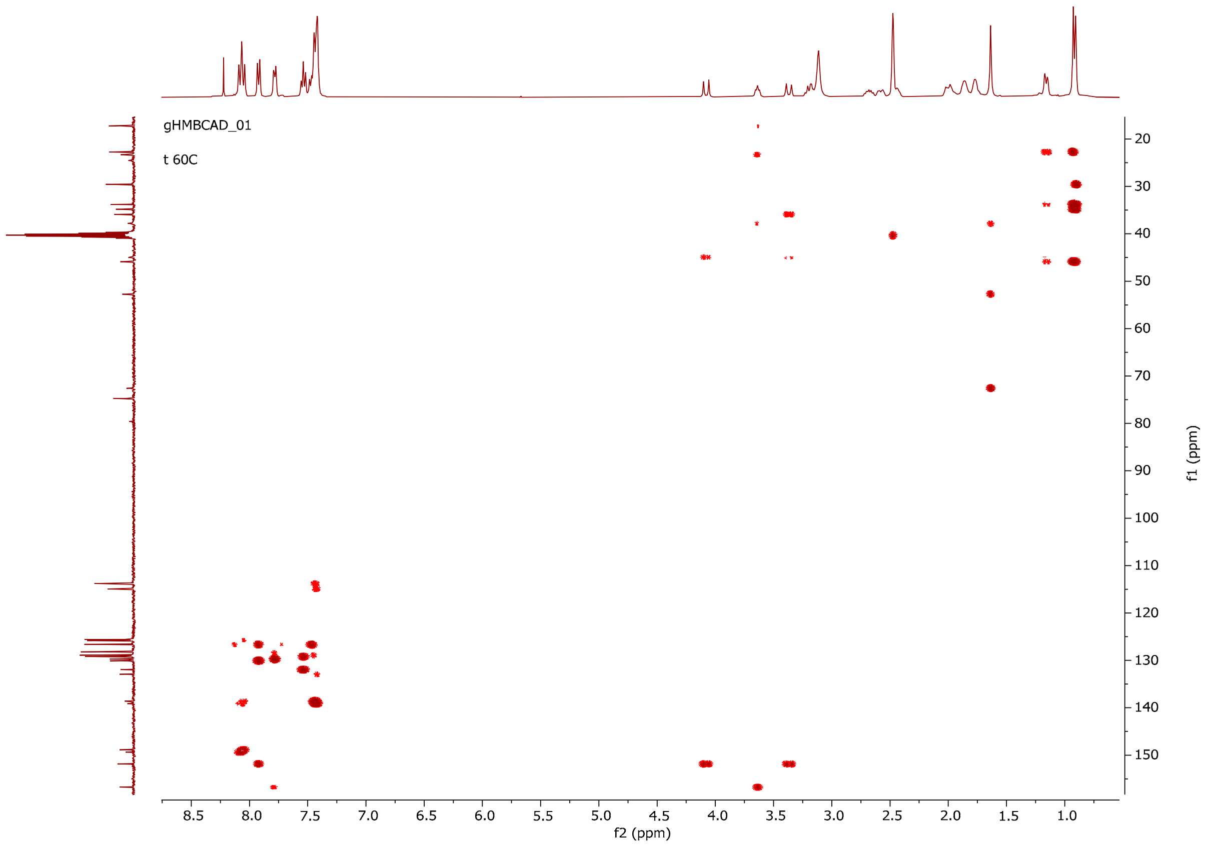

Figure S72. HMBC ^{1}H-^{13}C NMR spectra of compound **31** (T = 60℃).

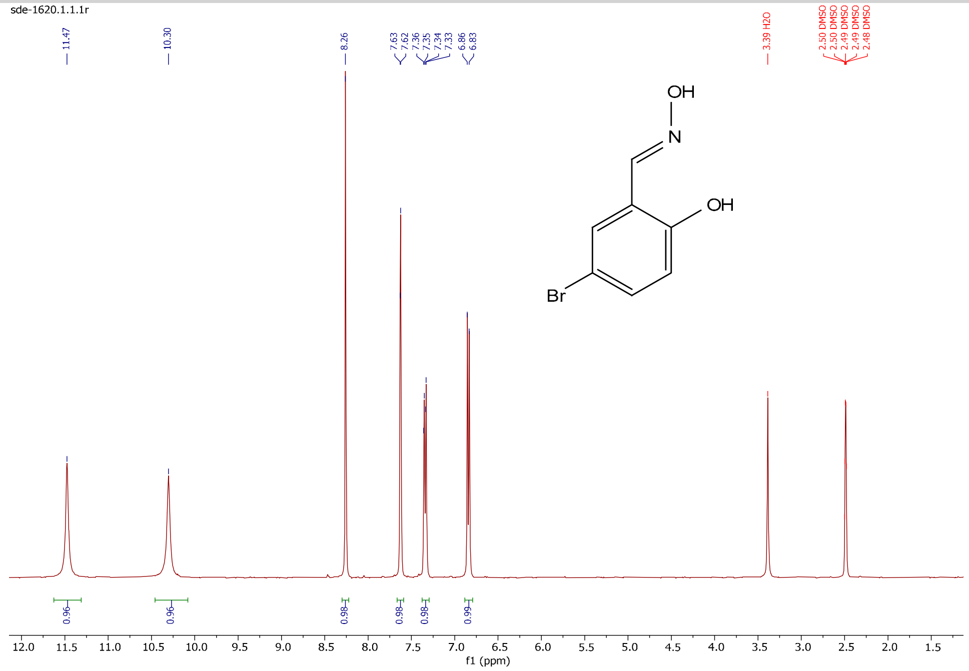

Figure S73. ^{1}H NMR spectra of compound **32.**

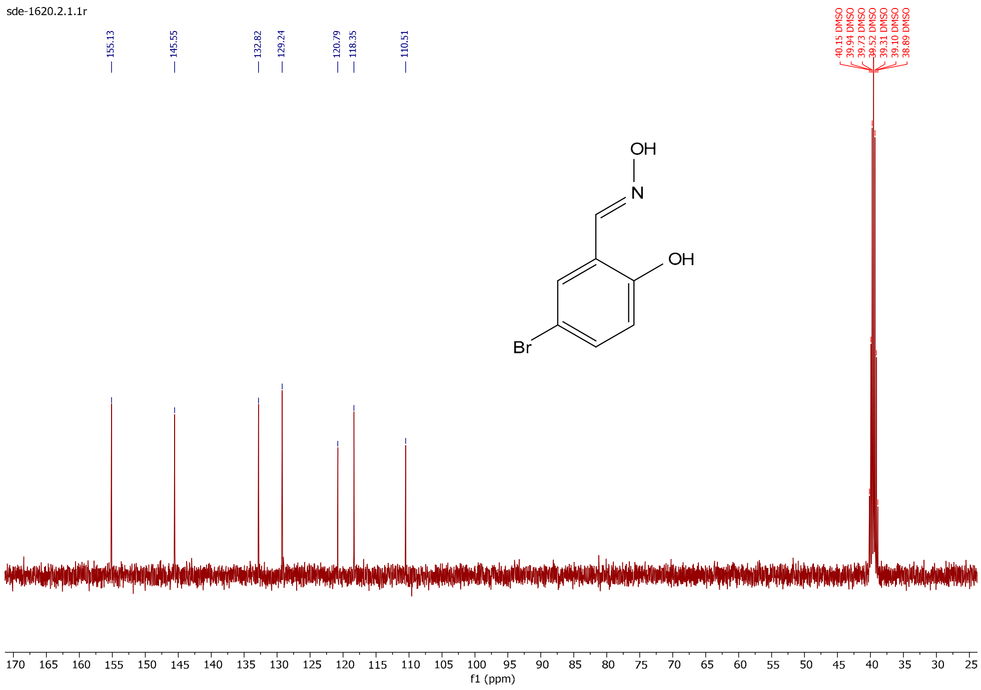

Figure S74. ^{13}C NMR spectra of compound **32.**

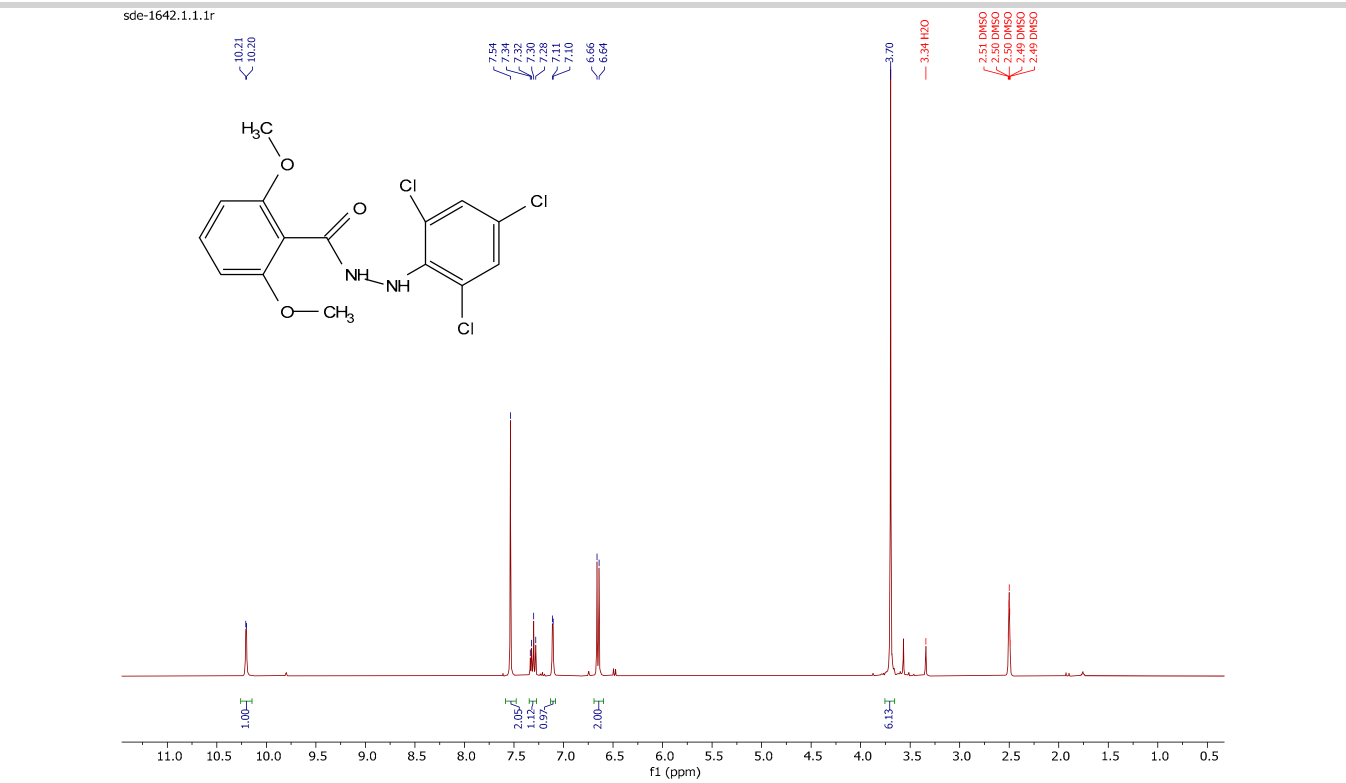

Figure S75. ^{1}H NMR spectra of compound **33.**

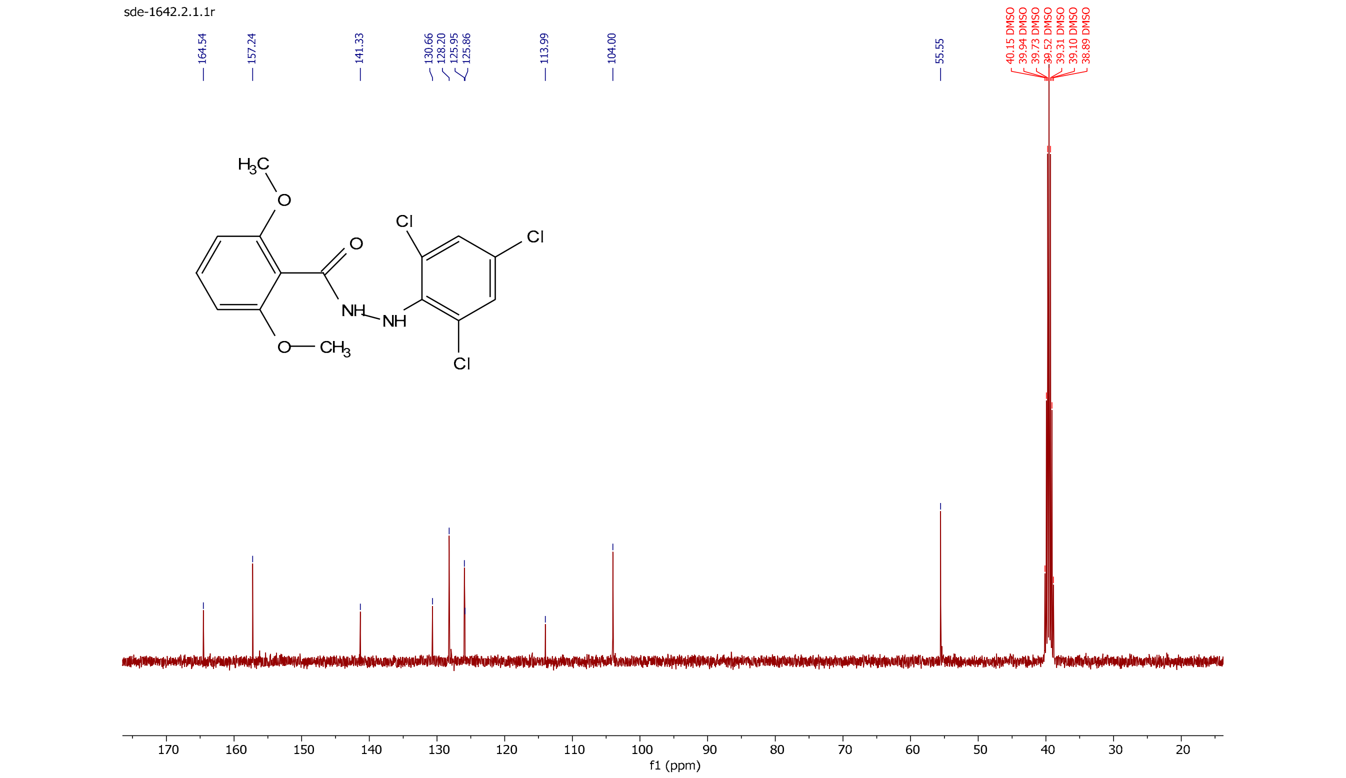

Figure S76. ^{13}C NMR spectra of compound **33.**

Table S1. Crystal data and structure refinement for compound 18a.

Identification code	(18a) sde1619_1)
Empirical formula	C22 H28 Br N O2
Formula weight	418.36
Temperature	295(2) K
Wavelength	1.54186 Å
Crystal system	Orthorhombic
Space group	C 2 2 21
Unit cell dimensions	a = 11.2541(3) Å = 90°.
	b = 15.6197(6) Å = 90°.
	c = 23.7474(9) Å = 90°.
Volume	4174.5(3) E^3
Z	8
Density (calculated)	1.331 Mg/m^3
Absorption coefficient	2.794 mm^{-1}
F(000)	1744
Theta range for data collection	3.723 to 66.596°.
Index ranges	-13<=h<=9, -18<=k<=18, -22<=l<=28
Reflections collected	14857
Independent reflections	3653 [R(int) = 0.0692]
Completeness to theta = 66.596°	99.8 %
Refinement method	Full-matrix least-squares on F^2
Data / restraints / parameters	3653 / 0 / 243
Goodness-of-fit on F^2	0.960
Final R indices [I>2sigma(I)]	R1 = 0.0413, wR2 = 0.0977
R indices (all data)	R1 = 0.0570, wR2 = 0.1025
Absolute structure parameter	-0.038(18)
Extinction coefficient	0.00069(9)
Largest diff. peak and hole	0.329 and -0.369 e. $Å^{-3}$

Table S2. Hydrogen bonds for 18a [Å and °].

D-H...A	d(D-H)	d(H...A)	d(D...A)	<(DHA)
O(2)-H(2)...N(1)	0.79(8)	1.88(8)	2.615(7)	155(7)

Symmetry transformations used to generate equivalent atoms:

Table S3. Crystal data and structure refinement for compound 21a.

Identification code	21a	
Empirical formula	C23 H32 N2	
Formula weight	336.50	
Temperature	295(2) K	
Wavelength	1.54186 Å	
Crystal system	Monoclinic	
Space group	P 21	
Unit cell dimensions	a = 8.3636(5) Å	= 90°.
	b = 21.3520(10) Å	= 91.916(5)°.
	c = 11.436 Å	= 90°.
Volume	2041.15(16) Å^3	
Z	4	
Density (calculated)	1.095 Mg/m^3	
Absorption coefficient	0.477 mm^{-1}	
F(000)	736	
Theta range for data collection	4.141 to 66.521°.	
Index ranges	-9<=h<=9, -25<=k<=25, -3<=l<=13	
Reflections collected	13382	
Independent reflections	6437 [R(int) = 0.1363]	
Completeness to theta = 66.521°	95.4 %	
Refinement method	Full-matrix least-squares on F^2	
Data / restraints / parameters	6437 / 1 / 464	
Goodness-of-fit on F^2	0.756	
Final R indices [I>2sigma(I)]	R1 = 0.0590, wR2 = 0.1278	
R indices (all data)	R1 = 0.1580, wR2 = 0.1482	
Absolute structure parameter	-1.3(10)	
Extinction coefficient	0.0047(5)	
Largest diff. peak and hole	0.191 and -0.223 e.Å^{-3}	

Table S4. Crystal data and structure refinement for compound 21b

Identification code	21b (sde1516_1)	
Empirical formula	C23 H32 N2	
Formula weight	336.50	
Temperature	295(2) K	
Wavelength	1.54186 Å	
Crystal system	Orthorhombic	
Space group	P 21 21 21	
Unit cell dimensions	a = 7.0538(6) Å	a= 90°.
	b = 11.7944(9) Å	b= 90°.
	c = 23.4880(10) Å	g = 90°.
Volume	1954.1(2) Å ^{3}	
Z	4	
Density (calculated)	1.144 Mg/m^3	
Absorption coefficient	0.498 mm^{-1}	
F(000)	736	
Theta range for data collection	5.315 to 67.007°.	
Index ranges	-8<=h<=5, -14<=k<=13, -27<=l<=27	
Reflections collected	18687	
Independent reflections	3394 [R(int) = 0.0466]	
Completeness to theta = 67.008°	98.3 %	
Refinement method	Full-matrix least-squares on F^2	
Data / restraints / parameters	3394 / 0 / 251	
Goodness-of-fit on F^2	0.890	
Final R indices [I>2sigma(I)]	R1 = 0.0300, wR2 = 0.0611	
R indices (all data)	R1 = 0.0442, wR2 = 0.0647	
Absolute structure parameter	-0.4(3)	
Extinction coefficient	0.0076(4)	
Largest diff. peak and hole	0.108 and -0.091 e. Å $^{-3}$	

Table S5. Cytotoxic effect of compounds 17a (SDE-1525), 21a (SDE-1558-1), 23a (SDE-1615-1), and 25 (SDE-1650-2) on a variety of cell lines, including colon cancer (HCT116, HT-29), breast cancer (MCF7, SKBR3), melanoma (SK-MEL-28), lung cancer (A549), prostate cancer (DU145), and normal cell (HEK-293), with IC_{50} values.

<table>
<tr><th>Organ</th><th>Cell lines</th><th>Curves in vitro study with IC50 values</th></tr>
<tr><td rowspan="2">Colon cancer</td><td>HCT116</td><td>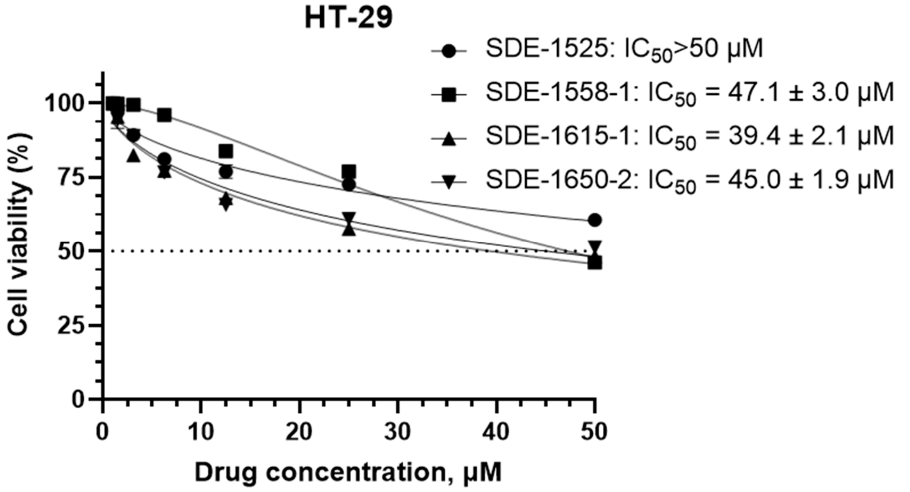
</td></tr>
<tr><td>HT-29</td><td>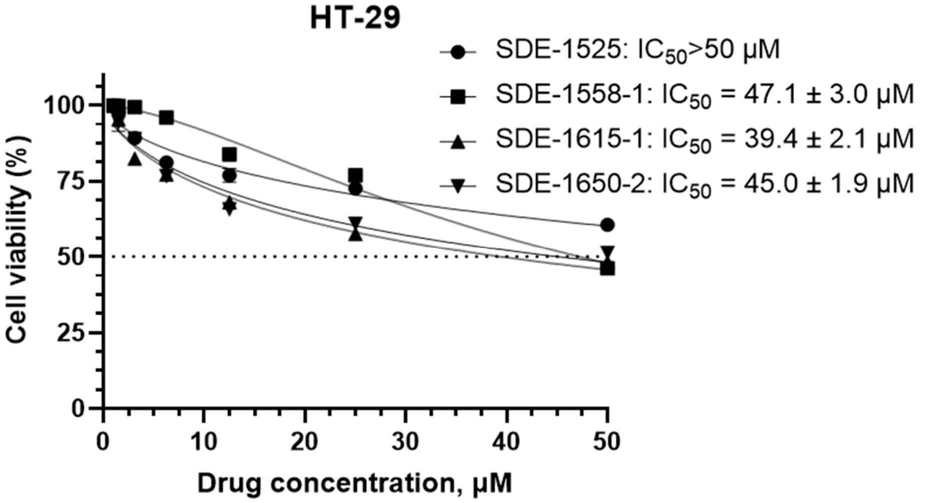
</td></tr>
</table>

<table>
<tr><td rowspan="2">Breast cancer</td><td>MCF7</td><td>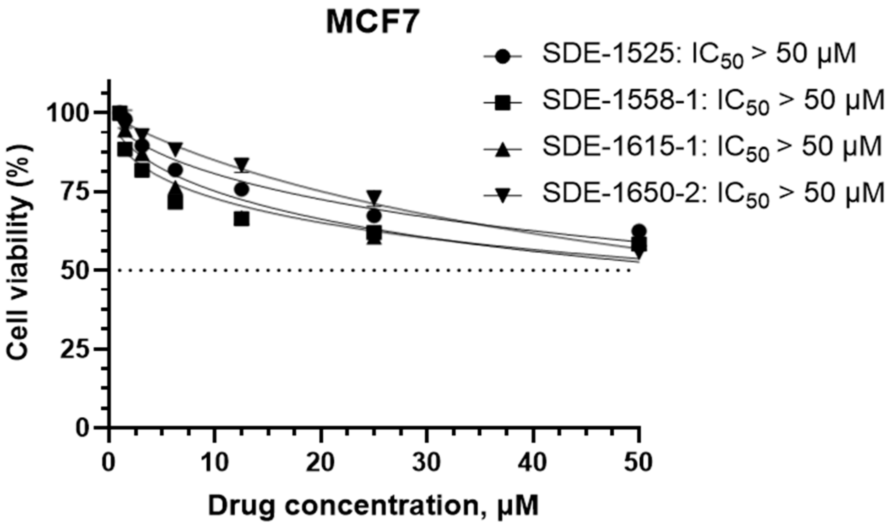
</td></tr>
<tr><td>SKBR3</td><td>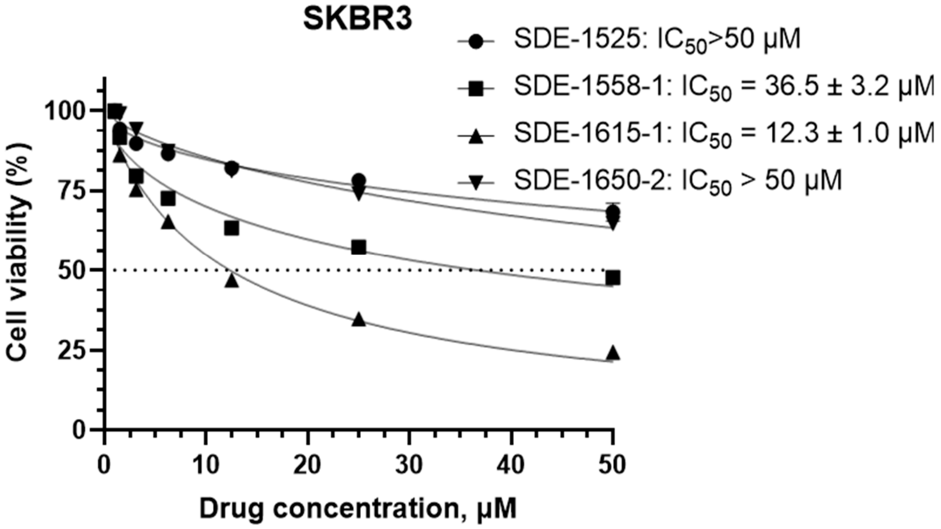
</td></tr>
<tr><td>Melanoma</td><td>SK-MEL-28</td><td>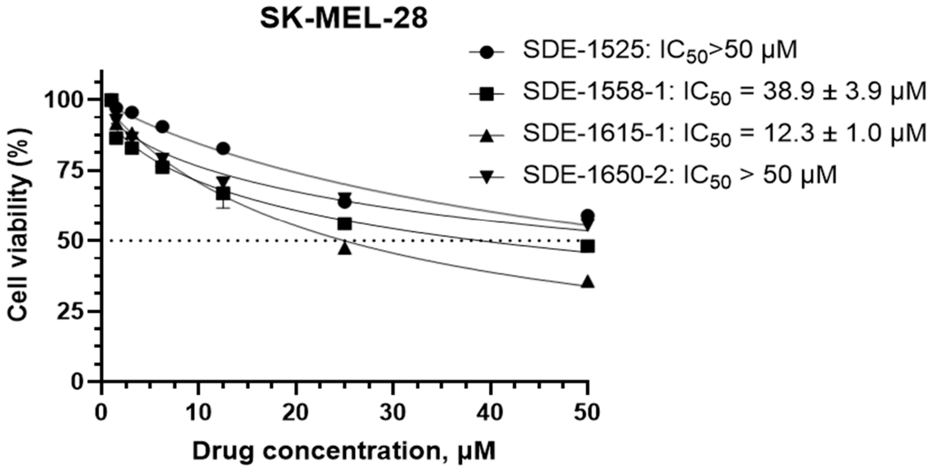
</td></tr>
</table>

<table>
<tr><td>Lung cancer</td><td>A549</td><td>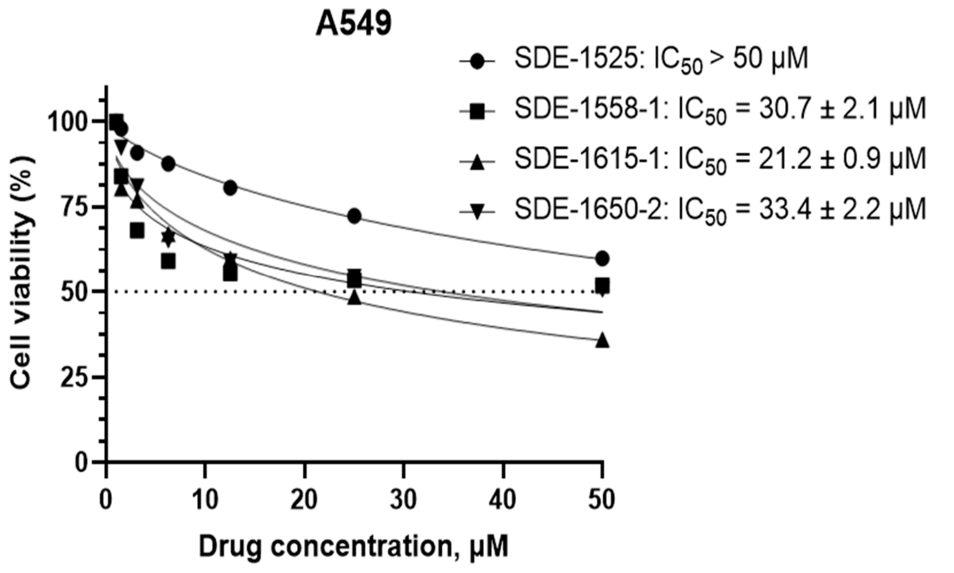
</td></tr>
<tr><td>Prostate cancer</td><td>DU145</td><td>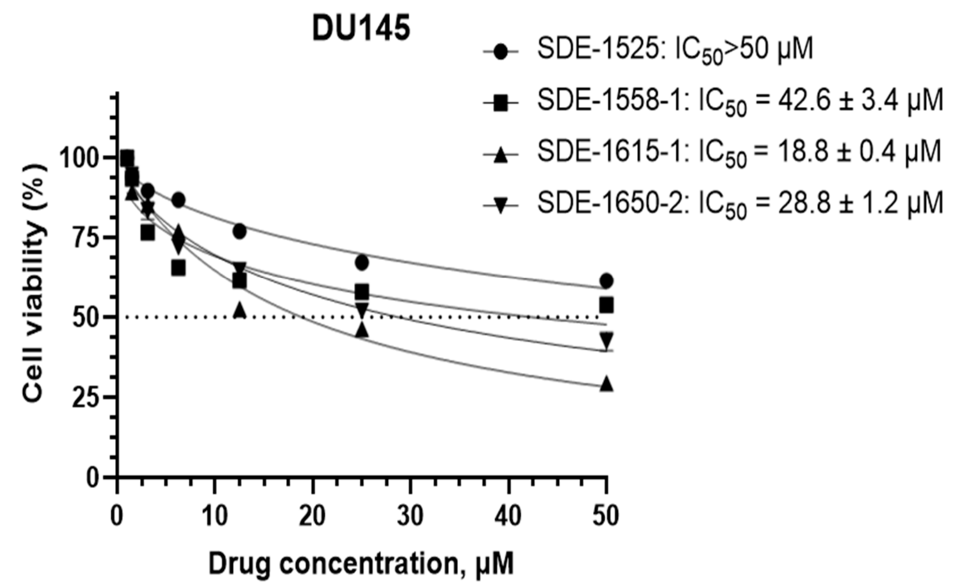
</td></tr>
<tr><td>Normal cell</td><td>HEK-293</td><td>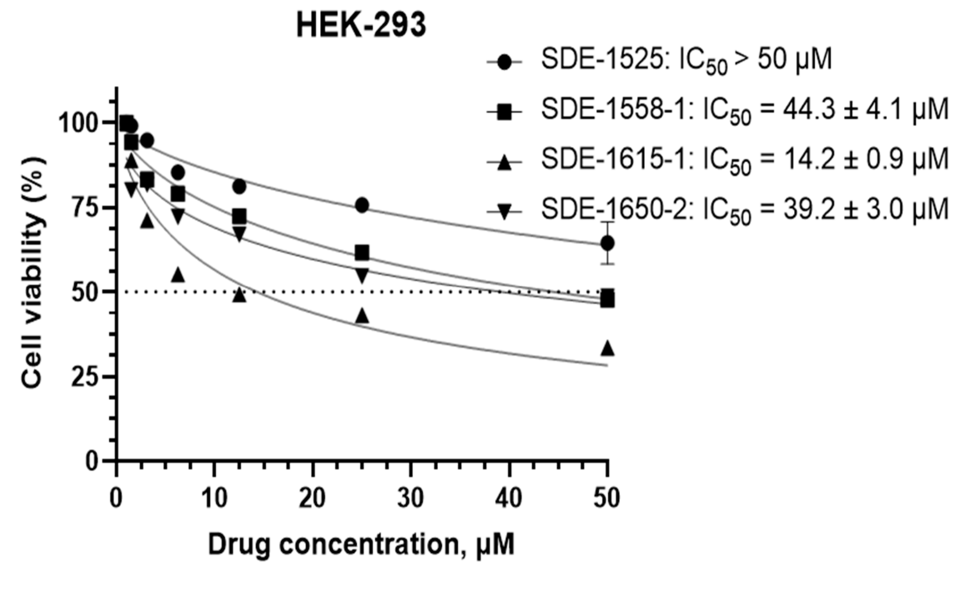
</td></tr>
</table>
